# AI-based digital image dietary assessment methods compared to humans and ground truth: a systematic review

**DOI:** 10.1080/07853890.2023.2273497

**Published:** 2023-12-07

**Authors:** Eleanor Shonkoff, Kelly Copeland Cara, Xuechen (Anna) Pei, Mei Chung, Shreyas Kamath, Karen Panetta, Erin Hennessy

**Affiliations:** a School of Health Sciences, Merrimack College, North Andover, MA, USA; b Friedman School of Nutrition Science and Policy, Tufts University, Boston, MA, USA; c School of Engineering, Tufts University, Medford, MA, USA; d ChildObesity180, Tufts University, Boston, MA, USA

**Keywords:** Artificial intelligence, machine learning, nutrition assessment, nutrition surveys, food images

## Abstract

**Objective:**

Human error estimating food intake is a major source of bias in nutrition research. Artificial intelligence (AI) methods may reduce bias, but the overall accuracy of AI estimates is unknown. This study was a systematic review of peer-reviewed journal articles comparing fully automated AI-based (e.g. deep learning) methods of dietary assessment from digital images to human assessors and ground truth (e.g. doubly labelled water).

**Materials and Methods:**

Literature was searched through May 2023 in four electronic databases plus reference mining. Eligible articles reported AI estimated volume, energy, or nutrients. Independent investigators screened articles and extracted data. Potential sources of bias were documented in absence of an applicable risk of bias assessment tool.

**Results:**

Database and hand searches identified 14,059 unique publications; fifty-two papers (studies) published from 2010 to 2023 were retained. For food detection and classification, 79% of papers used a convolutional neural network. Common ground truth sources were calculation using nutrient tables (51%) and weighed food (27%). Included papers varied widely in food image databases and results reported, so meta-analytic synthesis could not be conducted. Relative errors were extracted or calculated from 69% of papers. Average overall relative errors (AI vs. ground truth) ranged from 0.10% to 38.3% for calories and 0.09% to 33% for volume, suggesting similar performance. Ranges of relative error were lower when images had single/simple foods.

**Conclusions:**

Relative errors for volume and calorie estimations suggest that AI methods align with – and have the potential to exceed – accuracy of human estimations. However, variability in food image databases and results reported prevented meta-analytic synthesis. The field can advance by testing AI architectures on a limited number of large-scale food image and nutrition databases that the field determines to be adequate for training and testing and by reporting accuracy of at least absolute and relative error for volume or calorie estimations.

## KEY MESSAGES

- These results suggest that AI methods are in line with – and have the potential to exceed – accuracy of human estimations of nutrient content based on digital food images.
- Variability in food image databases used and results reported prevented meta-analytic synthesis.
- The field can advance by testing AI architectures on a limited number of large-scale food image and nutrition databases that the field determines to be accurate and by reporting accuracy of at least absolute and relative error for volume or calorie estimations.
- Overall, the tools currently available need more development before deployment as stand-alone dietary assessment methods in nutrition research or clinical practice.

## Introduction

Artificial intelligence (AI) methods such as deep learning [1] are now being used to further nutrition science [2–4], with applications to food classification, image- and non-image-based assessment of dietary intake, and identification of biomarkers of foods or nutrients [2,4], among others. Additionally, the nutrition field has also seen development and growth in investigating digital images (DIs) in dietary assessment^5^ .DIs have been used successfully to supplement other methods of evaluating dietary intake – such as enhancing written food records or image-assisted 24 h dietary recall – and can be a stand-alone tool when images are high-quality and adequately capture all foods pre-consumption^5^ .This review investigates literature at the intersection of AI and DI dietary assessment.

AI-based systems may revolutionize capabilities for accuracy, speed, or complex pattern recognition; their use in clinical healthcare settings for vision-based evaluation may be more accurate than human doctors for detection of cancerous pulmonary nodules or fractured wrists on radiology scans^6^ .In clinical nutrition, AI techniques have been used to aid in diet optimization for health conditions, food image recognition, risk prediction, self-monitoring of dietary intake, precision nutrition, and analyzing links between diet patterns and health outcomes [1,2]. AI image-based processes have been tested for assessing nutrient intake in hospitalized patients [7], estimating protein content of supplement powders [8], fully-automating calorie intake estimation [9], estimating carbohydrate content of foods for diabetics [10], and estimating children’s fruit and vegetable consumption [11]. There is a significant range of potential applications for this technology in the nutrition field.

One specific line of research has been to evaluate the potential of AI dietary assessment methods for self-monitoring dietary intake, which is consistently found to be associated with weight loss [12] but people often underestimate dietary intake (especially energy) [13,14], particularly those with obesity [15]. Digital image dietary assessment has been shown to reduce underreporting [16]. Technology-supported self-monitoring may improve dietary changes, adherence, and anthropometric outcomes compared to other methods [17,18]. Findings from the burgeoning field of AI-assisted dietary assessment suggest potential to estimate portion size [19,20], carbohydrate content [21], and calorie content of foods [22,23].

AI methods can be built to rely on some human input for algorithm completion (i.e. ‘semi-automated’ or ‘semi-automatic’), or they could aim to go from digital images to estimated diet or nutrition-related information through a fully-automated or automatic process (i.e. independent of the user’s input) [24,25]. In contrast to other recent work (e.g. Doulah et al. [26]), we focus on fully-automatic processes in this review. Steps of the fully automatic process include food segmentation, classification, volume and nutrient estimation. Recent reviews have found that convolutional neural networks (CNNs) are frequently used across steps of the fully automatic process in dietary assessment using images [27,28], and that they performed better on large publicly available datasets than other approaches (e.g. hand-extracted features fed to a traditional machine learning classifier) [27]. Three recent reviews have examined AI-based dietary assessment based on images [24,25,27]. Dalakleidi et al. [27] found that estimating volume, calorie, and nutrients were the least-researched portion of the process and noted challenges such as lack of depth information and annotated datasets. Wang et al. [24] reviewed and discussed many vision-based methods for automatic dietary analysis and found the need for a large-scale benchmark dataset. Hochsmann and Martin [25] reviewed image-based dietary assessment methods that included human and AI approaches; they concluded that human management of input from digital images seems necessary to ensure accurate results at this stage of development. Finally, Kaur et al.’s [29] systematic review summarized a variety of deep neural networks that have been used to analyze digital images for nutrient information and identified some common approaches and databases.

Despite these reviews, what remains unknown is the accuracy of the full array of fully automated AI-techniques at estimating energy consumed compared to best-practices (e.g. weighed plate waste). AI shows promise as a route for meeting the critical need for accurate, low-burden dietary assessment. AI-based digital image methods have the potential to reduce burden when they require fewer tools (e.g. smartphone instead of a heavy scale). Critical review of this array of techniques is needed, particularly as these types of technologies become increasingly deployed for use by the public. For instance, in some countries, smartphones are already on the market equipped with camera technology purported to provide feedback about calorie content of foods [30,31]. The objective of this study was to conduct a systematic review of the literature comparing fully automated AI-based methods of dietary assessment from digital images to human assessors and to ground truth.

## Materials and methods

The key question of this systematic review is: How similar are AI techniques to human beings or ground truth when analyzing digital images of food for diet related features? Study methods followed the National Academy of Medicine’s Standards for Systematic Reviews [32] and the Cochrane Handbook for Systematic Reviews of Interventions [33]. Results are reported according to the Preferred Reporting Items for Systematic Reviews and Meta-Analyses (PRISMA) statement [34]. A study protocol was developed prior to data extraction (available on request). This review was not registered.

### Data sources and searches

Search strategies limited results to articles reporting on the use of AI methods to process and analyze digital food images. Search terms included ‘artificial intelligence’ and related terms (e.g. deep learning, machine learning, neural network), terms related to image coding or classification, and common dietary assessment terminology (e.g. nutrition assessment, food analysis, food intake, volume, quantity, portion, calories) (see Supplemental eTable 1**)**. Database searches were conducted in Ovid MEDLINE® (1946 to May 26, 2023) and Cochrane Central Register of Controlled Trials (1991 to May 26, 2023), Embase (1966 to May 26, 2023), and Web of Science Core Collection (1900 to May 27, 2023) plus reference mining in related review articles. Only articles published in peer-reviewed journals were included. For relevant conference presentations, we performed manual searches for peer-reviewed publications and affiliated labs.

### Study selection

After removing duplicate citations, two independent investigators screened all titles, abstracts, and full-text articles in Covidence online software based on eligibility criteria outlined in Supplemental eTable 2. Disagreements were resolved by consensus or a third investigator. Articles were eligible if they reported comparisons of AI methods to digital food image assessment by human assessors (typically dietitians) or to ground truth as defined by the original study. Supplementary eTable 3 presents excluded articles and reasons.

### Data extraction

Two independent investigators extracted study characteristic and outcome data and resolved discrepancies by discussion, or a third investigator resolved them. Relative errors for AI-estimated energy or nutrients were extracted or calculated (|actual – estimated|/actual)*100) from published results when possible to allow for comparability across papers (in contrast to absolute error, which was paper-specific). Thus, the relative errors represented the difference between AI-estimated results and the ground truth. For example, if a paper defined ‘ground truth’ for calories in a hamburger as the weight (g) of the burger multiplied by the calories per gram, then a relative error of 1% would mean that the AI system’s estimation of calories differed from the weighed plate waste measurement of calories by only 1% (indicating high accuracy). When multiple relative errors were presented, the result from the best-performing or main proposed version of the AI architecture was extracted (see Supplemental eTable 4). If available, we extracted reported average relative errors (over various food types or methods) and highest and lowest reported individual relative errors (from the best-performing architecture or iteration).

### Risk of bias

To our knowledge, no tool exists to assess risk of bias for the types of AI studies included in this review where system input – not sample size or study design – are considered the main source of bias. Therefore, AI engineers were consulted regarding potential sources of bias at any stage of the image analysis or accuracy evaluation processes. Two independent investigators documented these potential biases for each study, and discrepancies were adjudicated by a third investigator (see Supplemental eTable 5).

### Data synthesis

The main outcome was accuracy of AI methods to estimate dietary components. A narrative synthesis of all included studies and summary tables are presented. Due to high heterogeneity in reported effect measures and study methods, data were not appropriate to perform meta-analyses [33]. Instead of calculating an overall effect size, forest plots of individual study results are presented to facilitate qualitative synthesis for papers with relative error for calories or volume. Plots were created using Stata/SE 17.0 for Windows.

## Results

**Figure 1.**
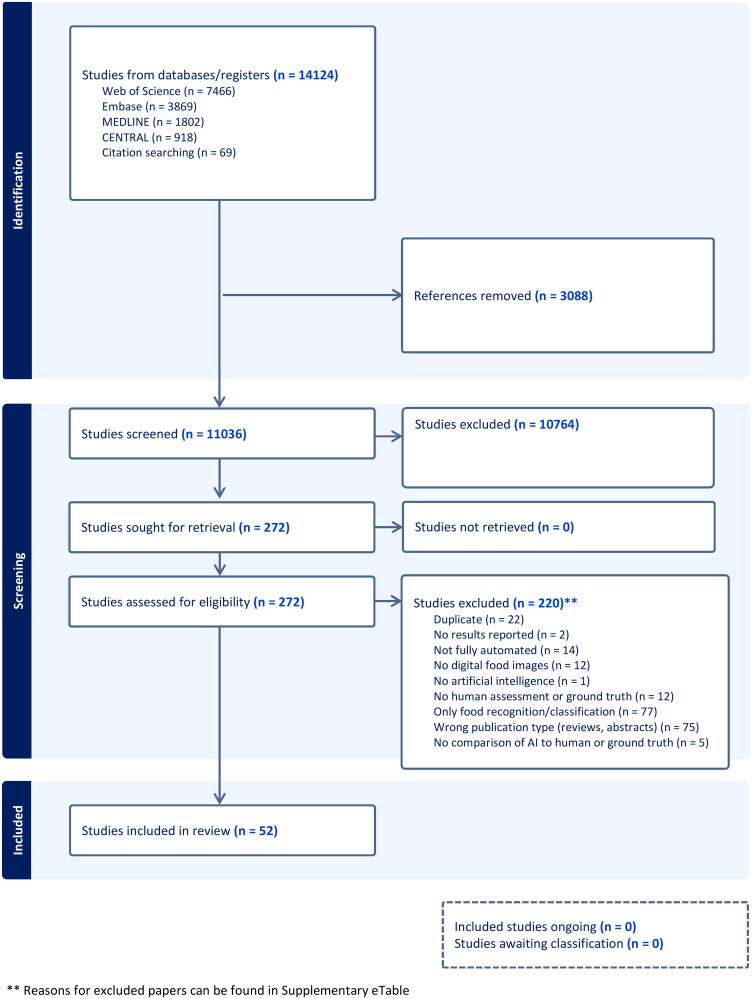
AI SR project.

The literature selection process is summarized [35] in Figure 1. Altogether, 52 papers published from 2010 to 2023 (44 papers published after 2015) were included [7–11,19–23,36–77]. Study authors were affiliated with institutions across 17 countries, with 35% in the United States (see Table 1). Studies received funding from government (44%), private (33%), education (25%) or other sources (2%), with 33% reporting no sources of external funding. The most commonly reported outcomes were estimated calories (52% of studies) and volume (40%). Most papers (81%) compared results from AI methods to ground truth alone. The two common definitions of ground truth were calculation using nutrient information [7,9, 10,22,23,39,41,42,45,46,49,51–53,55,57,60,62–64,67,71,73–76] (e.g. a database with nutrition information, such a a USDA database; 51%) and weighed food (27%) [7–10,38,39,42,44,48,52,56,58,66,72] (see Table 1 and Supplemental eTable 4). No studies directly measured energy content of foods (i.e. bomb calorimetry).

**Table 1.** Summary of study characteristics.

Characteristic	*N* (of studies)	% of 52 studies^a^
Authors’ country affiliations		
USA	18	35%
China	10	19%
India	9	17%
Switzerland	8	15%
Japan	6	12%
Australia	2	4%
Indonesia	2	4%
Malaysia	2	4%
UK	2	4%
South Korea	2	4%
Italy	1	2%
Saudi Arabia	1	2%
Bangladesh	1	2%
Canada	1	2%
Kuwait	1	2%
New Zealand	1	2%
Thailand	1	2%
Source of funding		
Government	23	44%
Private (hospital, non-profit, corporate, etc.)	17	33%
Reported no external source or did not report a source	17	33%
Education (e.g. University)	13	25%
Other (e.g. crowdfunding)	1	2%
Food measure estimated by AI		
Calories	27	52%
Volume	21	40%
Carbohydrate	12	23%
Weight or Mass	11	21%
Protein	11	21%
Fat	8	15%
Area	5	10%
Other (e.g. nutrient, ingredient)	9	17%
Fiber	4	8%
Portion/serving/count	1	2%
Definitions of ground truth		
Calculation using nutrient information	26	51%
Weighed food	14	27%
Other	14	27%
Water displacement	9	17%
Manual measure / digital image annotation / 3-D model	9	17%
Methods of validation (comparators for AI)^b^		
Ground truth alone	42	81%
Ground truth and human assessor	8	15%
Ground truth and calorie app	1	2%
Human assessor alone	1	2%
Types of results/tests reported		
Relative error (i.e. accuracy %, error %)	32	62%
Absolute error	18	35%
Correlation coefficient / Coefficient of variation	16	31%
Other (e.g. actual and estimated calories but not error)	12	23%
Bland-Altman	8	15%
t-test	6	12%
Root Mean Square Error of Approximation or Mean Square Error	5	10%
Regression line	4	8%
Food or nutrient data source		
Health organization, hospital, or national/international food database	15	29%
United States Department of Agriculture database	13	25%
Not applicable (e.g. examined volume)	12	23%
Other (e.g. cooking website, corporate website)	12	23%
Not reported	6	12%
Tool used to capture images		
System included at least one digital camera or smartphone	30	59%
Not applicable	15	29%
Other (e.g. depth camera, wearable device)	6	12%
Not reported	1	2%
Source of images		
Newly captured images	25	48%
Pre-existing images (not newly captured)	16	31%
Both newly captured and pre-existing images	10	19%
Not reported	1	2%
Use of a fiducial marker or indicator of a known size		
No	22	42%
Yes	14	27%
Not reported	9	17%
Other	5	10%
Mixed (yes and no, depending on experiment or dataset)	2	4%
Process of AI estimation		
Volume to nutrients/energy	20	38%
Not applicable	9	17%
Other	9	17%
Direct estimate of nutrients from image	5	10%
Portion to nutrients (e.g. 4 fries)	5	10%
Mass to nutrients	3	6%
Weight to nutrients	3	6%
Type of Convolutional Neural Network		
Did not specify type of CNN	17	33%
Not applicable (i.e. did not report using a CNN)	11	21%
Other	10	19%
ResNet or DenseNet	6	12%
Mask R-CNN or other R-CNN	6	12%
Inception or Xception	6	12%
Multi-task CNN	3	6%
VGG	3	6%
Deep CNN	2	4%

AI: artificial intelligence.

^a^Totals sum to greater than 100% if more than one characteristic appeared in one study, such as authors from one paper affiliated with research centers in more than one country.

^b^In two studies, dietitian’s assessment were the ground truth definition [11, 76].

### Databases for nutrient information and food images

Twenty-five percent (25%) of studies used a USDA database to determine nutrient information, and a health organization, hospital or national/international food database was the source for another twenty-nine percent (29%). Approximately half (48%) of studies captured new food images,; thirty-one percent (31%) used pre-existing images (such as downloaded from the internet), and nineteen percent (19%) used a combination of new and pre-existing images. Twenty-two (22) papers reported using named food image databases, with some papers reporting more than one. Databases included: Angles-13 [43], Food-101 [60,67,73], Plates-18 [43], American Calorie Annotated [23], CALO Mama [70], Calorie-Annotated Food Photo Dataset [22,23], CFNet-34 (ChineseFoodNet + new images) [66], ChinaFood-100 [60], ChinaMarketFood-109 [60,67], FLD-DET [49], FLD-469 [49], Food2k [76], Food-101 [60,67,73], FoodLog [49,72], Fruit 360 [73], FRUITS [69], ImageNet [7,9, 51,67,68,76], ImageNet-1000 [23,46,60,67,68,76], Inselspital [10,41–43], Japanese Calorie-Annotated Food Photo Dataset [23], JISS-22 [49], JISS-DET [49], Korea Food Image database [64], MADiMA [55], fast food database [55], Meals-14 [43], Meals-45 [43], Nasco Life/form Food replica [19], Nutrient Intake Assessment Database [7], Nutrition5K [76], Rakuten18 [51], UECFood-100 [60,67], UECFood-256 [60,67], UNIMIB2016 dataset [46,68,71], VFDL-15 [62], VFDS-15 [62], and Yale-CMU-Berkeley object set [47]. Very few AI systems were tested using the same image datasets: six (6) papers used ImageNet or Image-Net-1000; Inselspital was used in four (4); UNIMIB2016, Food-101, UECFood-100/UECFood-256, Calorie-Annotated Food Photo Dataset, ChinaMarketFood-109 and FoodLog were used in two (2).

Notably, the most frequently reported tools for capturing images were smartphones or digital cameras, with 59% of studies using them either alone or in combination with other tools such as structured light systems, depth sensors, infrared projectors, or wearable devices. Some food images included a fiducial marker (27%) or an indicator of known size (10%) as a reference to aid the AI system in estimating depth for calculating volume in real-world images. This reference scale is established using objects of known dimensions, such as fiducial markers, a photographer’s thumb [39,57], a credit card [10,21,37], the serving container [20], the width of the mobile phone [19], a Rubik’s Cube [54], or superimposing a square grid on the image [45]. The procedure involves physically placing these items on the dining table or in the image. Many studies reported not using a fiducial marker (42%), some varied across iterations within the study (4%) and some did not report either way (17%) (also see Supplemental eTable 6).

### Artificial intelligence methods

Broadly, AI systems aimed to take a digital image as input (e.g. an image of plate with a burger and fries) and provide diet- or nutrition-related information as output (e.g. energy contained in the foods imaged, volume of the burger in the image). They used a variety of methods to achieve this aim. Many had stepwise approach: processing the image, identifying and classifying foods, segmenting the image into specific areas containing specific foods, and linking that information to a nutrition database to determine nutrition facts about the foods contained in the image. Most papers took unique approaches (see Supplemental eTable 6).

In many papers, the AI system estimated nutrient or energy content by first estimating the volume of food contained in the image and then calculating nutrients/energy (38%; see Table 1). A majority of papers (79%) reported using some type of deep learning based Convolutional Neural Network (CNN) (see Table 1 and Supplemental eTable6). Deep-learning methods are those that can take raw data (such as an image, as an array of pixel values) and identify how to represent it at successively higher levels of abstraction [78]. Neural networks ‘learn to map a fixed-size input (for example, an image) to a fixed-size output (for example, a probability for each of several categories)’ and convolutional neural networks can process information from grid like data, such as image and videos. They have the capability to automatically learn and extract hierarchical features from the data, thus making them more powerful in computer vision [78]. Various deep CNN architectures were used for food detection and classification, such as deep neural network [55,62], feed-forward NN classifier [45], mask R-CNN [47,55], MTCnet with and without CRF (Conditional Random Field) [7], multi-task CNN or contextual network [7,22,51], adaptive neural networks [11], Whale Levenberg Marquardt Neural Network classifier [46], ResNet50 [49], and VGG-16 [22,23].Twelve studies (34%) used a 3-D volume or model reconstruction for food volume estimation [7,10,20,37,40–43,47,50,55,56], in which the AI architecture creates an internal representation of a three dimensional model that can be output and viewed by the human (appearing like a picture or video). Other approaches included rotating a contour around a central axis to construct a ball-shaped model [21], using stereo matching or stereo image [41,43,48,55], random sample consensus (RANSAC) [41,43,52], Speeded Up Robust Features (SURF) [41,43,44], surrounding box [48], structured light systems [50], or virtual reality technology to aid in estimating size compared to a cube [19].

A substantial dataset is typically necessary for conducting AI training with CNNs. As summarized above, some researchers used existing datasets to train and evaluate their AI models, while others created entirely new and extensive datasets. These datasets were designed for the purpose of training and testing various AI methods. To enhance dataset diversity and address the data-intensive nature of AI training, some researchers created smaller datasets and merged them with existing ones. In fact, a majority of the studies (67%) expanded their training datasets for deep learning methods that require substantial data by either developing entirely new extensive datasets or combining new ones with pre-existing ones. As described, very few named databases were used for more than one study. The most frequently used were ImageNet or ImageNet-1000, which was used for six tests (across all studies/papers), and Inselspital which was used for four. Importantly, this lack of database overlap prohibited meta-analysis, which requires close alignment across studies in order to pool data.

### Types of results reported

Supplemental eFigure 1 shows the percent of studies reporting the most common accuracy results by diet-related component. The types of analyses conducted and results reported varied widely (e.g. averaging across multiple types of foods vs. within food types only; a small number of images from a limited set of foods such as an apple and banana vs. numerous images of complex mixed dishes, such as curry). Because studies defined ground truth differently, the same effect measure (e.g. relative error) did not consistently represent the same comparison. As shown in Table 1, the most commonly reported diet-related outcomes were volume, calories/energy, carbohydrates, weight/mass and protein. The most frequently reported results for these were relative error for volume (40%) and calories/energy (31%), and absolute error for volume (21%) and calories/energy (25%) (see Supplementary eFigure 1). Relative error was reported for a lower percentage of studies reporting on carbohydrates (12%), weight/mass (6%) or protein (6%). Other reported measures included Bland-Altman analysis; actual and estimated values (e.g. volumes, nutrients); and tests of mean differences.

### Relative error of calorie and volume estimations from AI architectures

**Figure 2.**
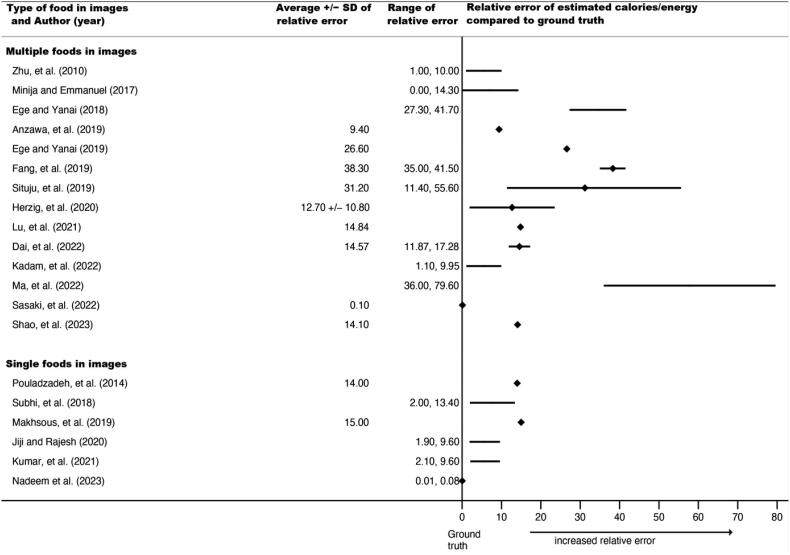
Forest plot of average relative errors and range of relative errors for AI-estimated calories by year and type of food items in image (*n* = 20 papers). ♦ indicates a reported or calculated average relative error, except for Nadeem et al, 2023, for which the range was too small to appear using a line, so it indicates the range. **−** indicates the range between the lowest and highest individual relative errors for the best-performing or proposed AI architecture

**Figure 3.**
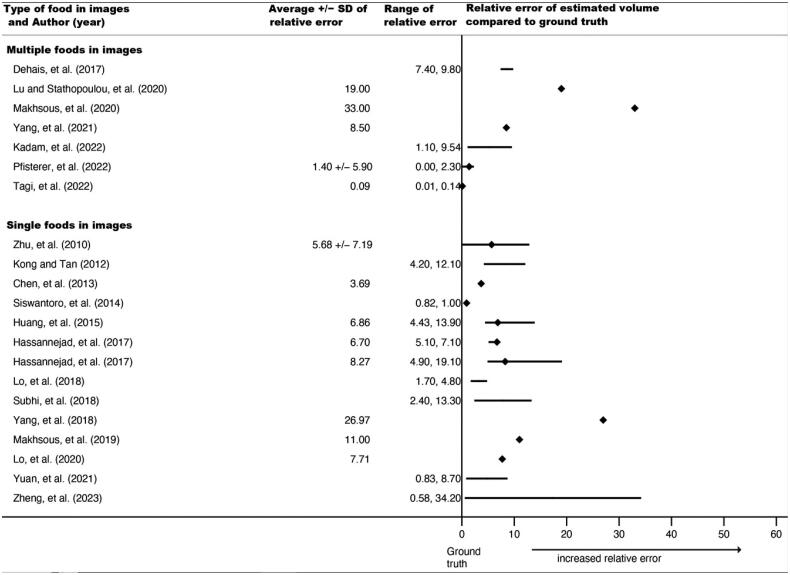
Forest plot of average relative errors and range of relative errors for AI-estimated volume by year and type of food items in image (*n* = 22 papers). ♦ indicates a reported or calculated average relative error; except for Siswantoro et al., 2014, for which the range was too small to appear using a line, so it indicates the range. **−** indicates the range between the lowest and highest individual relative errors for the best-performing or proposed AI architecture

Forty-two relative errors could be extracted or calculated from published results for calories, volume, or both in 36 studies (69%). Some studies planned *a priori* to train the AI system until a certain percent accuracy was reached, so extracted relative errors do not necessarily represent the upper limits of a system, just accuracy when results were published. As noted, relative errors could not be directly compared or synthesized because they were based on different types of food images as input into the AI architectures (e.g. a single apple vs. a plate of mashed potatoes with meatloaf). Thus, Figures 2 and 3 display average relative errors (for calories and volume, respectively), ranges of individual relative errors, and standard deviations (where reported) grouped by type of food in images (single vs. multiple foods), but no overall pooled relative error.

For calories, reported average relative errors (e.g. over different types of foods tested within the same study) ranged from 0.10% to 38.3%; the lowest individual relative error (from the range over all papers) was 0.00% and the highest was 79.6%. Six of these 20 relative errors (30%) were from AI estimates using single food images (vs. multiple foods). For volume, reported average relative errors ranged from 0.09% to 33%; individual relative errors had a low of 0.00% and a high of 34.20%; fifteen of these 22 (68%) were from single food images. Average relative errors and ranges tended to be smaller for the single food images compared to multiple food images, suggesting that AI architectures more closely approximated ground truth when images contained simpler food. The range of reported average relative errors for calories and volume were comparable, if slightly lower for volume, which had a higher proportion of single food images. This suggests relatively similar accuracy for calories and volume.

### Food image characteristics as potential sources of bias

Table 2 reports descriptive statistics about food image characteristics that we identified as potential sources of bias (see Supplemental eTable 5). Methods of reducing bias included having a trained person take the images (50% of studies); controlling the setting (33% ‘mostly controlled’), food layout (‘somewhat controlled’ 37%) or lighting (‘somewhat controlled’, 31%); and reporting on image features (31% on pixels or resolution; 31% on some aspect of image quality). Types of images were roughly equally divided among standardized foods (38%; e.g. chain restaurant), non-standardized (27%; e.g. home-prepared), and other (35%). Images for most papers contained multiple food items, either solely (35%) or in addition to images of single food items (38%).

**Table 2.** Summary statistics for food image database characteristics.

Characteristic	*N* (of studies)	% of 52 studies
Image acquisition		
Active: Taken by researcher/study staff member or trained study participant	26	50%
Passive: Automatically detected by camera	9	17%
Not applicable	9	17%
Active: Not clear who took the image	7	13%
Not reported	1	2%
Image setting^a^		
Mostly controlled (e.g. Real-world with clear table and solid background)	17	33%
Somewhat controlled (e.g. Real-world with some objects on table OR background noise)	11	21%
Highly controlled (e.g. Laboratory with grid-paper)	9	17%
Other / indeterminate or mixed	9	17%
Not at all controlled (e.g. Real-world with some objects on table AND background noise)	6	12%
Image lighting^a^		
Somewhat controlled (e.g. Real-world with consistently good lighting)	16	31%
Highly controlled (e.g. Laboratory with staged lighting)	9	17%
Not at all controlled (e.g. Real-world with inconsistent lighting)	8	15%
Mixed or indeterminate lighting (e.g. Two or more lighting environments included)	7	13%
Mostly controlled (e.g. Real-world with professional lighting)	7	13%
Was image quality mentioned in the paper?		
Reported on some aspect of image quality	16	31%
Reported standard image pixels, or resolution	16	31%
Did not report on image quality	7	13%
Reported using only high-quality images	6	12%
Reported excluding low-quality images from analyses	2	4%
Type of foods in image		
Standardized (e.g. chain restaurant, cafeteria, packaged foods)	20	38%
Other (e.g. mix of standardized & non-standardized)	18	35%
Non-standardized (e.g. home-prepared, non-chain restaurant, buffet)	14	27%
Single vs. multiple food items in the image		
Single and multiple food item images	20	38%
Multiple separate food items (e.g. plate with burger and fries)	18	35%
Single food	13	25%
Unclear	1	2%
Food layout		
Somewhat controlled (e.g. arranged, but with some item overlap/occlusion)	19	37%
Highly controlled (e.g. laid out with clear separation between multiple items)	13	25%
Mostly controlled (e.g. arranged, but items are close together on a plate/tray)	10	19%
Other / indeterminate / mixed	7	13%
Not at all controlled (e.g. not arranged and/or much item overlap/occlusion)	3	6%

^a^If image conditions were not described, investigators from this study interpreted setting and/or lighting from images included in the publication.

## Discussion

In recent years, significant interest has emerged in investigating use of AI methods for conducting accurate dietary assessment using digital food images [3]. This review found 35 papers (studies), most published since 2015, investigating AI approaches using a wide variety of tools and techniques. Calories, food volume, and carbohydrate content were the most frequently reported dietary components, and absolute error and relative error were the most common accuracy indicators. AI estimations had a wide range of average relative errors compared to ground truth, with relatively comparable ranges between calories and volume (approximately 0.10% to 38% for calories and 0.09% to 33% for volume). This was not surprising, as calories and other nutrients were often calculated from AI estimated volumes. No papers used bomb-calorimetry, a gold standard ground truth method to measure energy in nutrition science; but weighed plate waste and nutrient information from posted restaurant menus (where some papers got their calorie information) have been found to be strongly correlated with bomb-calorimetry methods [79,80]. Most papers used a convolutional neural network. USDA databases (https://fdc.nal.usda.gov/index.html) were the source of nutrient information in a fourth of studies, but only one food image database was used by more than one research group (ImageNet/ImageNet-1000). Image quality – a potential source of bias – was discussed in about a third of papers. Overall, the breadth of work is impressive, and AI tools are reaching a high degree of accuracy; however, the published literature contains substantial variability in food image databases used and types of analytical results reported, so findings at this stage of development cannot be synthesized.

AI architecture estimations ranged from 62% to 99% accurate (calculated: 1 - % error) over calories and volume measures when examining results that were averaged across images of multiple foods or food types (Figures 1 and 2). Images of simpler foods (i.e. single item, like an apple) appeared to co-occur with lower relative error rates. The ranges for highest error reported were slightly higher for calories (vs. volume), but a larger proportion of those images were of multiple food images, which may have been harder for AI systems than single food images and not indicative of greater accuracy for volume. Images with only one, simple food (like an apple) may have fewer ‘distractions’ for computer vision processes, reducing the likelihood that the AI system misclassifies areas in the image. In contrast, images with mixed dishes (such as curries) or plates with a variety of overlapping foods of varying heights or with unclear boundaries present higher risk for classification or segmentation errors.

For many studies, absolute or relative errors were directly reported or could be calculated from reported data. The results for AI appear to be within the range of accuracy levels from human coders of digital images in other studies [25]. As noted, in contrast to expectations, only one paper directly compared accuracy of AI methods to humans alone; and only 8 papers used human assessors at all, indicating an area open for research. One recent review of digital image dietary assessment methods reported that percent error of human methods ranged between 30% underestimation to 1% overestimation, indicating accuracy of 70% to 99% across various outcomes and ground truth methods (e.g. energy intake, servings, weighed food, doubly labeled water) [25]. Studies using human coders tend to report correlations between ground truth and human estimations for weight (g) and calories (kcal); and they tend to use Bland-Altman plots to display associations across varying levels of leftover food. While all papers included in this review reported some type of analytic comparison between AI and ground truth, only some reported correlations. For results from AI architectures to be more directly comparable to results from human coders, AI researchers could report the correlation; and they could examine the AI tool’s ability to identify nutrients consumed by estimating both full portions and leftover unconsumed food, back-calculating actual consumption; further, they could display results with Bland-Altman plots.

As noted, the relative errors reported in the included papers reflected a variety of AI methods and comparisons. Researchers had varying aims or needs, such as optimizing for computing space or processing time vs. optimizing for the lowest relative error. Extracted results represent the peer-reviewed literature but not necessarily the upper limits of accuracy. But to contextualize the current findings, a 99% accurate AI-based calorie management tool would lead to under- or over-estimation of calorie intake by only about 20 calories per day (based on a 2,000-calorie diet). If consistent over a year, those errors would equate to 7,300 calories per year or about 2 pounds. A tool that was 62% accurate would be off by 760 calories per day, or 277,400 calories (79 pounds) per year. These numbers drastically oversimplify the complex processes of appetite and food intake self-regulation, and omit important components of energy balance (such as physical activity), but they convey a sense of the scope of the impact of the current ranges of relative error. As AI-based dietary assessment tools develop, it will be important to determine what features of the food images, AI architecture, and end-users lead to over- and under-estimation of nutrient intake so that user-facing tools can be adjusted for accurate self-management of dietary goals or nutrition research. There are likely trade-offs between end-user burden (e.g. not requiring a fiducial marker, working in inconsistent lighting with overlapping food) and accuracy. It is important to note that this review included only published literature; the accuracy of any applications or camera technology currently on the market [30,31] were not evaluated (unless validity tests had been published).

Convolutional Neural Networks were the most common method of AI estimation, which aligns with recent research finding that CNN-based models are most frequently used [27]. We also found that most studies are testing AI systems on different food image datasets, highlighting the need for a large-scale benchmark food image dataset [24]. The current review specifies characteristics that would be important for such a dataset and recommends analytic techniques and reporting guidelines that would allow comparisons across nutrition and engineering fields, and meta-analytic synthesis. Recent reviews envision AI as being on the precipice of tremendous leaps in accuracy, timeliness, and clinically meaningful nutrition outcomes [1,2, 4]. But they urge the nascent science to communicate in broader terms to connect across audiences, catch up to the AI advances made in medicine, and identify those areas where AI can accelerate progress vs. those that will always rely on traditional (human) judgement [1,2, 4]. As AI tools become sufficiently developed, coordination will need to occur with clinical fields as well (e.g. physicians, dietitians) for management of nutrition-related chronic disease.

The heterogeneity we found in results reported precluded meta-analytic synthesis for two reasons. First, for a valid comparison between two AI architectures both would need to have the same sets of databases input into the system. Yet, few databases were used in more than one study. This emerging field would benefit from agreement on the characteristics needed for a valid food image database for training and testing and from selecting a limited number of ‘gold standard’ databases. Potential valuable features might include a wide variety of food types (e.g. separate foods like individual fruits, mixed foods like curries, cultural variety); high image quality (including pixels, resolution); lighting condition; a variety of settings (e.g. chain restaurant, home table, mall food court); a variety of camera angles; and potentially the inclusion of a consistent fiducial marker or size referent (e.g. photographer’s thumb). One recent review suggested that MADiMA [55] and Nutrition5K [81] seemed the most comprehensive datasets currently available for this type of use but noted that they did not have ‘eastern style’ foods [24]. (Authors described ‘western style’ foods tending to be served separately, while ‘eastern’ foods were mixed; the study was conducted in China). Researchers should also consider making their own databases publicly available so other researchers can compare AI performance.

Second, even if the inputs to the AI systems identified in this review had been the same, the outputs differed. The types of analyses conducted to determine system ‘accuracy’ varied across papers in at least two ways. First, the statistical methods differed. Absolute error was the most frequently reported numeric result, but over 10 types of indicators were reported. Second, the definition of ground truth varied. So, the absolute error in one paper may have represented a comparison between AI and a dietitian coding the digital image, another may have compared AI to plate waste weighed on a household food scale, and a third may have compared it to ‘one serving’ from a nutrition database. An argument could be made that pooling these absolute values would be conceptually similar to pooling effect sizes across different populations (e.g. adolescents, seniors), and heterogeneity could be calculated and examined. In this case, that could not be done because of different system databases; but beyond that, there is no field-wide ‘gold standard’ for the type of analysis or definition of ground truth. Moving forward, researchers could strive to use a limited set of high-quality food image databases, test AI architectures on food images containing both single and multiple foods, and report overall relative errors (and standard deviations) averaged over many different food types.

This review also revealed a gap in the literature regarding validated tools for assessing risk of bias in these types of studies. As described above, bias could come from inputs into the system: a food image database with low-quality images, a limited set of ‘easy’ images, or a definition of ground truth that was not accurate. We addressed this challenge by evaluating studies on features that may have biased the food classification, segmentation, or dietary estimation process. We selected features such as image setting, content, lighting, and resolution because poor quality would create missing information potentially leading to error in the AI estimation. Results showed variety in the types of environments represented in images (e.g. chain restaurant, home tables) and some degree of control over the image setting and lighting. The majority (65%) reported something related to image quality (e.g. pixels, resolution, blurriness), though definitions of quality varied. We offer this list of characteristics to future researchers as a framework for reporting results in published literature.

In addition to bias resulting from food image database input, recent research raises the possibility that the processing approach itself has the potential to create or reveal other forms of bias, such as a recent finding that an AI system could identify patients’ races from x-ray data [82]. That particular finding has not been replicated, and the way the identification happens is unclear (e.g. geographical history, previously undetectable melanin differences). But the development of these types of technology warrants awareness of ethical, legal, and medical considerations, particularly when the AI tools would be used to manage nutrition-related conditions affecting lifespan or life quality, such as diabetes, obesity, or heart disease [6,83]. In particular, attention will need to be paid to: (a) how AI architectures are trained so they do not learn or magnify unconscious bias from human coders [84,85] and can separate user food choices from historical, social, cultural, or political confounders that could exacerbate health inequities; (b) expanding the universe of populations who engage in user testing and counteracting a reliance on subgroups (such as white females for self-monitoring and weight loss^12^]; (c) being able to explain the processes through which results are obtained [86] while being mindful of potential sources of bias for supervised vs. unsupervised learning; and (d) whether federal approval processes are needed for products released on the market to ensure health claims meet a threshold for demonstrating efficacy. These are only cautions. Overall, there is tremendous potential with AI-based dietary assessment but a significant need to develop and validate tools for assessing bias and comparing study quality.

Strengths of this systematic review included the process (i.e. strict inclusion/exclusion criteria, multiple independent coders) and synthesis of literature addressing an important and timely question. In the Engineering field, scientific results may be reported in preprint form or outside of peer-reviewed journal articles (e.g. conference proceedings). The current systematic review was limited to the published literature to match conventions in nutrition and health sciences. While some preprint reports go through a peer review process, the streamlined descriptions required of abstract submission prohibit detailed explanations of study methods or results for review. We acknowledge that the technology industry moves rapidly, particularly in a market space where commercial products are being created and released for sale, such as cell phones equipped with AI dietary assessment methods [30,31]. But for management of chronic conditions, the speed of innovation should be tempered by the use of testing and validation procedures that protect end user safety. Because this review was limited to peer-reviewed published articles, meaningful results may have been omitted. One recent review [27] relied on an almost entirely different set of papers due to different exclusion criteria. Publication bias is also more difficult to evaluate when substantial literature is in the preprint form. In this review, the procedure for that type of literature was to search for authors’ publications subsequent to the abstract. Articles identified were included in the review. If no follow-up was published, it remains unknown whether the results were later found to be null or invalidated in some way or whether the conference proceedings were considered the end result with no additional need for publishing.

## Conclusions

Significant interest has emerged in recent years in investigating the ability of AI methods to conduct accurate dietary assessment using digital food images. Most studies included in this review reported that their AI system was accurate according to their definition of ground truth. This systematic review found a wide range of food image databases, nutrient information, and types of results reported in the literature. Future researchers should consider using a limited number of databases that the field has determined to be adequate for training and testing. The field should come to consensus on what characteristics are required for a high-quality database (e.g. range of types of foods, number of items in images, lighting conditions and food layouts; linked to nutrient databases known to be accurate). Researchers should also consider reporting at least absolute error and average relative error for AI-based volume and calorie estimations. A validated risk of bias tool needs to be developed so study quality and results can be compared across these types of studies. As the field grows, we recommend focusing on consistent definitions of accuracy and ground truth and conducting cross-field collaboration with end users to identify best practices for using the technology.

## Supplementary Material

Supplemental Material

## Data Availability

The data that support the findings of this study are available from the corresponding author, ES, upon reasonable request.
