# Supplementary material for: AI-based digital image dietary assessment methods compared to humans and ground truth: a systematic review
Source: Ann Med. 2023 Dec 7;55(2):2273497. doi: 10.1080/07853890.2023.2273497 (PMC10836267; doi:10.1080/07853890.2023.2273497)
Supplement: Supplemental Material [file IANN_A_2273497_SM8229.docx]

**List of titles and numbers of supplemental tables and figures**

**eTable 1.** Search strategy

**eTable 2.** Eligibility Criteria

**eTable 3**. Title excluded during the full-text stage of the review with exclusion reasons (ordered chronologically and by first author)

**eTable 4.** Results and key findings for included articles

**eTable 5**. Food image characteristics for each study by year

**eTable 6.** Image-based dietary assessment methods and AI approaches used in included papers

**eFigure 1.** Percent of total papers (n = 52) reporting volume, calories, carbohydrates, weight/mass or protein by type of results reported

This supplementary material has been provided by the authors to give readers additional information about

their work.

**eTable 1.** Search strategy

Search conducted 8/11/2021

Database: Ovid MEDLINE(R) <1946 to July Week 5 2021>

Search Strategy:

--------------------------------------------------------------------------------

1 artificial intelligence/ or Computer Heuristics/ or Expert Systems/ or exp machine learning/ or neural networks, computer/ or exp Image Processing, Computer-Assisted/ or exp Signal Processing, Computer-Assisted/ or Algorithms/ or Automation/ or decision trees/ or Pattern Recognition, Automated/

2 ((artificial or machine or comput* or deep) adj2 (learn* or assist* or transfer or intelligence or network* or model* or approx* or technique* or method* or accur* or analys* or algorithm*)).mp.

3 (autoencod* or "neural network*" or perceptron* or convolutional or ensemble).mp.

4 ((image* or imaging) adj2 (food* or code or coding or digital or classif* or categor* or technolog* or method* or technique* or decod* or "de cod*" or recogn* or approx* or accur* or analys* or segment*)).mp.

5 ("data processing algorithm*" or "object recognition" or "motion estimation" or "3D reconstruction" or "three-dimensional reconstruction" or "three dimensional reconstruction").mp.

6 or/1-5

7 Nutrition Assessment/ or Food Analysis/

8 ((diet* or nutrit* or food* or calori*) adj2 (assess* or analys* or eval* or recall or weigh* or volume or count* or identif* or quanti* or portion* or qualit* or content* or record* or intake* or measur* or estimat* or categor* or approx* or recogn* or accur* or survey* or journal* or instrument*)).mp.

9 7 or 8

10 6 and 9

11 limit 10 to animals

12 10 not 11

Search updated 5/26/2023

Database: Ovid MEDLINE(R) <1946 to May Week 3 2023>

Search Strategy:

--------------------------------------------------------------------------------

1-12 [Same as above]

13 limit 12 to (address or autobiography or bibliography or biography or case reports or clinical conference or comment or congress or consensus development conference or consensus development conference, nih or dataset or dictionary or directory or duplicate publication or editorial or "expression of concern" or festschrift or interactive tutorial or interview or lecture or legal case or legislation or letter or news or newspaper article or patient education handout or periodical index or personal narrative or portrait or randomized controlled trial, veterinary or "research support, american recovery and reinvestment act" or research support, nih, extramural or research support, nih, intramural or research support, non us gov't or research support, us gov't, non phs or research support, us gov't, phs or video-audio media or webcast)

14 12 not 13

15 limit 14 to yr="2021 -Current"

***************************

Search conducted 8/11/2021

Database: EBM Reviews - Cochrane Central Register of Controlled Trials <July 2021>

Search Strategy:

--------------------------------------------------------------------------------

1 artificial intelligence/ or Computer Heuristics/ or Expert Systems/ or exp machine learning/ or neural networks, computer/ or exp Image Processing, Computer-Assisted/ or exp Signal Processing, Computer-Assisted/ or Algorithms/ or Automation/ or decision trees/ or Pattern Recognition, Automated/

2 ((artificial or machine or comput* or deep) adj2 (learn* or assist* or transfer or intelligence or network* or model* or approx* or technique* or method* or accur* or analys* or algorithm*)).mp.

3 (autoencod* or "neural network*" or perceptron* or convolutional or ensemble).mp.

4 ((image* or imaging) adj2 (food* or code or coding or digital or classif* or categor* or technolog* or method* or technique* or decod* or "de cod*" or recogn* or approx* or accur* or analys* or segment*)).mp.

5 ("data processing algorithm*" or "object recognition" or "motion estimation" or "3D reconstruction" or "three-dimensional reconstruction" or "three dimensional reconstruction").mp.

6 or/1-5

7 Nutrition Assessment/ or Food Analysis/

8 ((diet* or nutrit* or food* or calori*) adj2 (assess* or analys* or eval* or recall or weigh* or volume or count* or identif* or quanti* or portion* or qualit* or content* or record* or intake* or measur* or estimat* or categor* or approx* or recogn* or accur* or survey* or journal* or instrument*)).mp.

9 7 or 8

10 6 and 9

Search updated 5/26/2023

Database: EBM Reviews - Cochrane Central Register of Controlled Trials <April 2023>

Search Strategy:

--------------------------------------------------------------------------------

1-10 [Same as above]

11 limit 10 to yr="2021 -Current"

12 limit 11 to medline records

13 11 not 12

***************************

Search conducted 8/11/2021

Embase

Session Results

.......................................................

#13. #10 NOT #11 AND ([embase]/lim OR [pubmed-not-medline]/lim)

#12. #10 NOT #11

#11. #6 AND #9 AND ([animal cell]/lim OR [animal experiment]/lim OR [animal model]/lim OR [animal tissue]/lim)

#10. #6 AND #9

#9. #7 OR #

#8. ((diet* OR nutrit* OR food* OR calori*) NEAR/2 (assess* OR analys* OR eval* OR recall OR weigh* OR volume OR count* OR identif* OR quanti* OR portion* OR qualit* OR content* OR record* OR intake* OR measur* OR estimat* OR categor* OR approx* OR recogn* OR accur* OR survey* OR journal* OR instrument*)):ti,ab,tt,oa,ok,kw,lnk

#7. 'nutritional assessment'/de OR 'food analysis'/de

#6. #1 OR #2 OR #3 OR #4 OR #5

#5. 'data processing algorithm*':ti,ab,tt,oa,ok,kw,lnk OR 'object recognition':ti,ab,tt,oa,ok,kw,lnk OR 'motion estimation':ti,ab,tt,oa,ok,kw,lnk OR '3d reconstruction':ti,ab,tt,oa,ok,kw,lnk OR 'three-dimensional reconstruction':ti,ab,tt,oa,ok,kw,lnk OR 'three dimensional reconstruction':ti,ab,tt,oa,ok,kw,lnk

#4. ((image* OR imaging) NEAR/2 (food* OR code OR coding OR digital OR classif* OR categor* OR technolog* OR method* OR technique* OR decod* OR 'de cod*' OR recogn* OR approx* OR accur* OR analys* OR segment*)):ti,ab,tt,oa,ok,kw,lnk

#3. autoencod*:ti,ab,tt,oa,ok,kw,lnk OR 'neural network*':ti,ab,tt,oa,ok,kw,lnk OR perceptron*:ti,ab,tt,oa,ok,kw,lnk OR convolutional:ti,ab,tt,oa,ok,kw,lnk OR ensemble:ti,ab,tt,oa,ok,kw,lnk

#2. ((artificial OR machine OR comput* OR deep) NEAR/2 (learn* OR assist* OR transfer OR intelligence OR network* OR model* OR approx* OR technique* OR method* OR accur* OR analys* OR algorithm*)):ti,ab,tt,oa,ok,kw,lnk

#1. 'artificial intelligence'/de OR ‘computer heuristics'/de OR 'expert system'/de OR 'machine learning'/de OR 'supervised machine learning'/de OR 'unsupervised machine learning'/de OR 'artificial neural network'/de OR 'image processing'/exp OR 'signal processing'/exp OR 'algorithm'/de OR 'automation'/de OR 'decision tree'/de OR 'automated pattern recognition'/de

Search updated 5/26/2023

Embase

Session Results

.......................................................

No. Query Results

#15. #14 AND [2021-2023]/py

#14. #13 AND ([embase]/lim OR [pubmed-not-medline]/lim)

#13. #12 AND ([article]/lim OR [article in press]/lim OR [conference paper]/lim OR [data papers]/lim OR [review]/lim)

#12. #10 NOT #11

#11. #10 AND [animals]/lim

#1-#10 [Same as above]

***************************

Search conducted 8/16/2021

Web of Science Core Collection (1900 to 08/16/2021)

1 TI=("artificial intelligence" or "Computer Heuristic*" or "Expert System*" or "machine learning" or "deep learning" or "support vector*" or "computer neural network*" or "Image Processing" or "Signal Processing" or "Algorithm*" or "Automation" or "decision tree*" or "Automated Pattern Recognition")

2 AK=("artificial intelligence" or "Computer Heuristic*" or "Expert System*" or "machine learning" or "deep learning" or "support vector*" or "computer neural network*" or "Image Processing" or "Signal Processing" or "Algorithm*" or "Automation" or "decision tree*" or "Automated Pattern Recognition")

3 TS=((artificial or machine or comput* or deep) NEAR/2 (learn* or assist* or transfer or intelligence or network* or model* or approx* or technique* or method* or accur* or analys* or algorithm*))

4 TS=((image* or imaging) NEAR/2 (food* or code or coding or digital or classif* or categor* or technolog* or method* or technique* or decod* or "de cod*" or recogn* or approx* or accur* or analys* or segment*))

5 TS=(autoencod* or "neural network*" or perceptron* or convolutional or ensemble or "data processing algorithm*" or "object recognition" or "motion estimation" or "3D reconstruction" or "three-dimensional reconstruction" or "three dimensional reconstruction")

6 ((((#1) OR #2) OR #3) OR #4) OR #5

7 TS=((diet* or nutrit* or food* or calori*) NEAR/2 (assess* or analys* or eval* or recall or weigh* or volume or count* or identif* or quanti* or portion* or qualit* or content* or record* or intake* or measur* or estimat* or categor* or approx* or recogn* or accur* or survey* or journal* or instrument*))

8 (#6) AND #7

9 (#6) AND #7 and Articles or Review Articles or Data Papers or Early Access (Document Types)

Search updated 5/27/2023

Web of Science Core Collection (1900 to 05/27/2023)

1-9 [Same as above]

10 #6 AND #7 and Article or Review Article or Data Paper or Early Access (Document Types) and 2021 or 2022 or 2023 (Publication Years)

**eTable 2.** Eligibility Criteria*

| **Category** | **Inclusion Criteria** | **Exclusion Criteria** |
| --- | --- | --- |
| Publication Status | Articles published in peer-reviewed journals | Articles not published in peer-reviewed journals, including unpublished data, manuscript reports, abstracts, pre-prints, and conference proceedings |
| Language of Publication | English | Languages other than English |
| Study Design | Cross-sectional studies  Any intervention study:   - Randomized controlled trials - Non-randomized controlled trials - Uncontrolled trials   Prospective cohort studies  Nested case-control studies  Case-cohort studies | Mendelian randomization studies  Retrospective cohort studies  Narrative reviews  Systematic reviews  Meta-analyses  Letters to the editor  Commentaries  Conference proceedings  Abstracts  Case studies or case series |
| Study Participants | Human subjects  No subjects (e.g., plate analysis only) | Animal subjects |
| Data Used for Processing | Digital images of food | No food images (e.g., swallowing sounds, chewing, bites, only food weight) |
| Data Processing Method | **Must include artificial intelligence methods** (AI) including, but not limited to:   - Neural networks - Machine learning - Deep learning - Models and algorithms | No AI  Only semi-automatic AI (i.e., required human input) |
| Comparator | **Must include at least one of these:**   - Comparisons to human assessment of food (e.g., dietitian assessment), **and/or** - Comparisons to ground truth assessment of food   Can also include:   - Other AI comparators - Any other comparators | No comparator  No human or ground truth comparator (e.g., other AI only) |
| Outcomes Assessed by AI | Food assessment including any of these:   - Volume - Proportion (e.g., 70% consumed) - Quantity (number) - Weight (grams) - Nutrients (e.g., vitamin C, kcal) - Item recognition, food classification, or segmentation **PLUS** one of the above (e.g., item recognition AND volume) | No dietary/food content of any kind  **Only** reports item recognition, food classification, or segmentation |
| Outcomes for Data Extraction | Including but not limited to:   - Limits of agreement - Sensitivity and specificity - Estimation error - Pearson or Spearman correlations - Area under the curve (AUC) - Percent accuracy - Positive predictive values | No data showing comparison of AI methods to human assessment or ground truth |

* No restrictions on publication date, age or health status of participants, study duration, study sample size, or study location (country).

**eTable 3**. Title excluded during the full-text stage of the review with exclusion reasons (ordered chronologically and by first author)

| **#** | **First author** | **Year** | **Title** | **Exclusion reason** |
| --- | --- | --- | --- | --- |
| 1 | Sun | 2008 | Determination of food portion size by image processing | Wrong publication type (reviews, abstracts) |
| 2 | Anami | 2009 | Identification of multiple grain image samples from tray | Only food recognition/classification |
| 3 | Boushey | 2009 | Use of technology in children's dietary assessment | No fully automated AI |
| 4 | Martin | 2009 | Quantification of food intake using food image analysis | Wrong publication type (reviews, abstracts) |
| 5 | Puri | 2009 | Recognition and volume estimation of food intake using a mobile device | Duplicate |
| 6 | Anami | 2010 | Influence of Light, Distance and Size on Recognition and Classification of Food Grains' Images | Only food recognition/classification |
| 7 | Chen | 2010 | Toward Dietary Assessment via Mobile Phone Video Cameras | Wrong publication type (reviews, abstracts) |
| 8 | Khanna | 2010 | An Overview of the Technology Assisted Dietary Assessment Project at Purdue University | Wrong publication type (reviews, abstracts) |
| 9 | Weiss | 2010 | Automatic Food Documentation and Volume Computation Using Digital Imaging and Electronic Transmission | No human assessment or ground truth |
| 10 | Woo | 2010 | Automatic portion estimation and visual refinement in mobile dietary assessment | Wrong publication type (reviews, abstracts) |
| 11 | Yue | 2010 | Food volume estimation using a circular reference in image-based dietary studies | Wrong publication type (reviews, abstracts) |
| 12 | Zhu | 2010 | An image analysis system for dietary assessment and evaluation | Wrong publication type (reviews, abstracts) |
| 13 | Zhu | 2010 | The Use of Mobile Devices in Aiding Dietary Assessment and Evaluation | Duplicate |
| 14 | Chae | 2011 | Volume estimation using food speci c shape templates in mobile image-based dietary assessment | Wrong publication type (reviews, abstracts) |
| 15 | Noronha | 2011 | Platemate: Crowdsourcing nutritional analysis from food photographs | Wrong publication type (reviews, abstracts) |
| 16 | Rodrigues | 2011 | Use of food images for evaluating food intake | Wrong publication type (reviews, abstracts) |
| 17 | Shang | 2011 | A mobile structured light system for food volume estimation | Duplicate |
| 18 | Shang | 2011 | A pervasive Dietary Data Recording System | Wrong publication type (reviews, abstracts) |
| 19 | Villalobos | 2011 | A personal assistive system for nutrient intake monitoring | Wrong publication type (reviews, abstracts) |
| 20 | Xu | 2011 | Low Complexity Image Quality Measures for Dietary Assessment Using Mobile Devices | Wrong publication type (reviews, abstracts) |
| 21 | Yao | 2011 | Food Dimension Estimation from A Single Image Using Structured Lights | Wrong publication type (reviews, abstracts) |
| 22 | Zhu | 2011 | Multilevel segmentation for food classification in dietary assessment | Wrong publication type (reviews, abstracts) |
| 23 | Zhu | 2011 | Segmentation assisted food classification for dietary assessment | Wrong publication type (reviews, abstracts) |
| 24 | Almaghrabi | 2012 | A novel method for measuring nutrition intake based on food image | Wrong publication type (reviews, abstracts) |
| 25 | Chen | 2012 | 3D/2D model-to-image registration for quantitative dietary assessment | Wrong publication type (reviews, abstracts) |
| 26 | Jia | 2012 | 3D localization of circular feature in 2D image and application to food volume estimation | Wrong publication type (reviews, abstracts) |
| 27 | Jia | 2012 | Imaged based estimation of food volume using circular referents in dietary assessment | No fully automated AI |
| 28 | Jia | 2012 | Imaged based estimation of food volume using circular referents in dietary assessment | Duplicate |
| 29 | Liu | 2012 | An Intelligent Food-Intake Monitoring System Using Wearable Sensors | Duplicate |
| 30 | Martin | 2012 | Validity of the remote food photography method (RFPM) for estimating energy and nutrient intake in near real-time | No fully automated AI |
| 31 | Rahman | 2012 | Food Volume Estimation in a Mobile Phone Based Dietary Assessment System | Wrong publication type (reviews, abstracts) |
| 32 | Villalobos | 2012 | An image procesing approach for calorie intake measurement | Wrong publication type (reviews, abstracts) |
| 33 | Xu | 2012 | Image enhancement and quality measures for dietary assessment using mobile devices | Wrong publication type (reviews, abstracts) |
| 34 | Yue | 2012 | Measurement of food volume based on single 2-D image without conventional camera calibration | Wrong publication type (reviews, abstracts) |
| 35 | Chen | 2013 | Model-based measurement of food portion size for image-based dietary assessment using 3D/2D registration | Duplicate |
| 36 | Dehais | 2013 | Food volume computation for self dietary assessment applications | Wrong publication type (reviews, abstracts) |
| 37 | Dibiano | 2013 | Food image analysis for measuring food intake in free living conditions | Duplicate |
| 38 | He | 2013 | Food image analysis: Segmentation, identification and weight estimation | Wrong publication type (reviews, abstracts) |
| 39 | Xu | 2013 | Image-based food volume estimation | Wrong publication type (reviews, abstracts) |
| 40 | Xu | 2013 | Model-based food volume estimation using 3D pose | Wrong publication type (reviews, abstracts) |
| 41 | Ahmad | 2014 | A mobile phone user interface for image-based dietary assessment | Wrong publication type (reviews, abstracts) |
| 42 | Anthimopoulos | 2014 | A Food Recognition System for Diabetic Patients Based on an Optimized Bag-of-Features Model | Only food recognition/classification |
| 43 | Jia | 2014 | Accuracy of food portion size estimation from digital pictures acquired by a chest-worn camera | Duplicate |
| 44 | Martin | 2014 | Measuring food intake with digital photography | Wrong publication type (reviews, abstracts) |
| 45 | Nguyen | 2014 | Food image classification using local appearance and global structural information | Only food recognition/classification |
| 46 | Oliveira | 2014 | A mobile, lightweight, poll-based food identification system | Only food recognition/classification |
| 47 | Pouladzadeh | 2014 | Using graph cut segmentation for food calorie measurement | Wrong publication type (reviews, abstracts) |
| 48 | Pustozerov | 2014 | A Remote Monitoring System for Diabetes Patients | No digital food images |
| 49 | Schap | 2014 | Merging dietary assessment with the adolescent lifestyle | Wrong publication type (reviews, abstracts) |
| 50 | Sharp | 2014 | Feasibility and validity of mobile phones to assess dietary intake | Wrong publication type (reviews, abstracts) |
| 51 | Sun | 2014 | eButton: A Wearable Computer for Health Monitoring and Personal Assistance | Wrong publication type (reviews, abstracts) |
| 52 | Zheng | 2014 | Spectroscopy-based food classification with extreme learning machine | No digital food images |
| 53 | Beijbom | 2015 | Menu-Match: Restaurant-Specific Food Logging from Images | Wrong publication type (reviews, abstracts) |
| 54 | Boulos | 2015 | Towards an "Internet of Food": Food Ontologies for the Internet of Things | Wrong publication type (reviews, abstracts) |
| 55 | Fang | 2015 | Single-View Food Portion Estimation Based on Geometric Models | Wrong publication type (reviews, abstracts) |
| 56 | Harray | 2015 | A Novel Dietary Assessment Method to Measure a Healthy and Sustainable Diet Using the Mobile Food Record: protocol and Methodology | Wrong publication type (reviews, abstracts) |
| 57 | Hassannejad | 2015 | Using Small Checkerboards as Size Reference: A Model-Based Approach | Wrong publication type (reviews, abstracts) |
| 58 | Kalantarian | 2015 | Audio-based detection and evaluation of eating behavior using the smartwatch platform | No digital food images |
| 59 | Kawano | 2015 | FoodCam: A real-time food recognition system on a smartphone | No human assessment or ground truth |
| 60 | Kong | 2015 | DietCam: Multi-view regular shape food recognition with a camera phone | Only food recognition/classification |
| 61 | McAllister | 2015 | Semi-automated system for predicting calories in photographs of meals | Wrong publication type (reviews, abstracts) |
| 62 | Myers | 2015 | Im2Calories: Towards an automated mobile vision food diary | Duplicate |
| 63 | Pouladzadeh | 2015 | A virtualization mechanism for real-time multimedia-assisted mobile food recognition application in cloud computing | No human assessment or ground truth |
| 64 | Pouladzadeh | 2015 | Cloud-based SVM for food categorization | No human assessment or ground truth |
| 65 | Probst | 2015 | Dietary assessment on a mobile phone using image processing and pattern recognition techniques: Algorithm design and system prototyping | Only food recognition/classification |
| 66 | Steele | 2015 | An Overview of the State of the Art of Automated Capture of Dietary Intake Information | Wrong publication type (reviews, abstracts) |
| 67 | Thongpull | 2015 | A design automation approach for task-specific intelligent multi-sensory systems - Lab-on-spoon in food applications | No digital food images |
| 68 | Zhang | 2015 | "Snap-n-Eat": Food Recognition and Nutrition Estimation on a Smartphone | No human assessment or ground truth |
| 69 | Zhang | 2015 | "Snap-n-Eat": Food Recognition and Nutrition Estimation on a Smartphone | Duplicate |
| 70 | Zhu | 2015 | Multiple Hypotheses Image Segmentation and Classification With Application to Dietary Assessment | Only food recognition/classification |
| 71 | Fang | 2016 | A comparison of food portion size estimation using geometric models and depth images | Wrong publication type (reviews, abstracts) |
| 72 | Farinella | 2016 | Retrieval and classification of food images | Only food recognition/classification |
| 73 | He | 2016 | DietCam: Multiview Food Recognition Using a Multikernel SVM | Only food recognition/classification |
| 74 | Hippocrate | 2016 | Food Weight Estimation using Smartphone and Cutlery | Wrong publication type (reviews, abstracts) |
| 75 | Tatsuma | 2016 | Food Image Recognition Using Covariance of Convolutional Layer Feature Maps | Only food recognition/classification |
| 76 | Zhang | 2016 | Multi-Task Learning for Food Identification and Analysis with Deep Convolutional Neural Networks | Only food recognition/classification |
| 77 | Allegra | 2017 | A Multimedia Database for Automatic Meal Assessment Systems | Duplicate |
| 78 | Allman-Farinelli | 2017 | Technology Interventions to Manage Food Intake: Where Are We Now? | Wrong publication type (reviews, abstracts) |
| 79 | Ashman | 2017 | Validation of a Smartphone Image-Based Dietary Assessment Method for Pregnant Women | No fully automated AI |
| 80 | Bally | 2017 | Carbohydrate Estimation Supported by the GoCARB System in Individuals With Type 1 Diabetes: A Randomized Prospective Pilot Study | Wrong publication type (reviews, abstracts) |
| 81 | Boushey | 2017 | New mobile methods for dietary assessment: Review of image-assisted and image-based dietary assessment methods | Wrong publication type (reviews, abstracts) |
| 82 | Boushey | 2017 | New mobile methods for dietary assessment: review of image-assisted and image-based dietary assessment methods | Duplicate |
| 83 | Boushey | 2017 | Reported Energy Intake Accuracy Compared to Doubly Labeled Water and Usability of the Mobile Food Record among Community Dwelling Adults | Duplicate |
| 84 | Christ | 2017 | Diabetes60 - inferring bread units from food images using fully convolutional neural networks | Wrong publication type (reviews, abstracts) |
| 85 | Ciocca | 2017 | Food Recognition: A New Dataset, Experiments, and Results | Only food recognition/classification |
| 86 | Eftimov | 2017 | StandFood: Standardization of Foods Using a Semi-Automatic System for Classifying and Describing Foods According to FoodEx2 | Only food recognition/classification |
| 87 | Hassannejad | 2017 | A New Approach to Image-Based Estimation of Food Volume | Duplicate |
| 88 | Hassannejad | 2017 | Automatic diet monitoring: a review of computer vision and wearable sensor-based methods | Wrong publication type (reviews, abstracts) |
| 89 | Mezgec | 2017 | NutriNet: A Deep Learning Food and Drink Image Recognition System for Dietary Assessment | Only food recognition/classification |
| 90 | Miranda | 2017 | Use of computer vision techniques for automatic food classification by size | Wrong publication type (reviews, abstracts) |
| 91 | Pandey | 2017 | FoodNet: Recognizing Foods Using Ensemble of Deep Networks | Only food recognition/classification |
| 92 | Pouladzadeh | 2017 | Mobile Multi-Food Recognition Using Deep Learning | No human assessment or ground truth |
| 93 | Vu | 2017 | Wearable Food Intake Monitoring Technologies: A Comprehensive Review | Wrong publication type (reviews, abstracts) |
| 94 | Zheng | 2017 | Food Image Recognition via Superpixel Based Low-Level and Mid-Level Distance Coding for Smart Home Applications | Only food recognition/classification |
| 95 | Beltran | 2018 | Reliability and validity of food portion size estimation from images using manual flexible digital virtual meshes | No fully automated AI |
| 96 | Ciocca | 2018 | CNN-based features for retrieval and classification of food images | Only food recognition/classification |
| 97 | Ege | 2018 | Image-based food calorie estimation using recipe information | Duplicate |
| 98 | Fang | 2018 | Single-View Food Portion Estimation: Learning Image-to-Energy Mappings Using Generative Adversarial Networks | Wrong publication type (reviews, abstracts) |
| 99 | Gao | 2018 | Food volume estimation for quantifying dietary intake with a wearable camera | Wrong publication type (reviews, abstracts) |
| 100 | Heravi | 2018 | An optimized convolutional neural network with bottleneck and spatial pyramid pooling layers for classification of foods | Only food recognition/classification |
| 101 | Herrera | 2018 | Narrative review of new methods for assessing food and energy intake | Wrong publication type (reviews, abstracts) |
| 102 | Horiguchi | 2018 | Personalized Classifier for Food Image Recognition | Only food recognition/classification |
| 103 | KorousicSeljak | 2018 | Identification of Requirements for Computer-Supported Matching of Food Consumption Data with Food Composition Data | No digital food images |
| 104 | Liu | 2018 | A New Deep Learning-Based Food Recognition System for Dietary Assessment on An Edge Computing Service Infrastructure | Only food recognition/classification |
| 105 | Lu | 2018 | A multi-task learning approach for meal assessment | Wrong publication type (reviews, abstracts) |
| 106 | McAllister | 2018 | Combining deep residual neural network features with supervised machine learning algorithms to classify diverse food image datasets | Only food recognition/classification |
| 107 | Ming | 2018 | Food Photo Recognition for Dietary Tracking: System and Experiment | Wrong publication type (reviews, abstracts) |
| 108 | Spruijt-Metz | 2018 | Advances and Controversies in Diet and Physical Activity Measurement in Youth | Wrong publication type (reviews, abstracts) |
| 109 | Suh | 2018 | Real-time Calorie Extraction and Cuisine Classification through Food-Image Recognition | No human assessment or ground truth |
| 110 | Sundaravadivel | 2018 | Smart-Log: A Deep-Learning Based Automated Nutrition Monitoring System in the IoT | No digital food images |
| 111 | Thamarai | 2018 | Calorie measurement: predicting the nutrient content of food using image analysis | No comparison of AI to human assessment or ground truth |
| 112 | Vasiloglou | 2018 | A Comparative study on carbohydrate estimation: GoCARB vs. dietitians | Duplicate |
| 113 | Wang | 2018 | Context based image analysis with application in dietary assessment and evaluation | Only food recognition/classification |
| 114 | Yang | 2018 | Image-based food portion size estimation using a smartphone without a fiducial marker | Duplicate |
| 115 | Yigit | 2018 | Comparison of convolutional neural network models for food image classification | Only food recognition/classification |
| 116 | Zheng | 2018 | Mid-level deep Food Part mining for food image recognition | Only food recognition/classification |
| 117 | Aguilar | 2019 | Regularized uncertainty-based multi-task learning model for food analysis | Only food recognition/classification |
| 118 | Ahn | 2019 | Estimating the Composition of Food Nutrients from Hyperspectral Signals Based on Deep Neural Networks | No digital food images |
| 119 | Allegra | 2019 | Learning to rank food images | Duplicate |
| 120 | Fang | 2019 | An end-to-end image-based automatic food energy estimation technique based on learned energy distribution images: Protocol and methodology | Duplicate |
| 121 | Jia | 2019 | Automatic food detection in egocentric images using artificial intelligence technology | Only food recognition/classification |
| 122 | KakaBra | 2019 | A smart nutrition management system and nutrition related diseases in humans | No digital food images |
| 123 | Khan | 2019 | Food items detection and recognition via multiple deep models | Only food recognition/classification |
| 124 | Lo | 2019 | A Novel Vision-based Approach for Dietary Assessment using Deep Learning View Synthesis | Wrong publication type (reviews, abstracts) |
| 125 | Lu | 2019 | An artificial intelligence-based system for nutrient intake assessment of hospitalised patients | Duplicate |
| 126 | Mezgec | 2019 | Mixed deep learning and natural language processing method for fake-food image recognition and standardization to help automated dietary assessment | Only food recognition/classification |
| 127 | Pan | 2019 | Image Augmentation-Based Food Recognition with Convolutional Neural Networks | Only food recognition/classification |
| 128 | Park | 2019 | The development of food image detection and recognition model of Korean food for mobile dietary management | Only food recognition/classification |
| 129 | Subhi | 2019 | Vision-Based Approaches for Automatic Food Recognition and Dietary Assessment: A Survey | Wrong publication type (reviews, abstracts) |
| 130 | Teng | 2019 | Recognition of Chinese food using convolutional neural network | Only food recognition/classification |
| 131 | Yu | 2019 | Personalized Food Image Classifier Considering Time-Dependent and Item-Dependent Food Distribution | Only food recognition/classification |
| 132 | Yunus | 2019 | A Framework to Estimate the Nutritional Value of Food in Real Time Using Deep Learning Techniques | No human assessment or ground truth |
| 133 | Zhou | 2019 | Application of Deep Learning in Food: A Review | Wrong publication type (reviews, abstracts) |
| 134 | Aguilar | 2020 | Uncertainty-aware integration of local and flat classifiers for food recognition | Only food recognition/classification |
| 135 | Alfonsi | 2020 | Carbohydrate Counting App Using Image Recognition for Youth With Type 1 Diabetes: pilot Randomized Control Trial | No comparison of AI to human assessment or ground truth |
| 136 | Allegra | 2020 | A review on food recognition technology for health applications | Wrong publication type (reviews, abstracts) |
| 137 | Aslan | 2020 | Benchmarking algorithms for food localization and semantic segmentation | Only food recognition/classification |
| 138 | Burkapalli | 2020 | Food image segmentation using edge adaptive based deep-CNNs | Only food recognition/classification |
| 139 | Choi | 2020 | ISPY: a pilot study of a novel carbohydrate counting smartphone app for youth with type 1 diabetes | Wrong publication type (reviews, abstracts) |
| 140 | Chui | 2020 | Validating an automated image identification process of a passive image-assisted dietary assessment method: proof of concept | Only food recognition/classification |
| 141 | Ciocca | 2020 | State Recognition of Food Images Using Deep Features | Only food recognition/classification |
| 142 | Furtado | 2020 | Human Visual System vs Convolution Neural Networks in food recognition task: An empirical comparison | Only food recognition/classification |
| 143 | Ho | 2020 | Validity of image-based dietary assessment methods: A systematic review and meta-analysis | Wrong publication type (reviews, abstracts) |
| 144 | Ji | 2020 | Validity and Usability of a Smartphone Image-Based Dietary Assessment App Compared to 3-Day Food Diaries in Assessing Dietary Intake Among Canadian Adults: randomized Controlled Trial | No fully automated AI |
| 145 | Jiang | 2020 | DeepFood: Food Image Analysis and Dietary Assessment via Deep Model | No human assessment or ground truth |
| 146 | Jiang | 2020 | Few-shot Food Recognition via Multi-view Representation Learning | Only food recognition/classification |
| 147 | Jiang | 2020 | Multi-Scale Multi-View Deep Feature Aggregation for Food Recognition | Only food recognition/classification |
| 148 | Jobarteh | 2020 | Development and validation of an objective, passive dietary assessment method for estimating food and nutrient intake in households in lowand middle-income countries: A study protocol | Wrong publication type (reviews, abstracts) |
| 149 | Kavitha | 2020 | Estimation of Calories and Micro Nutrients from Food Image Using Deep Learning Algorithm | No comparison of AI to human assessment or ground truth |
| 150 | Knez | 2020 | Food object recognition using a mobile device: Evaluation of currently implemented systems | Wrong publication type (reviews, abstracts) |
| 151 | Lo | 2020 | Image-Based Food Classification and Volume Estimation for Dietary Assessment: A Review | Wrong publication type (reviews, abstracts) |
| 152 | Lu | 2020 | goFOOD(TM): An Artificial Intelligence System for Dietary Assessment | Duplicate |
| 153 | Lu | 2020 | goFOOD(TM): An artificial intelligence system for dietary assessment | Duplicate |
| 154 | Minija | 2020 | Food recognition using neural network classifier and multiple hypotheses image segmentation | No comparison of AI to human assessment or ground truth |
| 155 | Ossani | 2020 | Machine learning in classification and identification of nonconventional vegetables | Only food recognition/classification |
| 156 | Pan | 2020 | A combinational convolutional neural network of double subnets for food-ingredient recognition | Only food recognition/classification |
| 157 | Pan | 2020 | A Novel Combinational Convolutional Neural Network for Automatic Food-Ingredient Classification | Only food recognition/classification |
| 158 | Rachakonda | 2020 | iLog: An Intelligent Device for Automatic Food Intake Monitoring and Stress Detection in the IoMT | No human assessment or ground truth |
| 159 | Song | 2020 | Hybrid Attention-Based Prototypical Network for Unfamiliar Restaurant Food Image Few-Shot Recognition | Only food recognition/classification |
| 160 | Sowah | 2020 | Design and Development of Diabetes Management System Using Machine Learning | No human assessment or ground truth |
| 161 | Tahir | 2020 | An Open-Ended Continual Learning for Food Recognition Using Class Incremental Extreme Learning Machines | Only food recognition/classification |
| 162 | Tan | 2020 | Quantized Deep Residual Convolutional Neural Network for Image-Based Dietary Assessment | Only food recognition/classification |
| 163 | Tasci | 2020 | Voting combinations-based ensemble of fine-tuned convolutional neural networks for food image recognition | Only food recognition/classification |
| 164 | Wibisono | 2020 | Traditional food knowledge of Indonesia: a new high-quality food dataset and automatic recognition system | Only food recognition/classification |
| 165 | Won | 2020 | Multi-Scale CNN for Fine-Grained Image Recognition | Only food recognition/classification |
| 166 | Yang | 2020 | A Hierarchical deep model for food classification from photographs | Only food recognition/classification |
| 167 | Yoshimura | 2020 | Application of Mass Spectrometry Imaging for Visualizing Food Components | No digital food images |
| 168 | Zhang | 2020 | Wi-HSNN: A subnetwork-based encoding structure for dimension reduction and food classification via harnessing multi-CNN model high-level features | Only food recognition/classification |
| 169 | Zhao | 2020 | JDNet: A Joint-Learning Distilled Network for Mobile Visual Food Recognition | Only food recognition/classification |
| 170 | Alfonsi | 2021 | Ispy: novel carbohydrate counting smartphone app for youth with type 1 diabetes | Wrong publication type (reviews, abstracts) |
| 171 | Alshomrani | 2021 | Food Detection by Fine-Tuning Pre-trained Convolutional Neural Network Using Noisy Labels | Only food recognition/classification |
| 172 | Chen | 2021 | A Study of Multi-Task and Region-Wise Deep Learning for Food Ingredient Recognition | Only food recognition/classification |
| 173 | Chen | 2021 | An Exploratory Approach to Deriving Nutrition Information of Restaurant Food from Crowdsourced Food Images: Case of Hartford | No results reported |
| 174 | Chopra | 2021 | Recent Studies on Segmentation Techniques for Food Recognition: A Survey | Wrong publication type (reviews, abstracts) |
| 175 | Fakhrou | 2021 | Smartphone-based food recognition system using multiple deep CNN models | Only food recognition/classification |
| 176 | Feng | 2021 | Application of Visible/Infrared Spectroscopy and Hyperspectral Imaging With Machine Learning Techniques for Identifying Food Varieties and Geographical Origins | Wrong publication type (reviews, abstracts) |
| 177 | Jeyalakshmi | 2021 | PRESCRIPTIVE ANALYTICS OF CONSTRAINT OPTIMISATION OF DIABETES DIET EXHORTATION BY USING INFORMATION SYSTEMS | Not fully automated |
| 178 | Jia | 2021 | Estimating Dining Plate Size From an Egocentric Image Sequence Without a Fiducial Marker | No digital food images |
| 179 | Liang | 2021 | MVANet: Multi-Task Guided Multi-View Attention Network for Chinese Food Recognition | Only food recognition/classification |
| 180 | Liu | 2021 | Food and Ingredient Joint Learning for Fine-Grained Recognition | Only food recognition/classification |
| 181 | Lohala | 2021 | A novel deep learning neural network for fast-food image classification and prediction using modified loss function | Only food recognition/classification |
| 182 | Mezgec | 2021 | Deep Neural Networks for Image-Based Dietary Assessment | Only food recognition/classification |
| 183 | Qiu | 2021 | Counting Bites and Recognizing Consumed Food from Videos for Passive Dietary Monitoring | No digital food images |
| 184 | Ramesh | 2021 | Food Detection and Segmentation from Egocentric Camera Images | Wrong publication type (reviews, abstracts) |
| 185 | Sak | 2021 | Artificial intelligence in nutrients science research: A review | Wrong publication type (reviews, abstracts) |
| 186 | Siemon | 2021 | Sequential transfer learning based on hierarchical clustering for improved performance in deep learning based food segmentation | Only food recognition/classification |
| 187 | Stankoski | 2021 | Smartwatch-Based Eating Detection: Data Selection for Machine Learning from Imbalanced Data with Imperfect Labels | No digital food images |
| 188 | Tahir | 2021 | A Comprehensive Survey of Image-Based Food Recognition and Volume Estimation Methods for Dietary Assessment | Wrong publication type (reviews, abstracts) |
| 189 | Tahir | 2021 | Explainable deep learning ensemble for food image analysis on edge devices | Only food recognition/classification |
| 190 | Tan | 2021 | Neural Architecture Search for Lightweight Neural Network in Food Recognition | Only food recognition/classification |
| 191 | Wang | 2021 | Food Image Recognition and Food Safety Detection Method Based on Deep Learning | Only food recognition/classification |
| 192 | Wu | 2021 | A Framework of Visual Checkout System Using Convolutional Neural Networks for Bento Buffet | No comparison of AI to human assessment or ground truth |
| 193 | Xiao | 2021 | A Simplified CNNs Visual Perception Learning Network Algorithm for Foods Recognition | Only food recognition/classification |
| 194 | Yarlagadda | 2021 | Saliency-Aware Class-Agnostic Food Image Segmentation | Only food recognition/classification |
| 195 | Aguilar | 2022 | Uncertainty-aware selecting for an ensemble of deep food recognition models | Only food recognition/classification |
| 196 | Chew | 2022 | Mobile Food Journalling Application with Convolutional Neural Network and Transfer Learning: A Case for Diabetes Management in Malaysia | Only food recognition/classification |
| 197 | Dalakleidi | 2022 | Applying Image-Based Food-Recognition Systems on Dietary Assessment: A Systematic Review | Wrong publication type (reviews, abstracts) |
| 198 | Hafiz | 2022 | Image-based soft drink type classification and dietary assessment system using deep convolutional neural network with transfer learning | No results reported |
| 199 | Ittisoponpisan | 2022 | Pushing the Accuracy of Thai Food Image Classification with Transfer Learning | Only food recognition/classification |
| 200 | Jain | 2022 | Automated Identification Algorithm Using CNN for Computer Vision in Smart Refrigerators | Only food recognition/classification |
| 201 | Jia | 2022 | A Novel Approach to Dining Bowl Reconstruction for Image-Based Food Volume Estimation | Not fully automated |
| 202 | Khan | 2022 | Automatic Prediction of Glycemic Index Category from Food Images Using Machine Learning Approaches | Only food recognition/classification |
| 203 | Liu | 2022 | Deep-Learning-Assisted Multi-Dish Food Recognition Application for Dietary Intake Reporting | Only food recognition/classification |
| 204 | Lubura | 2022 | Food Recognition and Food Waste Estimation Using Convolutional Neural Network | No human assessment or ground truth |
| 205 | Nguyen | 2022 | Relative validity of a mobile AI-technology-assisted dietary assessment in adolescent females in Vietnam | Not fully automated |
| 206 | Nguyen | 2022 | SibNet: Food instance counting and segmentation | Only food recognition/classification |
| 207 | Oduru | 2022 | Healthy vs. Unhealthy Food Images: Image Classification of Twitter Images | Only food recognition/classification |
| 208 | Raju | 2022 | FOODCAM: A Novel Structured Light-Stereo Imaging System for Food Portion Size Estimation | No artificial intelligence |
| 209 | Shen | 2022 | Development of a new machine vision algorithm to estimate potato's shape and size based on support vector machine | Only food recognition/classification |
| 210 | Sheng | 2022 | Food recognition via an efficient neural network with transformer grouping | Only food recognition/classification |
| 211 | Smith | 2022 | Food Volume Estimation by Integrating 3D Image Projection and Manual Wire Mesh Transformations | Not fully automated |
| 212 | Van Wymelbeke-Delannoy | 2022 | A Cross-Sectional Reproducibility Study of a Standard Camera Sensor Using Artificial Intelligence to Assess Food Items: The FoodIntech Project | Only food recognition/classification |
| 213 | Vasiloglou | 2022 | Multimedia Data-Based Mobile Applications for Dietary Assessment | Wrong publication type (reviews, abstracts) |
| 214 | Viveka | 2022 | To Control Diabetes Using Machine Learning Algorithm and Calorie Measurement Technique | Not fully automated |
| 215 | Wang | 2022 | A review on vision-based analysis for automatic dietary assessment | Wrong publication type (reviews, abstracts) |
| 216 | Joshua | 2023 | Health to Eat: A Smart Plate with Food Recognition, Classification, and Weight Measurement for Type-2 Diabetic Mellitus Patients' Nutrition Control | Not fully automated |
| 217 | Kaur | 2023 | Deep neural network for food image classification and nutrient identification: A systematic review | Wrong publication type (reviews, abstracts) |
| 218 | Mortazavi | 2023 | A Review of Digital Innovations for Diet Monitoring and Precision Nutrition | Wrong publication type (reviews, abstracts) |
| 219 | Oskouei | 2023 | FoodRecNet: a comprehensively personalized food recommender system using deep neural networks | Not fully automated |
| 220 | Ramirez-Contreras | 2023 | Relative Validity and Reliability of the Remind App as an Image-Based Method to Assess Dietary Intake and Meal Timing in Young Adults | Not fully automated |

**eTable 4.** Results and key findings for included articles

| **Author (year)** | **Ground truth definition** | **Description of comparison to human assessors or other** | **Type of accuracy results reported** **^a^** | **Results for area, volume, weight, mass, and energy/nutrient estimations** | **Key findings for accuracy of the proposed approaches** |
| --- | --- | --- | --- | --- | --- |
| Zhu, et al. (2010) | For volume estimation: water displacement method, radius method  For energy/nutrient estimation: manually traced foods in image and generated mask images; reports correct nutrient information was available, but source not reported | Study staff and other “individuals” traced the contour of food items, generated a mask image and corresponding food label. Results “were shown to graduate students in the Department of Foods and Nutrition at Purdue University for evaluation” (p. 10) | Percentage error | **Percentage error rate for estimated area and volumes:**  Area (n=2 food items): mean = 0.79% (SD = 0.65%)  Volume by water displacement (n=7 food items): mean = 5.68% (7.19%)  Volume by measured radius (n=7 food items): mean = 5.65% (SD = 5.25%)  **Percentage error rate for estimated mass:**  Garlic bread (a) = 3.4%  Garlic bread (b) = 56.4%  Yellow cake (a) = 36.6%  Yellow cake (b) = 7.0%  **Percentage error for energy intake for different amounts of training data in the automatic method:**  [Mean across 78 participants]  10% training data = within 10% margin of ground truth  25% training data = within 3% margin of ground truth  50% training data = within 1% margin of ground truth | Error rates for estimated volumes were 5.7% compared to both water displacement and measured radius methods. Estimated energy intake improved to within 1% of correct nutrient information when 50% training data was used in the automatic method. |
| Kong and Tan (2012) | For volume estimation: the average of the value from water displacement and that from PhotoModeler | None | Measured and estimated food volumes; No results presented for calories | **Measured and estimated food volumes (cm^3^):**  [Mean measured across 10 samples / mean estimated with model (average absolute deviation for estimate); calculated mean relative error based on reported measured and estimated means]  Apple = 310.5/286.7 (23.8); 7.7%  Orange = 207/198.4 (8.6); 4.2%  Pear = 221/194.2 (26.8); 12.1%  Banana = 215.8/204.1 (11.7); 5.4%  Burger = 678.2/623 (55.2); 8.1%  Sub = 1280.1/1211.7 (68.4); 5.3% | The maximum standard deviation of volume estimation was ±20% error suggesting that calories could be roughly estimated based on volume estimation plus average calorie density. Calculated mean relative errors were ≤12.1% for all foods. |
| Lee, et al. (2012) | For weight estimation:  All foods and beverages were pre-weighed separately to one-tenth of a gram prior to plating; calculated the average weight of the food and beverages served during meals and what was actually eaten | 15 adolescent participants assessed each food item portion using multiple measurement descriptors (MDes) from the What In The Foods You Eat Search Tool, two-dimensional (2D) images of standard-sized portions, and/or other method | Ratio of estimates (automated and self-reports) to actual weights | **Ratio of estimate to known mean weights (g) and energy (kcal):**  [Based on means from 15 adolescents]  Ratios for 19 listed foods ranged from 0.89 (sausage links; spaghetti with sauce, cheese) to 4.61 (lettuce [salad])  Most accurate ratios were 1.01 (strawberry jam), 1.04 (orange juice), and 0.95 (2% milk; cheeseburger)  **Energy (kcal):**  [Based on meals served to 15 adolescents]  Mean energy served (ground truth) = 2723 ± 51  Mean energy estimated from automatic volume computations = 3588 ± 180 | Across 19 foods, the mean ratio of automated weight estimate to known weight ranged from 0.89 to 4.61 with 9 foods between 0.80 to 1.20. Lettuce produced the largest error, and strawberry jam the smallest error. |
| Chen, et al. (2013) | For volume estimation: Manually or water displacement | None | Relative error | **Relative error (%) of food volume estimation:**  Mean across 17 foods = 3.69  [Mean (SD) for 1 food item on 4 plates with different radius and depth values]  Plate 1 = 2.05 (1.69)  Plate 2 = 0.53 (-5.77)  Plate 3 = 1.16 (1.51)  Plate 4 = -0.20 (0.58) | Using a single-view 2D image, this framework estimated food volume with an average error of 3.69%. |
| Pouladzadeh, et al. (2014) | For food portion estimation:  measured the area of each food portion twice: once by hand from the image, and once using proposed method  In the second scenario, the real food portion is actually weighted and its calorie is extracted using tables. | None | Error; absolute accuracy; uncertainty of measurement | **Error (%) for single food area measured by proposed method vs. by hand from image:**  Bread = 0.63  Cake = 2.30  Spaghetti = -3.07  Cookies = 0.50  Omelet = 10.5  **Absolute accuracy (%) of calculated vs. real calories:**  Average across 8 single foods = 86  **Repeated uncertainty of measurement for calories measured by App** **across 3 locations, 3 angles, and 3 cameras compared to real calories:**  [Calculated relative errors averaged across locations, angles and cameras]  Across 11 single foods, range was 0.21 for orange to 20.73 for tomato | This method measured area with an average error of 3.4% and had an absolute accuracy of 86% for calorie estimation. |
| Siswantoro, et al. (2014) | For volume estimation: Water displacement | None | Absolute relative error (ARE) and coefficient of variation (CV); correlation coefficient; paired t-test; mean volume difference | **Volume (cc) measurement by proposed method vs. water displacement method:**  [Mean ARE % (CV %)]  Ball (n=2) = 0.02 (0.10)  Apple (n=50) = 1.00 (0.18)  Mango (n=50) = 0.97 (0.16)  Tomato (n=50) = 0.82 (0.18)  **Volume measurements (cc) from proposed method vs. water displacement compared by paired t-tests and tests of correlations:**  Apple: mean diff (SD) in cc = -0.04 (1.64); 95% CI -0.50, 0.43; *P* = 0.87; *r* = 0.988  Mango: mean diff (SD) in cc = 0.70 (3.52); 95% CI -0.30, 1.70; *P* = 0.17; *r* = 0.996  Tomato: mean diff (SD) in cc = -0.11 (1.24); 95% CI -0.47, 0.24; *P* = 0.52; *r* = 0.998 | Compared to the water displacement method, the proposed method estimated volume with an absolute relative error <3% for over 98% of samples and a coefficient of variation <0.5% for 98% of samples. Volume estimations by water displacement and the proposed method were highly correlated (*r*≥0.988) and showed no significant differences (*P*>0.1) for all samples. |
| Anthimopoulos, et al. (2015) | 3D shapes: a depth sensor, the Xtion PRO LIVE by Asus was used to scan the dishes. Food types and locations were manually specified on the images.  CHO estimation: table of CHO to volume densities for different food types was generated based on the USDA Food and Nutrient Database for Dietary Studies.  The CHO densities for each food class were averaged to determine the overall CHO density. Together with the generated nutritional table these data yielded the real CHO content of each of the food items | None | Absolute error; absolute percentage error | **Absolute error (g) and absolute percentage error (%) for carbohydrate estimation of served meals:**  [Means across 24 dishes with 12 estimates each]  Mean (SD) absolute error (g) = 6 (8)  Mean (SD) absolute percentage error (%) = 10 (13)  Mean (SD) percentage error range (%) = 3 (2) for dish #9 to 16 (10) for dish #23 | The mean absolute percentage error in carbohydrate estimation for normal-sized dishes was 10 ± 13%. |
| Huang, et al. (2015) | For volume estimation: water displacement | None | Error rate | **Error rate (%) for estimated vs. actual volume (ml) and carbohydrate content (g):**  [Average % (range) across 6 food items]  Volume = 6.86 (4.43 for peach to 13.9 for apple #2)  Carbohydrates = 8.18 (2.45 for peach to 16.1 for apple #2) | Using limited datasets, the study demonstrated a mean error rate of 6.9% for volume estimation and 8.2% for carbohydrate estimation. |
| Rhyner, et al. (2016)^b^ | For weight: household scale  Carbohydrate content: using the USDA National Nutrient Database for Standard Reference. | 19 participants with type 1 diabetes mellitus who were mobile phone users (only 1 was not) made estimations of the carbohydrate content of meals of different sizes (small, medium, or large) with or without using the GoCARB application. Prior to using the app, participants received a short training and detailed written user manual. | Absolute error; absolute percentage error | **Absolute error (g) for carbohydrate estimation by participants or GoCARB:**  [mean (SD) across 19 participants with or without diabetes]  Participants = 27.89 (38.20)  GoCARB = 12.28 (9.56)  Difference in estimation errors, *P*=.001 (after removing a participant with extreme errors, *P*=.01)  **Absolute percentage error (%) for carbohydrate estimation by participants or GoCARB:**  [mean (SD) across 19 participants with or without diabetes]  Participants = 54.8 (72.3)  GoCARB = 26.2 (18.7)  **Carbohydrate (g) counting errors by participants or GoCARB for different meal sizes:**  [Participant mean (SD)]  Small = 18.47 (28.86)  Medium = 26.39 (38.20)  Large = 38.82 (47.03)  [GoCARB mean (SD)]  Small = 10.24 (6.16)  Medium = 10.12 (8.02)  Large = 16.38 (12.30) | The GoCARB system performed significantly better (*P*=.001) at carbohydrate estimation (mean absolute error=12.28 g, SD=9.56 g) than human participants (mean absolute error=27.89 g, SD=38.20 g). Carbohydrate counting errors increased with meal size for both participants and GoCARB. |
| Dehais, et al. (2017) | For volume estimation:  The dominant horizontal plane (table plane) was extracted with RANSAC, shifted to the true dish height, after which the ground truth volume was extracted. | None | Mean absolute percentage error (MAPE) in volume for different 3D mesh sizes, different relative angles with a fixed first image, different plates, and different datasets overall; signed percentage error; coefficient of variation (CV_i_) of estimates | **MAPE overall (%) for volume estimations:**  [Different 3D mesh sizes (2^10^ to 2^14^)]  Range = 8.2 for 2^12^ to 9.9 for 2^10^  [Different relative angles (5º to 35º) with a fixed first image (*Angles-13* dataset)]  Range = 5.1 at 15º and 20º to 30.4 at 35º  [Different plates (*Plates-18* dataset)]  Dinner = 8.9  Soup = 6.4  Oval = 7.1  [Different datasets]  Meals-45 = 8.2  Meals-14 = 9.8  Plates-18 = 7.4  **Signed percentage error (%) for volume estimates per item:**  [113 different food items and 24 estimates (2712 values) from *Meals-45* dataset]  Smooth and symmetrical distribution with peak and mean around 0, 69% of samples within ± 10, and 95% within ± 20  **CV_i_ (%) of volume estimates per item:**  [113 different food items from *Meals-45* dataset]  Average = 7.1  Distribution with 81.8% of values under 10 and 99.2% under 20 | The fully automated dietary system estimated volume with an average error of <10% (in 5.5 seconds per dish) for 77 real dishes of known volume. |
| Hassannejad, et al. (2017) | Volume estimation: For part 1 (solid foods) used an industrial 3D laser scanner Sick LMS400 (SICK AG, Waldkirch, Germany) to acquire a 3D model  For part 2 (non-solid foods) assessed using a scale | None | Error rate | **Error rate (%) in volume estimations:**  [Average (range)]  Solid shapes (n=6) = 8.27 (4.9 to 19.1)  Non-solid shapes (n=4) = 6.7 (5.1 to 7.1) | The image-based modeling approach achieved an average accuracy of 92% and an average processing time of ~23 s on a test set with images of different pastas and breads. |
| Minija and Emmanuel (2017) | For volume estimation: "a set of measurements which contain the more accurate value of the object." | None | Actual and estimated calorie values; mean square error (MSE) | **Errors for calorie estimation by the proposed method:**  [Calculated average absolute (relative) errors]  Image 1 (n=3 segments) = 0 (0.0%)  Image 2 (n=3 segments) = 0 (0.0%)  Image 3 (n=3 segments) = 0 (0.0%)  Image 4 (n=3 segments) = 5 (14.3%)  **MSE for calorie estimation by the proposed method:**  Image 1 = 0  Image 2 = 0  Image 3 = 0  Image 4 = 75 | The proposed method, a feed-forward NN classifier, was able to accurately estimate (0% error) calorie values for food segments across 4 images 11/12 times resulting in an MSE of 0 for images 1-3, and an MSE of 75 for image 4. |
| Todd, et al. (2017) | For portion served and consumed: Dietitians’ digital observations | 3 assessors (Dietitians from Cornell Universities Division of Nutritional Sciences; raters needed to be a registered dietitian with no prior experience with DFIA); after training, raters viewed pre- and post-lunch photographs and estimated portion served and percentage consumed using pre-and post-lunch photos on a computer screen and a hardcopy reference guide of standard serving sizes and corresponding weights of fruits and vegetables based on the USDA National Nutrient Database for Standard Reference;  Each dietitian assessed the same 41 lunch tray image pairs, 3 of which were shown twice to assess inter-rater reliability. | Mean differences; Spearman’s and Pearson’s correlation coefficients; Bland Altman differences | **Estimates by Digital Food Image Analysis (DFIA) vs. dieticians for fruits and vegetables served or consumed:**  [Mean differences (SE) and *P*-values from paired t-tests for 222 fruit and vegetable items; Spearman’s correlations (*r_s_*; all *P*<0.001); Pearson’s correlations (*r_p_*; all *P*<0.01)]  Fruits served (g) = -1.8 (4.6), *P*=0.698; *r_s_*=0.339; *r_p_*=0.281  Vegetables served (g) = -10.5 (5.7), *P*=0.070; *r_s_*=0.442; *r_p_*=0.287  Fruits and vegetables served (g) = -6.0 (3.7), *P*=0.101; *r_s_*=0.500; *r_p_*=0.422  Fruits consumed (%) = 0.5 (1.8), *P*=0.794; *r_s_*=0.890; *r_p_*=0.906  Vegetables consumed (%) = -1.4 (3.6), *P*=0.701; *r_s_*=0.669; *r_p_*=0.627  Fruits and vegetables consumed (%) = -0.4 (2.0), *P*= 0.825; *r_s_*=0.791; *r_p_*=0.779  [Mean difference (SD) from Bland and Altman plots; upper limit, lower limit]  Fruit and vegetable servings (g) = -6.7 (45.5); -95.5, 82.0  Fruit and vegetable consumption (%) = -0.4 (27.9); -55.1, 54.3 | Results of paired t tests showed no significant differences between DFIA and digital observation by dietitians despite some serving size underestimation in the DFIA-derived estimates. Although correlations were moderate, results showed large standard deviations due to specific items like diced fruit. As serving sizes increased, so did DFIA overestimations. DFIA estimates were less variable when <40% or >60% of foods were consumed. |
| Ege and Yanai (2018) | For calories/energy estimation: commercial cooking recipe sites provide recipes annotated with calorie values. | None | Relative error; absolute error; correlation coefficient; ratio of estimated value within the relative error of 20% and 40% | **Food calorie estimation with various combinations of auxiliary information (calories only [single-task CNN], or calories plus categories, ingredients, and/or directions [multi-task CNN]):**  [Ranges for performance on the Japanese recipe dataset 4.1]  Relative error (%) = 27.3 (calories, directions, and categories) to 29.4 (calories only)  Absolute error (kcal) = 91.2 (calories, categories, ingredients, and directions) to 100.7 (calories only)  Correlation = 0.778 (calories only) to 0.817 (calories, categories, ingredients, and directions)  Ratio (%) of estimated value within the relative error of 20% = 45.9 (calories only) to 50.1 (calories, categories, ingredients, and directions)  [Ranges for performance on the American recipe dataset]  Relative error (%) = 41.7 (calories, categories, and ingredients) to 43.3 (calories only)  Absolute error (kcal) = 119.6 (calories, categories, and ingredients) to 128.5 (calories only)  Correlation = 0.293 (calories only) to 0.383 (calories, categories, and ingredients)  Ratio (%) of estimated value within the relative error of 20% = 32.2 (calories only) to 35.1 (calories, categories, and ingredients) | Estimating food calorie from a food photo by simultaneous learning of food calories, categories, ingredients and cooking directions using multi-task CNNs consistently produced better results than independent single-task CNNs. Compared to image-search based calorie estimation using VGG16 pre-trained with the ImageNet 1000-class dataset, the multi-task CNN reduced relative error about 20% and absolute error 19 kcal. |
| Emmanuel and Minija (2018) | For calories/energy estimation: database NR; presumably, GT was taken from UNIMIB2016 dataset; calorie value of each segmented food item is found based on area of pixels | None | Mean square error (MSE) for calorie estimation | **MSE for calorie estimation by the proposed model:**  [Comparative analysis for the proposed model (CSW-WLIFC with WLM-NN) vs. 3 other models: WLIFC with LM-NN, CSW-WLIFC with LM-NN, and WLIFC with WLM-NN]  Food category 1 = 0.14 (comparator range = 0.22 to 0.43)  Food category 8 = 0.02 (comparator range = 0.03 to 0.57) | Compared to existing models, the proposed model showed improved performance based on a lower MSE for calorie estimation using food images from the UNIMIB2016 dataset. |
| Lo, et al. (2018) | For volume estimation: based on the given object dimensions  For the objects with a general shape (geometric calculations)  For irregular objects, the mean estimated volume Vg as another reference volume to evaluate the performance of the proposed algorithms for objects without ground truth volume as prior knowledge | None | Error of estimated volumes | **Error (%) of the estimated volume using point cloud completion:**  Orange = 1.7  Cube = 1.0  Tuna fish can = 2.2  Pudding box = 4.8  **Error (%) of the estimated volume using the iterative closest point algorithm:**  Range across 8 food object items was 3.3 (banana and potted meat can) to 9.4 (pudding box) | The proposed integrated approach based on the depth-sensing technique and deep learning view synthesis achieved an accuracy in volume estimation of up to 93%. |
| Subhi, et al. (2018) | For weight estimation: weighing scale | None | Uncertainty (relative error) | **Relative error (%) for actual measurements and estimated values:**  [Volume]  Burger bun = 2.4  Fried chicken thigh = 12  Slice of pizza = 8.6  Brownie = 13.3  [Weight]  Burger bun = 8.4  Fried chicken thigh = 9.1  Slice of pizza = 10  Brownie = 13.3  [Calories]  Bread, whole-wheat, prepared from recipe = 2.0  12” veggie pizza slice = 9.6  KFC deep fried chicken breast = 9.0  Chocolate cake and walnut brownies = 13.4 | The proposed algorithm applied to images obtained from wearable smart glasses with two image sensors (requiring no referent) showed measurement uncertainty ≤ ~13% for automatically estimated volumes, weights, and calories. |
| Vasiloglou, et al. (2018)^b^ | For weight estimation: weighed using household scales (Kenwood, model AT850B) and ground truth (GTR) was estimated using the meal’s exact food items, as it appears in the USDA National Nutrient Database for Standard Reference | 6 assessors (dieticians with a BSc degree in Nutrition and Dietetics and who were German speaking with at least five years experience with diabetic patients and in CHO counting as professional) using the same 222 images; to estimate CHO content, dietitians could use the Diabetic Exchange List from a committee of the American Diabetes Association and the American Dietetic Association, but the final decision to use the proposed list was made by the dietitians. They were blinded to the actual weight of the meals, their CHO content, and the corresponding food items. | Absolute error; correlations | **Absolute error (g) for carbohydrate estimation by dieticians or GoCARB system:**  [mean (SD) across 54 meals]  Dieticians = 14.9 (10.12)  GoCARB = 14.8 (9.73)  Difference in estimation errors, *P* = 0.93  **Correlations for carbohydrate estimation accuracy by dieticians or GoCARB:**  Dieticians, *r* = 0.89, *P* < 0.001  GoCARB, *r* = 0.76, *P* < 0.001  **Absolute error (g) for carbohydrate estimation for different meal sizes:**  [Dieticians’ mean (SD) across 16 meals per size]  Small = 5.9 (3.5)  Medium = 7.6 (6.3)  Large = 19.4 (15.2)  [GoCARB’s mean (SD) across 16 meals per size]  Small = 8.5 (5.6)  Medium = 11.3 (8.9)  Large = 20.7 (11.6)  [Difference in absolute errors for dieticians vs. GoCARB]  Small, *P* = 0.18  Medium, *P* = 0.27  Large, *P* = 0.41 | Dietitians’ visual estimations and GoCARB estimations of carbohydrates were not significantly different from each other (*P* = 0.93). Both estimates correlated highly with ground truth based on the weighing method (*r* > 0.75, *P* < 0.001), and both estimations of carbohydrates had a mean absolute error <15 g. |
| Yang, et al. (2018) | For volume estimation:  Experiment 1: used Food Replica with known volumes  Experiment 2: cup measures (for liquids and grains), by water displacement (for submergible ones) or with a ruler (for cuboids) | None | Absolute error; root mean-square error (RMSE); differences in relative error | **Absolute error (%) for volume estimation by models with and without training:**  [Average across 15 different food items]  Without training = 105.80  With training = 26.97  **RMSE (%) for volume estimation by models with and without training:**  [Average across 15 different food items]  Without training = 147.84  With training = 42.39  **Differences in relative error of food portion size estimation tested with and without training:**  For 13 food items, *P*<0.01  For 1 item (broccoli), *P*<0.05  For 1 item (egg), *P*>0.05 | This fiducial-marker-free image-based food portion size estimation using a trained model had an average absolute error of 17% for large-volume food items (n=10) and 48% for small-volume items (n=5) indicating a decrease in accuracy with smaller volumes. |
| Anzawa, et al. (2019) | For calories/energy estimation: All the buffet dishes have their nutrition information. Dish was assumed to contain one serving size. Dishes were summed for calculating total nutrition. | None | Mean absolute error (MAE); correlation coefficient | **MAE (kcal or g with %) of estimated nutrients for single-class, multi-class, and hierarchical recognition techniques using the JISS-22 database (assumes single servings for buffet items):**  [Energy (kcal)]  Single-class recognition = 106 (13.8%)  Multi-class recognition = 152 (20.2%)  Hierarchical recognition = 74 (9.4%)  Correlation between estimated calories (kcal) via hierarchical recognition and ground truth = 0.92  [Protein (g)]  Single-class recognition = 4.93 (11.9%)  Multi-class recognition = 8.76 (21.0%)  Hierarchical recognition = 4.04 (9.6%)  [Lipid (g)]  Single-class recognition = 4.27 (16.3%)  Multi-class recognition = 6.77 (24.8%)  Hierarchical recognition = 3.72 (14.3%)  [Carbohydrate (g)]  Single-class recognition = 14.0 (16.2%)  Multi-class recognition = 18.1 (22.6%)  Hierarchical recognition = 8.09 (9.9%) | When applied to real data from buffet-style meals (food images from the JISS dataset), the hierarchical scheme outperformed single- and multi-class recognition techniques by achieving lower errors in estimated energy (9.4%), protein (9.6%), lipids (14.3%), and carbohydrates (9.9%) while also showing a high correlation between estimated and ground truth calories (0.92). |
| Ege and Yanai (2019) | For calories/energy estimation: recipe data with food calorie information for one person | None | Absolute error; relative error; ratio of estimated value within the relative error of 20% and 40% | **Error of calories estimated by single-task, multi-task, or the proposed method (detection + calorie estimation) using calorie-annotated single-dish food photos from 15 food categories:**  [Absolute (relative) error; ratio of the estimated value within the relative errors of 20%]  Single-task = 105.7 kcal (30.2%); 43%  Multi-task = 94.1 kcal (27.9%); 48%  Detection + Calorie estimation = 89.4 kcal (26.6%); 51% | The proposed method (detection + calorie estimation) was more accurate than single- and multi-task models for food calorie estimation based on lower error (27%) and a higher ratio of estimated values within the 20% error bound (51%). |
| Fang, et al. (2019) | Pre-weighed food packs were distributed to the participants and uneaten foods were returned and weighed; consumption estimates were based on calculations using weighed food and the energy values in the USDA Food and Nutrient Database for Dietary Studies | None | Absolute error | **Absolute error (kcal) for estimated energy:**  [Reported absolute error (calculated relative error)]  Average overall error = 209 (38.3%)  Breakfast = 204 (38.4%)  Lunch = 211 (35.0%)  Dinner = 210 (41.5%) | The proposed end-to-end system directly estimated food energy using automatic food portion estimation from eating occasion images captured with an image-based system and achieved an overall absolute error of 209 kcal (~38.3%). |
| Makhsous, et al. (2019) | For volume estimation: water displacement | None | Percentage of error | **Error for volume estimation using the DDRS algorithm:**  Overall percentage of error across 20 food items = ~11%  **Error for volume estimations converted to calorie calculations using the NDSR database:**  Overall percentage of error across 20 food items = ~15% | This pilot study of the DDRS smartphone application found overall percentage error in volume and calculated calorie estimations around or below 15% and reported improved accuracy over manual human estimations. |
| Situju, et al. (2019) | For calories/energy and salinity estimation: Ingredient annotated dataset: "calorie and the salinity information provided on these recipe sites are for one person"; NR for category annotated dataset | None | Absolute error; relative error; correlation coefficients | **Performance of calorie (kcal) estimations by single-task CNN, multi-task CNN, and the proposed multi-task CNN with two-stage fine tuning:**  [Absolute (relative) error overall; range of relative errors across 14 food categories; correlation between category classification and calorie estimation overall]  Single-task CNN = 100.2 kcal (41.7%); 12.7% (chow mein) to 104.9% (miso soup); 0.80  Multi-task CNN = 94.6 kcal (36.6%); 13.9% (chow mein) to 81.2% (miso soup); 0.82  Multi-task CNN with two-stage fine tuning = 89.6 kcal (31.2%); 11.4% (chow mein) to 55.6% (miso soup); 0.84  **Performance of salinity (g) estimations by single-task CNN, multi-task CNN, and the proposed multi-task CNN with two-stage fine tuning:**  [Absolute (relative) error overall; range of relative errors across 14 food categories; correlation between category classification and salinity estimation overall]  Single-task CNN = 0.75 g (37.2%); 22.8% (chow mein) to 59.4% (beef and potato stew); 0.40  Multi-task CNN = 0.76 g (36.8%); 21.1% (chow mein) to 67.9% (beef and potato stew); 0.43  Multi-task CNN with two-stage fine tuning = 0.74 g (36.1%); 18.5% (curry and rice) to 62.7% (beef and potato stew); 0.45 | Using a newly constructed dataset of publicly available images from several recipe-gathering websites, the proposed multi-task CNN with two-stage fine tuning had better overall performance for estimated calories (31.2% error) and salinity (36.1% error) compared with single-task CNN and multi-task CNN. Correlations between category classification and nutrients were also better with the two-stage model, but for all three models, estimated calories had much higher correlations (0.80-0.84) than estimated salinity (0.40-0.45). |
| Herzig, et al. (2020) | For weight estimation: precision scale  For macronutrient estimation: Conversion into macronutrient content was performed using the Swiss Food Composition Database | None | Absolute error; relative error; bias; 95% limits of agreement | **Errors of estimated weight, macronutrients, and energy content for all meals (breakfast, cooked meals, snacks):**  [Mean (SD) absolute error; mean (SD) relative error]  Weight (g) = 35.1 (42.8); 14.0% (12.2%)  Carbohydrate (g) = 5.5 (5.1); 14.8% (10.9%)  Protein (g) = 2.4 (5.6); 13.0% (13.8%)  Lipids (g) = 1.3 (1.7); 12.3% (12.8%)  Energy (kcal) = 41.2 (42.5); 12.7% (10.8%)  **Bias of estimated weight, macronutrients, and energy content for all meals (breakfast, cooked meals, snacks):**  [Mean (SD) absolute bias; mean (SD) relative bias; 95% limits of agreement (g or kcal)]  Weight (g) = 19.3 (52.1); 5.4% (17.8%); -84.8 and 123.4  Carbohydrate (g) = 1.0 (7.5); 2.9% (18.3%); -13.9 and 15.9  Protein (g) = 1.7 (5.9); 5.6% (18.2%); -10.0 and 13.4  Lipids (g) = 0.5 (2.1); 5.7% (16.9%); -3.8 and 4.7  Energy (kcal) = 15.5 (57.4); 4.1% (16.2%); -99.4 and 130.3  **Influence of viewing angle and meal type (breakfast, cooked meals, or snacks) on the accuracy of estimated weight, macronutrients, and energy content:**  [Viewing angle *P*-values for absolute errors; Meal type *P*-values for absolute errors]  Weight = 0.96; 0.001  Carbohydrate = 0.83; 0.001  Protein = 0.99; 0.001  Lipids = 0.73; 0.002  Energy = 0.70; 0.005 | A smartphone app combining depth sensing with computer vision and using volumetry had relative errors ≤13.8% for estimated weight, macronutrients, and energy from a broad set of meals in a real-life setting.  Meal type, but not viewing angle, significantly influenced accuracy of macronutrient and energy estimation such that bias for *cooked meals* was significantly higher (*P*<0.001) than for *breakfast* and marginally higher (*P*<0.1) than for *snacks* for all estimated macronutrients. Bias for *snacks* and bias for *breakfast* were not different for estimates of weight, any macronutrients, or energy. |
| Jiji and Rajesh (2020) | For calories/energy estimation: nutritional facts database | None | Actual vs. estimated calories | **Calorie estimation using two different illuminations, angles, and cameras:**  [Calculated absolute (relative) errors for measure 1 (location 1, 30º angle, Sony camera); measure 2 (location 2, 90º angle, Samsung camera)]  Carrot = 2.44 (6.0%); 1.55 (3.8%)  Apple = 2.87 (5.5%); 1.77 (3.4%)  Tomato = 0.54 (3.0%); 1.73 (9.6%)  Pomegranate = 2.02 (2.4%); 1.56 (1.9%)  Potato = 0.77 (2.2%); 2.13 (6.1%) | The proposed system showed <10% relative error in calorie estimation across five different foods with images produced by different illumination, camera angle, and mobile phone camera. |
| Lo, et al. (2020) | For volume estimation: "The food items are placed on an automatic turning table, which keeps rotating while the depth camera is recording. The3-D models of real food items are constructed and the ground truth volume can then be obtained using RecFusion, a professional 3-D scanning system, which performs dense 3-D reconstruction and volume estimation" | None | Accuracy | **Accuracy (%) for volume estimation at testing stage for fully-connected (light-weight) and UNet (extended) architectures:**  [Average (SD) accuracy (%) calculated based on testing dataset with 10 categories, each with 100 models, and each model with 20 different viewing angles (20 k partial inputs in total)]  Fully-connected = 90.45 (7.49)  UNet = 92.29 (5.12)  **Accuracy (%) for volume estimation by VNet trained on datasets with and without data augmentation (generated through linear interpolation):**  [Average accuracy (%) across 8 categories]  VNet without augmentation = 66.12  VNet with augmentation = 82.90  **Accuracy (%) for volume estimation by 3-D reconstruction with and without UNet:**  [Average accuracy (%) across 11 categories]  3-D reconstruction only = 71.54  3-D reconstruction with UNet = 84.68 | Using the proposed point completion network, UNet, the point cloud of occluded food items can be completed using prior learned shapes, and food volume estimation can achieve up to 92.29% accuracy. |
| Lu and Stathopoulou, et al. (2020) | For MADiMa database: "The pixel-level semantic map of the meal images are manually annotated and the ground truth calorie and macronutrient content for each food item are calculated from the recorded food weight and food composition databases (USDA and/or Swiss)"; for the fast food database: "The ground truth calories and macronutrient content were retrieved from the official website of McDonald" | None | Mean absolute relative error (MARE); median absolute error and its 25th and 75th percentiles; Pearson correlations | **MARE (%) for food volume estimation by the proposed joint learning architecture (i.e., goFOOD™) compared to GoCARB:**  [Using the MADiMA database with 234 food items]  goFOOD™ = 19%  GoCARB system = 22.6%  **Pearson correlations (*r*) for nutrient estimations by goFOOD™ vs. dieticians vs. ground truth (GT) using different databases (*P* < 0.05 for all reported values):**  [MADiMa database: goFOOD™ vs. GT; dieticians vs. GT; goFOOD™ vs. dieticians]  Carbohydrates (g) = 0.54; 0.57; 0.40  Protein (g) = 0.69; 0.82; 0.62  Fat (g) = 0.66; 0.63; 0.47  Calories (kcal) = 0.60; 0.66; 0.52  [Fast Food database: goFOOD™ vs. GT; dieticians vs. GT; goFOOD™ vs. dieticians]  Carbohydrates (g) = 0.88; 0.97; 0.87  Protein (g) = 0.89; 0.95; 0.92  Fat (g) = 0.50; 0.94; 0.47  Calories (kcal) = 0.87; 0.97; 0.83 | The proposed goFOOD™ system showed improved accuracy in volume estimations compared to previous versions (MARE = 19% vs. 22.6% previously).  For images of normal central-European meals (MADiMa database), nutrient estimates by goFOOD™ correlated more strongly with ground truth than estimates by experienced dietitians; goFOOD™ and dieticians performed similarly for fast food standardized meals (Fast Food database). |
| Makhsous, et al. (2020) | For volume: a weighing scale and manual measurement tools (i.e., measuring cups); plates were measured before and after the meal | None | Absolute error (g and %) | **Absolute error (g and %) for volume estimation by ASA24, MyFitnessPal, or the DietSensor system:**  [Mean (SD) absolute error in g; absolute error (%)]  ASA24 (n=13 meals) = 289 (34); 51%  MyFitnessPal (n=8 meals) = 390 (28); 73%  DietSensor (n=13 meals) = 255 (14); 33% | When tested by a small group of participants, DietSensor achieved better volume estimation accuracy (33% error) than when participants used the ASA24 (51%) or MyFitnessPal (73%). |
| Mohideen Pillai and Kother Mohideen (2020) | Nutrient tables, but no ground truth mentioned for volume (although, error% was reported, and it is possible the GT volume was based on measurements of photos | None | Error; uncertainty of measurement | **Error percentage for area estimates for select foods by the proposed method:**  Bread = 0.63%  Cake = 2.30%  Spaghetti = -3.07%  Cookies = 0.50%  Omelet = 10.5% | The proposed territorial measurement method estimated area for different food types with error percentages ≤11%. |
| Shermila and Milton (2020) | For weight: a notebook-weighing machine | None | Average prediction errors | **Average prediction errors for protein predictions from health drink powders by separate image features (FOS, HOG, LBP, GLCM, and Prewitt) or by deep CNN with experiments conducted for separate image components:**  [Single image feature prediction ranges]  Full color component = ± 3.02 (FOS) to ± 3.60 (HOG)  Red color component = ± 2.80 (GLCM) to ± 3.39 (HOG)  Green color component = ± 2.80 (GLCM) to ± 3.48 (HOG)  Blue color component = ± 2.71 (GLCM) to ± 3.14 (HOG)  Gray component = ± 2.80 (GLCM) to ± 3.98 (HOG)  [Deep CNN trained on the steepest descent algorithm with 30 epochs; 40 epochs and at a learning rate of 0.001]  Full color component = ± 2.13; ± 1.96  Red color component = ± 2.13; ± 2.12  Green color component = ± 2.03; ± 2.01  Blue color component = ± 1.97; ± 1.98  Gray component = ± 2.04; ± 2.01 | Using the GLCM image feature and blue color image component to model the linear regression system produced a minimum average protein prediction error of ± 2.71. In analyses of combined image features, FOS + GLCM performed best overall across image components, texture features (e.g., LBP and GLCM) were good at predicting protein content, and HOG predicted protein with maximum average error.  Using deep CNN to model the regression system in the full color component produced a minimum average protein prediction error of ± 1.96. |
| Chotwanvirat, et al. (2021) | For food portion: a digital kitchen scale and rounded to the nearest integer | Eight RDs from Theptarin Hospital and six RDs who were certified diabetes educators by the Thai Association of Diabetes Educators. Five had performed image-assisted dietary assessment in a previous study. Results were converted back to weight in grams for identified foods according to the Thai Food Exchange List or the official Carbohydrate Counting guidebook. | RMSE, Pearson's correlation coefficient (r), Lin's concordance correlation coefficient (Rc), paired t-test, and Bland-Altman plot | Estimated carbohydrate content was 655 grams compared to 682 measured, error rate = 4%  Root mean square error between Measured and Estimated carbohydrate content was 9.4.  Lin's concordance correlation coefficient was 0.79 and Pearson's correlation coefficient was 0.80.  Mean of the carbohydrate content of Measured (30.3 +- 14.8 g) and Estimated by the system (29.2 +- 15.3) was not statistically significantly different (t test p > .05) | Deep learning based carbohydrate estimation using Thai food images was comparable to dietitians', with an overall error percentage of 4% (682 grams estimated vs. 655 grams measured). |
| Kumar, et al. (2021) | For food volume: Top and side view of the images are used to calculate food volume by measuring length, width, height and depth. Calorie values of the food are based on multiplying food mass with the “normalized” calorific level. Database NR. | None | Sample calories; calorie estimation | **Calorie estimation using SVM- and MLP-based classifiers for 6 food classes:**  [Calculated absolute (relative) errors for SVM; MLP]  Apple = 9 (17.3%); 5 (9.6%)  Banana = 5.25 (22.1%); 1.25 (5.3%)  Bread = 8 (10.7%); 5 (6.7%)  Guava = 1 (1.3%); 3 (3.8%)  Pizza = 6.7 (8.2%); 1.7 (2.1%)  Pomegranate = 5.4 (25.0%); 1.4 (6.5%) | MLP-based classifiers predicted calories with less error than SVM-based classifiers for 5 out of 6 food classes and produced relative error <10% for all 6 food classes. |
| Lu, et al. (2021) | For weight: scales with the unit of gram.  For nutrient intake of one served food: annotation using the weight and nutrient information provided by the hospital kitchen  For nutrient intake of multiple served foods: the iterated plate weight difference and the visual estimation approach are adopted for nutrient annotation | 2 assessors (dietitians from the USA, with over 5 years of experience in macronutrient counting), performed visual estimations for macronutrients (in grams) and calories (in kcal) for each meal and report them on a dedicated excel file (both for the MADiMa and the fast food databases) | Mean absolute error (MAE); mean relative error (MRE); and correction coefficient | **Performance of nutrient intake estimation by the proposed system with and without a daily menu:**  [*With daily menu*: MRE (%); correlation coefficient (*r*) for proposed system vs. ground truth (*P*<0.001 for all)]  Calories = 14.84; 0.923  Carbohydrates = 18.16; 0.937  Fat = 19.86; 0.927  Protein = 17.00; 0.921  Salt = 17.72; 0.941  Fiber = 19.42; 0.910  [*Without daily menu*: MRE (%); correlation coefficient (*r*) for proposed system vs. ground truth (*P*<0.001 for all)]  Calories = 18.52; 0.824  Carbohydrates = 19.12; 0.914  Fat = 27.70; 0.682  Protein = 19.34; 0.889  Salt = 20.20; 0.808  Fiber = 21.99; 0.883 | When used without a daily menu, the proposed system showed low error in calorie and all nutrient estimates (19-28%). Using a daily menu improved system performance (all estimates <20% error) and showed a “very strong” correlation (r>0.9, p<0.001) with ground truth. |
| Ma, et al. (2021) | For food portion: Nutrition information in one portion of food according to database | None | Random sample consensus (RANSAC) regression line; slope of RANSAC regression, mean absolute percentage error (MAPE), pearson correlation coefficient, spearman correlation coefficient | **Regression line for estimated nutrients by Top-5 AM (post-arithmetic mean by top-5 normalization):**  Inception V3 (proposed model) R2 range = 0.6362 (Fiber) to 0.7300 (protein)  WISeR-50 R2 range = 0.5645 (Iron) to 0.6613 (Calcium)  Resnet-50 R2 range = 0.4608 (Fiber) to 0.5714 (Calcium)  **Mean Absolute Percentage Error from Inception V3 (proposed model; numbers pulled from Figure 4 using https://automeris.io/WebPlotDigitizer/index.html)**  Range = 0.61423 (Iron) to 0.69717 (Vitamin C)  Overall average (SD), calculated by authors from reported numbers = 67% (SD = 0.04)  Pearson's correlation coefficient for Inception V3  Range = 0.78977 (Fiber) to 0.85097 (Protein)  Spearman correlation for Inception V3  Range = 0.79302 (Fiber) to 0.84124 (Protein)  Rohatgi, A. WebPlotDigitizer, Version 4.6, September 2022, https://automeris.io/WebPlotDigitizer, E-Mail: ankitrohatgi@hotmail.com; Location: Pacifica, California, USA | The results showed that the top-5 Arithmetic Mean (AM) algorithm achieved the highest regression coefficient (R2) up to 0.73 for protein estimation.  The RANSAC results showed that the nutrient distributions impacted the final estimation accuracy, the more balanced, the higher accuracy. The results achieved by deep learning for food nutrient estimation could encourage AI to be applied to the field of food in the future. |
| Papathanail, et al. (2021) | NA - human assessor | Trained medical student, dietitians, and instructed nursing staff | Mean absolute error, mean relative error %, correlation coefficient, error % | **Comparative results for the energy and the macronutrient intake for the testing set by the system and by a nurse using standard clinical procedure:**  **[System: Mean absolute error (sd), mean relative error %, correlation coefficient]**  Energy (kcal)=41 (54), 11.64%, 0.967  Carbohydrate (g)=4.6 (8.3), 13.23%, 0.905  Protein (g)=1.4 (2.5), 10.47%, 0.979  Fat (g)=1.9 (2.4), 11.70%, 0.984  Fatty acids (g)=1.2 (1.4), 14.84%, 0.978  **[Nurse: Mean absolute error (sd), mean relative error %, correlation coefficient]**  Energy (kcal)=112 (102), 31.45%, 0.861  Carbohydrate (g)=9.0 (10.8), 33.88%, 0.790  Protein (g)=3.7 (4.1), 32.34%, 0.919  Fat (g)=7.0 (6.4), 41.29%, 0.877  Fatty acids (g)=4.1 (3.7), 56.42%, 0.841  **Error (%) for consumed percentage by the system and by a nurse using the standard clinical procedure:**  [System error % vs. nurse error%]  Soup=8.08% vs. 24.04%  Side dish=9.50% vs. 12.67%  Meat/fish=6.56% vs. 19.61%  Salad/vegetables=7.46% vs. 21.50%  Dessert=10.74% vs. 34.67% | It shows that the AI and standard clinical were compared to human assessors (as GT), and pull either mean relative error % . |
| Yang, et al. (2021) | generated volume (for virtual food databases); ideal real food database: NR, but foods were standard meals from cafeteria; general real food database: applied method to the GRFD dataset which contained 416 images with measured volumes; authors refer to "volumetric truths" throughout. | None | Mean relative volumetric error (mRVE) | **mRVE (%) for volume estimations by the proposed human-mimetic method using the VFDL and VFDS datasets:**  [Overall mRVE (range) across 15 class divisions (1-15, small to large volume classes) for top1 (soft predictions) and top3 (hard predicted label)]  VFDL-15, top1 = 8.7 (5.0 [class 14] to 15.1 [class 1])  VFDL-15, top3 = 9.6 (5.7 [class 14] to 15.9 [class 1])  VFDS-15, top1 = 8.7 (5.3 [class 15] to 19.6 [class 1])  VFDS-15, top3 = 9.4 (5.1 [class 15] to 19.8 [class 1])  [mRVE using 30 reference classes]  VFDL-30, volume intervals of 100 mL between neighboring reference classes = 8.7  VFDS-30, volume intervals of 50 mL between neighboring reference classes = 8.6  [mRVE using mixed training data of the VFDL-15 and VFDS-15 datasets]  VFDL-15 = 8.5  VFDS-15 = 8.5 | Using the proposed normalization method showed consistent accuracy (≤20% error) across food classes of different volumes. Volume estimation errors in large volume classes were typically smaller than errors in small volume classes.  Results from experiments with mixed training datasets suggested food images can be placed in the same class if they share similar normalized volumes, regardless of actual volumes. |
| Yuan, et al. (2021) | For food volume: water displacement  "the true volumes of the 3D objects are precisely known"; "The control unit has an option to tare the plate weight automatically to obtain the net weight of the food." | None | Error | **Error (%) for volume estimations for real food by different methods:**  [Ranges across 6 different foods]  Slice-based method (N=20, cm^3^) = 8.5% (stir fry) to 34.8% (burger)  Slice-based method (N=50, cm^3^) = 3.3% (stir fry) to 26.7% (burger)  Image-based electric field method = 0.83% (apple) to 5.23% (stir fry)  Laser-based electric field method = 3.07% (bread) to 8.70% (apple) | When estimating volume for real foods, the electric field-based method outperformed the slice-based method. With this method, a best number of slices (*N*) can be found to optimize volume estimation, but *N* can vary for different foods.  In the image-based and laser-based point clouds, the electric field method produced volume estimations with low errors (all ≤9%) for all food shapes. |
| Dai, et al. (2021) | The calorie of one roll of basic Gimbap is defined as 35 - 40 kcal. One roll is sliced into 10-12 pieces for a typical total of 350 – 400 kcal. | None | Accuracy % | **Percentage error rate for estimated calories (calculated by authors):**  **Note: The "ground truth" for each slice of Gimbap is 35-40 kcal, for a total of 350-400 per roll [of 10].**  Figure 10(b1) = 282.38 kcal for 8 pieces  Figure 10(b2) = 269.99 kcal for 6 pieces  Figure 10(b3) = 390.84 kcal for 10 pieces (with some additional contents spilling out of the side, which would mean a few more kcals)  **Average precision (AP) (0.5 IoU)**  Gimbap1 = 88.13%  Gimbap2 = 82.72%  **mAP (mean average precision) at 0.5 IoU = 85.43%** | After training, the model shows accuracy of 88.13% for GIMBAP1 and that of 82.72% for Gimbap2. The fine-tuning Mask R-CNN achieved improved food segmentation and enhanced calorie estimation. |
| Kadam, et al. (2022) | Publicly available information posted online by a blogger named "Unknown" at blogger.com | None | Accuracy, % error, sensitivity, specificity, precision, negative predictive value, F1 score, Matthews correlation coefficient, false positive rate, false discovery rate, fast negative rate | **Volume estimation for four different scenarios/shapes of food items or bowls (actual volume / estimated volume; accuracy %):**  Amorphous: 270.165 / 299.154; 90.46%  Convex: 441.036 / 485.14; 90.9%  Regular (square/bread): 130 / 132.1; 98.5%  Regular (circle/orange): 79.0321 / 78.125; 98.9%  **Calorie estimation for four different scenarios/shapes of food items or bowls (actual calories / estimated calories; accuracy %):**  Amorphous: 660 / 733; 90.05%  Convex: 1078 / 1185; 90.98%  Regular (square/bread): 344 / 349.8; 98.4%  Regular (circle/orange): 36.718 / 37.145; 98.9% | This work supports the accuracy of MaskRCNN in food image segmentation, enhanced with a pre-trained RESNET network. The volume of the food is calculated with an accuracy of 90.46% and convex-shaped food items, like those shaped food like an upside down bowl, achieve an accuracy of 90.0%. |
| Li, et al. (2022) | Weight of the food multiplied by nutrition info from the databases | None | Accuracy %; Precision; Mean area under the PR curve (mAP) | **Percent accuracy for estimated nutritional composition (combination of carbohydrate, protein, fat, fiber, energy/calories, carotene, Vitamins A, E and C) across different eating scenarios and food image contents:**  Total average nutrition composition accuracy was 90.1%  Avg. for dataset a (one kind of food in image) in eating scenario C1 (1 person eats 1-3 foods) = 89.7%  Avg. for dataset b (two kinds in image) across eating scenarios C1 and C2 (includes 2 people and 2-4 foods) = 92.5%  Avg. for dataset c (3 kinds) across 3 scenarios = 93.3%  Avg. for d (4 kinds) across 3 scenarios = 97.2%  Avg. for e (5 kinds) across 3 scenarios = 96.5%  Avg. for f (6 kinds) across 2 scenarios = 80.9%  Avg. for g (9 kinds) for 1 scenario (5 people eat 6,8.9 foods) = 80.3% | In the scenarios where 3 or 4 people eat four foods and six foods, the nutritional composition perception of the system was the most accurate. On the whole, the nutritional composition perception accuracy of the system was good, with an average accuracy of 90.1%. |
| Ma, et al. (2022) | Database was annotated with nutrient information | None | Spearman correlation, Pearson's correlation, R2, Slope, Mean Absolute Percentage Error | **top-5 AM** had the highest regression coefficients (R2), up to 0.7748 for iron content estimation and lowest for thiamin 0.5410.  **Mean absolute percentage error for estimated calories (kcal/100g; assuming flipped to indicate % accuracy):**  Arithmetic mean model = 79.6%  Normalization+AM model = 76.6%  Harmonic mean model = 36%  **Pearson’s correlations for estimated calories:**  Arithmetic mean model = 0.83  Normalization+AM model = 0.80  Harmonic mean model = 0.79  **Spearman’s correlations for estimated calories:**  Arithmetic mean model = 0.80  Normalization+AM model = 0.80  Harmonic mean model = 0.81 | Proposed a suite of big-data-driven DL models regressing from food images to their nutrient estimation. Intercept V3 achieved the highest accuracy for nutrients estimation, which was acceptable for practical application |
| Minjia, et al. (2022) | Unclear but seems the database used was annotated | None | Macro Average Accuracy (MAA)  Standard Accuracy (SA)  Mean Square Error (MSE) | Macro Average Accuracy (MAA): 0.9643  Standard Accuracy (SA): 0.9877  Mean Square Error (MSE): 1816.9 | The proposed method outperforms the existing methods and attained 0.9643 for MAA, 0.9877 for SA, and 1816.9 for MSE. |
| Pfisterer, et al. (2022) | Ground-truth hand segmentation and an "applied ground truth" through the graph cut semi-automated method | NA | Mean absolute error; Volume intake error; mean error bias; mean error | **Volume estimation accuracy (mL) from comparative analyses of system performance within and across LTC datasets for the proposed method (EDFN, EDFN-D) and â€œapplied ground truthâ€ graph cuts (GC, GC-D):**  Regular texture foods  [Mean (SD) for mean absolute error; mean error bias; volume intake error]  EDFN=17.1 (49.2); -14.7 (50.0); -129.2 (154.3)  EDFN-D=18.0 (50.0); -17.2 (50.3); -130.2 (154.8)  GC=4.5 (5.5; -0.0 (7.1); 1.8 (6.6)  GC-D=4.6 (5.4); -1.8 (6.9); 0.2 (6.5)  Modified texture foods  [Mean (SD) for mean absolute error; mean error bias; volume intake error]  EDFN=2.8 (3.1); 1.7 (3.8); 0.3 (3.6)  EDFN-D=2.3 (3.2); -0.7 (3.9); 0.8 (3.6)  GC=2.2 (2.7); -1.9 (2.9); -0.9 (3.3)  GC-D=3.2 (3.4); -3.1 (3.5); -0.5 (3.4)  **Volume estimation accuracy (mL) across portion sizes (P#) for the modified texture foods dataset from the proposed methods (EDFN and EDFN-D):**  EDFN (no-depth-refinement)  [Mean (SD) for mean absolute error; mean error; volume intake error]  P1=3.3 (3.4); 2.0 (4.3); 0.0 (0.0)  P5=2.0 (2.6); 1.1 (3.1); 0.9 (4.6)  EDFN-D (depth-refined)  [Mean (SD) for mean absolute error; mean error; volume intake error]  P1=2.6 (3.3); 0.1 (4.3); 0.0 (0.0)  **P5=1.7 (2.2); -1.5 (2.4); 1.6 (4.0)** | Both proposed systems with (EDFN-D) and without (EDFN) depth-refinement had lower mean absolute errors and volume intake errors for images of modified texture foods compared to images of regular texture foods (including salad with a low food density). Both systems had higher volume intake errors as portions sizes decreased. Absolute mean absolute error was low overall (less than 4.0 mL).  Compared to the proposed methods, ground truth methods had greater volume estimation accuracy for regular texture foods, but similar volume estimation accuracy for modified texture foods. |
| Prakash, et al. (2022) | Ground truth | NA | Accuracy % | **Estimates for classification (for fruits), calories, protein, fat and carbohydrates were combined and compared between CNN and Logistic Regression methods:**  Overall average accuracy % for CNN = 83.84 %  Iteration (of 10) with highest % accuracy for CNN = 92.8% or 94.6%  Iteration (of 10) with lowest % accuracy for CNN = 78% or 85.4%  *Two numbers were presented in table vs. text but could not contact author for resolution | On a combined indicator representing fruit classification, calories, protein, fat and carbohydrates, the CNN model was significantly more accurate than the Logistic Regression model. The average accuracy for CNN was 83.84% and was 72.30% for Logistic Regression. |
| Sasaki, et al. (2022) | Dietitians observed the cooking process and recorded nutrient and food group contents of the 120 sample meals | Nationally registered dietitians  "After the app automatically predicted nutrition and food content of the meals, the staff were allowed to manually modify the name and portion size of each item based on their visual inspection. If the staff found that some ingredients needed to be added or removed, they modified the outputs from the app and recorded them (data Y)." | Means of the difference using paired t-test; Bland-Altman plots | **Mean (Standard Deviation) for Ground Truth and AI estimated meals (n = 120):**  Weight, GT = 524 g (129); Weight, AI= 521 g (156); Difference = -4; p value = 0.72  Energy, GT = 562 kcal (191); Energy, AI = 506 (189); Difference = -57, p value = < .001  Smallest difference between methods across a wide range of nutrients = -0.03 Vitamin B2  Largest difference between methods across a wide range of nutrients = -157 sodium  **Comparison of estimations for weight, energy, and macronutrient estimations by the automatic and manually adjusted system vs. ground truth (GT) for 120 sample meals (see [ref #] for micronutrient results):**  **[absolute mean difference (p-level) for automatic vs. GT; manually adjusted vs. GT]**  Weight (g)=-4 (0.72); 48 (<0.001)  Energy (kcal)=-57 (<0.001); 9 (0.40)  Protein (g)=-3.5 (<0.001); -0.4 (0.40)  Fat (g)=-1.9 (0.02); 0.5 (0.43)  Carbohydrate (g)=-4.3 (0.02); 1.5 (0.27) | Automatic estimations by the CALO Mama app (without manual adjustment) were statistically similar to ground truth (p>0.05) for 36.7% of 30 estimated nutrients. Manual adjustments to the automatic process improved accuracy such that estimates matched ground truth for 96.7% of 30 nutrients. |
| Tagi, et al. (2022) | For weight: weight method, digital scale, on an 11-point scale ranging from 0 to 10 | 10 dietitians from Tokushima University Hospital and 6 students from the Dept. of medical Nutrition, Tokushima University.  assessors used an 11-point scale (0 is <= 5% consumed to 11 = >= 95% consumed) to indicate consumption by viewing digital images | Mean absolute error, RMSEA, Bland-Altmans, coefficient of determination (R2), Welch t test, and the confusion matrix | **Measured value (volume) of leftover liquids / Estimated value / p-value / percent accuracy:**  Thin rice gruel: 4.58 / 3.39 / < .001 / 99.86%  Fermented milk: 4.58 / 5.15 / <.001 / 99.88%  Peach juice: 4.58 / 4.53 / .35 / 99.99%  Total: 4.58 / 4.15 / <.001 / 99.91%  **Mean absolute errors for the AI estimation of volume compared to weighed (i.e., actual measured) values:**  Thin rice gruel: 0.99  Fermented milk: 0.63  Peach juice: 0.25  Total: 0.85 | The AI estimation approach achieved higher accuracy than the visual estimation approach, suggesting that the AI estimation approach is more reliable for the precise measurement of liquid food intake. |
| UlHaque, et al. (2022) | Diet data (grams and Kilocalories) implicit in Food-101 and Fruit-360 | NA | Accuracy percent | **Accuracy percentage**  Calories for Model 44 (Training): 0.84  Calories for Model 44 (Validation): 0.86  Calories for Model 44 (Test): 0.848  Calories range for Models in Table 5 (for 10 models): Highest (Training): 0.84  Lowest (Training): 0.795  Highest (Validation): 0.86  Lowest (Validation): 0.847  Highest (Test): 0.85  Lowest (Test): 0.829 | The best overall model achieved 84.8% accuracy in the test phase |
| Zhang, et al. (2022) | Based on the type of food and the area of the food in the real world, a machine learning algorithm was built to infer the weight of the food | NA | t-test, Pearson correlation, R^2 | **Correlations for actual measurements and estimated values:**  Area: r=0.979, P=0.01  Weight: r=0.936, P=0.01 | Using area-weight calculation models, the proposed method's estimates of area and weight from food images had strong positive correlations with actual area and weight (r=0.98 and r=0.94, respectively). |
| Nadeem, et al. (2023) | Database had known calories -- i.e., calories per 100 grams of food, food density, and food shape | NA | Accuracy %, average calories (and actual calories) | **Percent accuracy for calories:**  Coca-Cola can: mean accuracy = 60% (average estimated calories = 153; actual calories = 142)  Apple: mean accuracy = 92% (average estimated calories = 77; actual calories 78)  Orange: mean accuracy = 90% (average estimated calories = 53; actual calories = 58) | The proposed mobile application, Smart Diet Diary improves semi-automatic calorie estimation using deep neural networks for object classification and detection. The experimental results indicated that the proposed system has an overall accuracy of around 80% and calorie count was within a 10% range of actual value. |
| Shao, et al. (2023) | Nutrition5K database, and authors meticulously screened each image and checked the nutrient information against the USDA FND database | NA | percentage of mean absolute error (PMAE) | **Percentage of mean absolute error (PMAE) for the proposed method on the Nutrition5k dataset:**  **[PMAE on whole dataset; PMAE after problem dishes were removed from dataset]**  Calories (kcal): 15.0%; 14.1%  Mass (g): 10.8%; 10.5%  Fat (g): 23.5%; 22.1%  Carbohydrates (g): 21.8%; 21.8%  Protein (g): 21.0%; 19.6%  Mean PMAE when whole dataset was used: 18.5% | Compared to select backbone networks and loss functions, the proposed method showed lower percentage of mean absolute error (PMAE) for estimated calories, mass, and carbohydrates but higher PMAE for fat and protein compared to RDFNet+CBAM Google-Nutrition. In the proposed method, the PMAE value of calories, mass, fat, carbohydrate, and protein reached 14.1%, 10.5%, 22.1%, 21.8%, and 19.6%, respectively. |
| Zheng, et al. (2023) | Averaging multiple water displacement measurement | NA | Error % | **Error % for volume estimation compared to water displacement:**  egg = 0.58%  orange = 0.62%  chicken leg = 34.20%  bread = 11.82%  grapefruit = 6.20%  cake = 12.00%  peach = 14.99% | The food volume accuracy for individual food replicas (e.g., an egg) compared to water displacement was generally high (0.58% for egg), except for the chicken leg, especially for regularly shaped foods, like eggs. Estimation for asymmetrical and thicker foods was higher.  The model-based food volume estimation method and other manual interactive method have the highest accuracy, but this algorithm requires manually selecting and manipulating three-dimensional objects, which are tedious and difficult to use in practice. |

CNN = convolutional neural network; CSW-WLIFC = Cauchy, Generalized T-Student, and Wavelet kernel based Wu-and-Li Index Fuzzy clustering based segmentation; FOS = first order statistics; GLCM = gray level co-occurrence matrix; HOG = histogram of oriented gradient; LBP = local binary pattern; MLP = multilayer perceptron; NN = neural network; NR = not reported; SVM = support vector machines; WLM = Whale Levenberg Marquardt model.

^a^ In papers reporting accuracy results, Absolute error was calculated as $|estimate-ground truth$|; Relative error (%) was calculated as $(|estimate-ground truth|\div ground truth)\times100$; Bias was calculated as $(estimate-ground truth$); and 95% limits of agreement were calculated as $(\pm2\times SD of the bias)$. When accuracy results were not reported in a paper but both measured and estimated values were reported, we performed and reported calculations for absolute and relative errors based on the formulas above.

^b^ Rhyner, et al. (2016) and Vasiloglou, et al. (2018) report on different studies using the same tool.

**eTable 5**. Food image characteristics for each study by year

| **Author (year)** | **Image setting (real-world or lab)^a^** | **Image content and type of food^b^** | **Image content:**  **Number of foods per image^c^** | **Image content:**  **Whether and how foods were arranged^d^** | **Image content:**  **Number of different food types analyzed** | **Image content:**  **Number of different items analyzed per food category** | **Number of images per food item** | **Number of newly captured images assessed** | **Number of database images assessed (by process, if more than one)** |
| --- | --- | --- | --- | --- | --- | --- | --- | --- | --- |
| Zhu, et al. (2010) | Mostly controlled (e.g., Real-world with clear table and solid background) | Standardized (e.g., chain restaurant, cafeteria, packaged foods) | Single and multiple food item images | Mostly controlled (e.g., arranged, but items are close together on a plate/tray) | Multiple | NR | Not clear, but presumably 2 (before and after meal images for each plate) | NR | 3000 |
| Kong and Tan (2012) | Not at all controlled (e.g., Real-world with some objects on table AND background noise) | Both standardized and non- standardized | Single and multiple food item images | Somewhat controlled (e.g., arranged, but with some item overlap/occlusion) | Multiple | NR | 3 | NR | NR |
| Lee, et al. (2012) | Highly controlled (e.g., Laboratory with grid-paper) | Standardized (e.g., chain restaurant, cafeteria, packaged foods) | Multiple separate food items | Highly controlled (e.g., laid out with clear separation between multiple items) | 19 unique foods | NR | NR | 45 | NA |
| Chen, et al. (2013) | Mostly controlled (e.g., Real-world with clear table and solid background) | Other: Food replicas of known volumes (see Fig. 6) | Single and multiple food item images | Highly controlled (e.g., laid out with clear separation between multiple items) | 17 unique foods (e.g., bread, pork chop, peanut butter, peach) | NA | 5 | 85 (17 items with 5 images each) | NA |
| Pouladzadeh, et al. (2014) | Mostly controlled (e.g., Real-world with clear table and solid background) | Non-standardized (e.g., home-prepared, non-chain restaurant, buffet) | Single and multiple food item images | Somewhat controlled (e.g., arranged, but with some item overlap/occlusion) | Multiple | more than 300 images for each food portion | 27 images for each plate of food in various conditions | over 3000 | NA |
| Siswantoro, et al. (2014) | Highly controlled (e.g., Laboratory with grid-paper) | Other: food product sample (whole fruits) | Single food | Highly controlled (e.g., laid out with clear separation between multiple items) | 1 type | 50 apples, 50 mangoes, 50 tomatoes; 2 balls (i.e., non-food item) | 5 | ~750 (5 images for each of 150 samples of food products) | NA |
| Anthimopoulos, et al. (2015) | Other: Web data set: Not at all controlled (e.g., Real-world with some objects on table AND background noise); Inselspital data set: Mostly controlled (e.g., Real-world with clear table and solid background) | Non-standardized (e.g., home-prepared, non-chain restaurant, buffet) | Multiple separate food items | Web data set: Somewhat controlled (e.g., arranged, but with some item overlap/occlusion); Inselspital data set: Mostly controlled (e.g., arranged, but items are close together on a plate/tray) | 9 broad food classes | Unclear. Inselspital data set: 248 multi-food served meals; Newly captured image data set: 24 dishes | 6 (newly captured): For each of 24 dishes, "3 different pairs of images were used and for each pair the system ran 4 times (12 estimates per dish or 288 in total)." | 24 dishes x 3 pairs of images for each = 144 | Food recognition: 3800  All the system modules: 1620 |
| Huang, et al. (2015) | Highly controlled (e.g., Laboratory with grid-paper) | Other: Fruit only | Single food | Highly controlled (e.g., laid out with clear separation between multiple items) | 1 type | Fruit Database: 10 fruits; Newly captured: 3 fruits | The database consisted of six photos of three different fruits: three apples, one peach, and two tomatoes. | 18 | To train the SVM classifier: 600 |
| Rhyner, et al. (2016)^e^ | Mostly controlled (e.g., Real-world with clear table and solid background) | Standardized (e.g., chain restaurant, cafeteria, packaged foods) | Multiple separate food items | Somewhat controlled (e.g., arranged, but with some item overlap/occlusion) | 3 types: protein, carbohydrate, and vegetables/salads | NR | 2 | 114 | NA |
| Dehais, et al. (2017) | Somewhat controlled (e.g., Real-world with some objects on table OR background noise) | Non-standardized (e.g., home-prepared, non-chain restaurant, buffet) | Multiple separate food items | Somewhat controlled (e.g., arranged, but with some item overlap/occlusion) | Multiple | NR | 6+ (3 pairs at 45 degrees from the vertical in the Meals-45 dataset, plus more images at ~7 different degrees in the Angles-13 dataset) |  | Volume estimation (food type): 270  Volume estimation (relative shooting angle on the results: NR  Volume estimation (quantify the effect of changing plates on the result, as well as to test the method on): 108  Volume estimation (to stress the system even further and test its versatility and generalization capabilities): 84 |
| Hassannejad, et al. (2017) | Somewhat controlled (e.g., Real-world with some objects on table OR background noise) | Non-standardized (e.g., home-prepared, non-chain restaurant, buffet) | Multiple separate food items | Somewhat controlled (e.g., arranged, but with some item overlap/occlusion) | 2 categories | 6 breads, 4 pastas | 6 | NR | NA |
| Minija and Emmanuel (2017) | Not at all controlled (e.g., Real-world with some objects on table AND background noise) | Non-standardized (e.g., home-prepared, non-chain restaurant, buffet) | Multiple separate food items | Not at all controlled (e.g., not arranged and/or much item overlap/occlusion) | Various food items such as brinjal, carrot, orange, cherries, French fries, juice, and burger are included. | NR | NA |  | Training and testing the feed-forward NN classifier: 100 |
| Todd, et al. (2017) | Mostly controlled (e.g., Real-world with clear table and solid background) | Standardized (e.g., chain restaurant, cafeteria, packaged foods) | Single and multiple food item images | Mostly controlled (e.g., arranged, but items are close together on a plate/tray) | 222 food items (74 of which were fruit or vegetable items) | NR | 2 images: pre and post meals | 6762 school lunch trays, pre and post images | NA |
| Ege and Yanai (2018) | Somewhat controlled (e.g., Real-world with some objects on table OR background noise) | Non-standardized (e.g., home-prepared, non-chain restaurant, buffet) | Single and multiple food item images | Mostly controlled (e.g., arranged, but items are close together on a plate/tray) | Japanese: 15 categories; American: 21 categories | NR | NA |  | No. images used for calorie estimation: Japanese food dataset: 4877;  American food dataset: 2484 |
| Emmanuel and Minija (2018) | Not at all controlled (e.g., Real-world with some objects on table AND background noise) | Standardized (e.g., chain restaurant, cafeteria, packaged foods) | Single and multiple food item images | Somewhat controlled (e.g., arranged, but with some item overlap/occlusion) | 73 categories | NR | NA |  | 1027 |
| Lo, et al. (2018) | NR; 3D models for training | Other: Real food (testing); 3D models (training) | Single food | NR | 8: banana, orange, pear, cube, potted meat can, lemon, tuna fish can, pudding box | 4 fruits; 3 packaged foods (2 canned fish, 1 pudding box) | 20,000 images per object item | 20,000 pairs of depth images (for training) | 160,000 |
| Subhi, et al. (2018) | Mostly controlled (e.g., Real-world with clear table and solid background) | NR | Single food | Highly controlled | 4 types: burger bun, pizza slice, fried chicken thigh, brownies | NA | 2 | NR, but ~8 (4 food items with 2 images each) | NA |
| Vasiloglou, et al. (2018)^e^ | Mostly controlled (e.g., Real-world with clear table and solid background) | Standardized (e.g., chain restaurant, cafeteria, packaged foods) | Multiple separate food items | Mostly controlled (e.g., arranged, but items are close together on a plate/tray) | 15 food categories were considered (e.g., pasta, rice, potatoes, carrots, salad (i.e., leafy vegetables), and fish) | NR | 2-6 | 222 meal images | NA |
| Yang, et al. (2018) | Somewhat controlled (e.g., Real-world with some objects on table OR background noise) | Food replicas | Single food | Somewhat controlled (e.g., arranged, but with some item overlap/occlusion) | NR; 15 food models used for testing; 14 foods, beverages, and non-food objects (different than those for testing) used for training | NR | NR | NR | Compare the effect of training: fifteen food models tested in both Studies 1 and 2;  Evaluate food volume estimation:  foods, beverages and non-food objects utilized for generating forty-five training images indifferent containers for Study 2. |
| Anzawa, et al. (2019) | Somewhat controlled (e.g., Real-world with some objects on table OR background noise) | Non-standardized (e.g., home-prepared, non-chain restaurant, buffet) | Single and multiple food item images | Somewhat controlled (e.g., arranged, but with some item overlap/occlusion) | 7 categories | NR; About 50 items (dishes and foods); JISS-22: 22 meal-data (e.g., 8 breakfasts, 7 lunches, and 7 dinners); about 50 items (dishes and foods) at each meal. | 3 photos for each item in JISS-22 (Fig. 3) |  | Training the detector and classifier: 195  Fine-tune the dish area detector: 304  Training the detector and classifier: 450,066  Training the classifier: 234,500 |
| Ege and Yanai (2019) | Somewhat controlled (e.g., Real-world with some objects on table OR background noise) | Non-standardized (e.g., home-prepared, non-chain restaurant, buffet) | Single and multiple food item images | Somewhat controlled (e.g., arranged, but with some item overlap/occlusion) | UEC Food-100: 100 categories; Calorie-annotated data set: 50 categories | UEC Food-100: >100 single dish images for each of 100 categories; Calorie-annotated data set: images (quantity NR) from 15 food categories were used for testing | NA | The 30 single-dish food photos and 50 multiple-dish food photos composed of 25 two-dish photos and  25 three-dish photos are taken by a camera. | Dish detection and food calorie estimation: 12,740  Extend dataset: 7,687 |
| Fang, et al. (2019) | Somewhat controlled (e.g., Real-world with some objects on table OR background noise) | Standardized (e.g., chain restaurant, cafeteria, packaged foods) | Single and multiple food item images | Somewhat controlled (e.g., arranged, but with some item overlap/occlusion) | Categories NR; ~50 unique items (see Table 1) | NR | NR |  | Pre-training: NR  Training the GAN: 202 food images for training and ~2,095 paired images to train and test cGAN |
| Makhsous, et al. (2019) | Not clear | Both standardized and non- standardized | Single and multiple food item images | NR | Multiple | NR | The algorithm extracts the six most stable frames from the video captured by the user | 360 degree video (vs. image); over 180 frames; specific frames are chosen automatically by the algorithm using the accelerometer data to find the most stable frames. | NA |
| Situju, et al. (2019) | Mostly controlled (e.g., Real-world with clear table and solid background) | Non-standardized (e.g., home-prepared, non-chain restaurant, buffet) | Single and multiple food item images | Somewhat controlled (e.g., arranged, but with some item overlap/occlusion) | 14 categories | Main task dataset: 105 to 565 per category (see Table 1); Category annotated dataset for fine-tuning: 536-3,000 images per category (see Table 2) | NR |  | Calorie and salinity: 3051  Food category classification: 28,359  Pre-training the CNN: 50,000,000 |
| Herzig, et al. (2020) | Mostly controlled (e.g., Real-world with clear table and solid background) | Standardized (e.g., chain restaurant, cafeteria, packaged foods) | Multiple separate food items | Somewhat controlled (e.g., arranged, but with some item overlap/occlusion) | 48 test meals from 128 food items | 48 meals with 128 food items | NA | 48 test meals with 128 food items | NA |
| Jiji and Rajesh (2020) | Highly controlled (e.g., Laboratory with grid-paper) | Non-standardized (e.g., home-prepared, non-chain restaurant, buffet) | Single food | Highly controlled (e.g., laid out with clear separation between multiple items) | 30 food items; 20 pictures of each food item (include vegetables, fruits, dairy and grain) | NR | 20 | 600 images (30 food items each with 20 photos) | NA |
| Lo, et al. (2020) | Highly controlled (e.g., Laboratory with grid-paper) | Other: Training dataset: 3D models of foods; Real-world experiment: 3-D models of real food items | Single food | Highly controlled (e.g., laid out with clear separation between multiple items) | Training dataset: 4 (KC's estimation: fruit, sandwiches, baked goods, grains); Real-world experiment: 4 (KC's estimation: fruit, sandwich, baked goods, breads); training loss and training accuracy are calculated based on the training dataset with 8 categories; testing loss and testing accuracy are calculated based on the testing dataset with 10 categories | Each category has 20 food models with different shape geometries and portion sizes. Linear interpolation has been used to generate 4 k food models (each category consists of 400 food models). training dataset: combination of food types (e.g., fruits, baked goods; real-world experiment: combination of food types as above | Training dataset: 300 images per item (8 items); Real-world experiment: NR - states that "videos (six trials for each food item) are captured" | NR | Training and testing: 2400 |
| Lu and Stathopoulou, et al. (2020) | Other: MADiMa: Somewhat controlled (e.g., Real-world with some objects on table OR background noise); Fast food: Mostly controlled (e.g., Real-world with clear table and solid background) | Real food, Other: MADiMa: non-standardized; Fast food: standardized | Single and multiple food item images | Somewhat controlled (e.g., arranged, but with some item overlap/occlusion) | MADiMa: 64 fine-grained food types; Fast food: 14 food types; Table A2; 18 categories (e.g., bread, pasta, potatoes, pulses/legumes/rice, fish and seafood) | NR; MADiMa: 234 food items; Fast food: 20 meals with 1-3 food items each | NR; MADiMa: Each meal contains images captured from different viewing angles (between 30 to 90 degrees with the table plane) and distance (40 - 60 cm to the table) using multiple sensors; Fast food: two-view images and stereo image pairs for each meal; goFOODTM requires an input of two meal images or a short video, must be captured from two different angles | See "Fast food" database | Evaluation: 234 food items and 64 fine-grained food types  performance of the full pipeline of the dietary  assessment: 20 meals and includes 14 different food types. |
| Makhsous, et al. (2020) | Not at all controlled (e.g., Real-world with some objects on table AND background noise) | Other: initial test: Scans of geometric shapes; Second round of testing: model food items (plastic); Third round of testing: "The final round of testing involved scanning multifood plates for measuring the volume of prepared plates of food, simulating the actual measurement of a plate with complex food items by a user."; The experiment involved standardized food from the hospital cafeteria | Single and multiple food item images | Various arrangements including food items in-contact and separated | Round 1 of testing involved 2 geometric shapes only (no food); Round 2 of testing included 4 food replicas from 2-3 types (two fruits, 1 vegetable, 1 bread); Round 3 of testing - NR; For the real-life usage experiment, images were of 3 different food plates, but types of food analyzed NR | NR | NR | Unclear, but at least 12 (there were 12 participants) | NA |
| Mohideen Pillai and Kother Mohideen (2020) | Somewhat controlled (e.g., Real-world with some objects on table OR background noise) | Standardized (e.g., chain restaurant, cafeteria, packaged foods) | Single and multiple food item images | Mostly controlled (e.g., arranged, but items are close together on a plate/tray) | "30 unique food and natural product classifications" | NR | Not clear: ~9 ("3 photos of various edges have been taken for each plate in 3 different lighting areas") or >100 (“over 100 images in each segment with different food products in Figure 6" Fig. 6 shows 23 food items; about half of the items show >100 images) | Unclear, but conclusion mentions 3000 (p. 1843) | NA |
| Shermila and Milton (2020) | Not clear | Standardized (e.g., chain restaurant, cafeteria, packaged foods) | Single food | NR | 1 type (protein drinks) | 9 health drink powders (six food items are milk powders which are used for children under 3 years and the remaining 3 food items are health drink powders) | 11 | 990 | NA |
| Chotwanvirat, et al. (2021) | Highly controlled (e.g., Laboratory with grid-paper) | Non-standardized (e.g., home-prepared, non-chain restaurant, buffet) | Single and multiple food item images | Mostly controlled (e.g., arranged, but items are close together on a plate/tray) | 175 food item classes | NR | Unclear: "Three Android smartphones were attached to three camera tripods to take photos from three different angles (30, 60, and 90 from the tabletop). While the rotating plate was operating, burst shots were taken to capture pictures simultaneously, resulting in a series of multi-angle food images." | 75,232 in Thai food dataset; 300 images of ready-to-eat local vendor's food for validation testing; 20 images were randomly selected from the validated dataset (containing 48 food items) used to compare dietitians' and authors' AI systems to estimate portion sizes | NA |
| Kumar, et al. (2021) | Highly controlled (e.g., Laboratory with grid-paper) | Other: Fruits, bread, and pizza (type NR) | Single food | Highly controlled (e.g., laid out with clear separation between multiple items) | 6 food classes: apple, banana, bread, guava, pizza, and pomegranate | 4 fruits, 1 bread, 1 pizza | 3 or more | NR | NR |
| Lu, et al. (2021) | Somewhat controlled (e.g., Real-world with some objects on table OR background noise) | Standardized (e.g., chain restaurant, cafeteria, packaged foods) | Multiple combined food items | Mostly controlled (e.g., arranged, but items are close together on a plate/tray) | 521 fine-grained food categories | 521 fine-grained categories belonging to 139 main courses, 86 side dishes, 42 vegetables, 46 soups, 32 salads, 96 sauces, and 80 desserts | 3 or more (before, during, and after consumption) | 660 RGB-D image pairs | Design the algorithms: 660 RGB-D image pairs  Network Architecture: NR |
| Ma, et al. (2021) | Mostly controlled (e.g., Real-world with clear table and solid background) | Non-standardized (e.g., home-prepared, non-chain restaurant, buffet) | Other: Other: single foods and Multiple combined food items (e.g., mixed dishes like stir fry) | Mostly controlled (e.g., arranged, but items are close together on a plate/tray) | 4 (staple, meat, seafood, veggie) | It varied from 5 items (seafood) to 54 items (veggie) | Unclear whether different angles of the same plate were taken; but there were approximately 100 images for each item (e.g., "stir fried cauliflower") | NA | 1.2 million training images, 50,000 validation images, and 150,000 testing images  ChinaFood-100:10074 |
| Papathanail, et al. (2021) | Mostly controlled (e.g., Real-world with clear table and solid background) | Standardized (e.g., chain restaurant, cafeteria, packaged foods) | Multiple separate food items (e.g., plate with burger and fries) | Somewhat controlled (e.g., arranged, but with some item overlap/occlusion) | six food types (soup, meat/fish, side dish, sauce, vegetables/salad, dessert) | NR | 2 images (before consumption and after) | 332 (166 meals x 2 images) | 534 |
| Yang, et al. (2021) | Mostly controlled (e.g., Real-world with clear table and solid background) | Other: Virtual food databases were generated to train and test the model | Single food | Highly controlled (e.g., laid out with clear separation between multiple items) | Multiple | NR | single-view image to estimate food volume; in the Ideal real food dataset, has multiple images; in virtual food datasets, seems multiple images representing different volumes of the same virtual foods were generated. | Ideal real food dataset: 1500 images; General real food dataset: 416 images | VFDL-15 (generated food dataset utilizing 15 classes and large volumes: 400 mL to 3400 mL) Evaluation 14892  VFDS-15 (generated food dataset utilizing 15 classes and small volumes: 200 mL to 1700 mL) Evaluation: 13694 |
| Yuan, et al. (2021) | Highly controlled (e.g., Laboratory with grid-paper) | Other: computer synthesized 3D objects were used in the first experiment to evaluate the performance of the VD meter | Single and multiple food item images | Highly controlled (e.g., laid out with clear separation between multiple items) | 6 foods (e.g., bread, burger) purchased from cafeteria and food stores, were used to study the real-world performance of the VD meter | NR | images were obtained by the three cameras of the VD meter for each food measurement | 64 images were taken for each camera, and total 64*3=192 images were obtained by the three cameras of the VD meter for each food measurement | NA |
| Dai, et al. (2022) | Not at all controlled (e.g., Real-world with some objects on table AND background noise) | Standardized (e.g., chain restaurant, cafeteria, packaged foods) | Multiple separate food items (e.g., plate with burger and fries) | Not at all controlled (e.g., not arranged and/or much item overlap/occlusion) | Gimbap | Unknown | NR | NA | Korea Food Image database: 1000 |
| Kadam, et al. (2022) | Not at all controlled (e.g., Real-world with some objects on table AND background noise) | Both non-standardized and standardized | Other: All: Single food, multiple separate food items, and multiple combined food items | Somewhat controlled (e.g., arranged, but with some item overlap/occlusion) | 7 classes of Indian breakfast foods (oranges, bread, Dosa, upma, samosa, Vada-paav, meduvada) from Fig. 9, plus 1 class of bowls | NR | NR | researchers took images for the bowl and coin dataset, which had approx. 70 images of bowl and reference object coins with top and side view (Fig. 4), and of bowls alone (stainless steel, glass, ceramic, plastic); that overall dataset had approx. 100 images | Regular shaped food: ~ 100  Irregular shaped food dataset ~120  Amorphous food item dataset: NR  Bowl and coin dataset: ~100 |
| Li and Yang (2022) | Unclear | Non-standardized (e.g., home-prepared, non-chain restaurant, buffet) | Unclear | Mostly controlled (e.g., arranged, but items are close together on a plate/tray) | 34 types | Unclear | Unclear | NR | CFNet-34 = ChineseFoodNet + Images newly acquired  Unknown total (23 kinds of food + 11 = 34 kinds; in the tests, they used 60 images with one kind of foods; 60 images with two kinds of foods. . . up to nine kinds of food in the image; this suggests at least 2700 images) |
| Ma, et al. (2022) | Mixed: "Mostly controlled" and "Not at all controlled" | Non-standardized (e.g., home-prepared, non-chain restaurant, buffet) | Single and multiple food item images | Mixed: "Not at all controlled" and "Mostly controlled" (see Fig 5) | 18 main groups; 109 classes | 100 images per class -- assuming all of these had distinct items, but there could have been multiple angles of the same item | NR | 10921 | Internet websites  100 per class, with 109 classes = approx. 10,900 images; clustered into 18 groups according to food classification  100 images each for 100 food classes = 10,000  1000 images of each of 101 food classes = 101,000 |
| Minija and Emmanuel (2022) | Somewhat controlled (e.g., Real-world with some objects on table OR background noise) | Other: Standardized (cafeteria) | Multiple separate food items (e.g., plate with burger and fries) | Mostly controlled (e.g., arranged, but items are close together on a plate/tray) | 73 | NR | NR | NA | UNIMIB2016 dataset  1,027 |
| Pfisterer, et al. (2022) | Mostly controlled (e.g., Real-world with clear table and solid background) | Standardized (e.g., chain restaurant, cafeteria, packaged foods) | Multiple separate food items (e.g., plate with burger and fries) | Somewhat controlled (e.g., arranged, but with some item overlap/occlusion) | 36 (9 regular texture and 27 modified texture) | 6 grains, 14 vegetables and fruits, 9 proteins, and 7 mixed foods | Regular dataset: 9 regular texture foods across three meals; up to three meal items and imaged at 25% increments relative to full portion. 125 unique plates per meal, 375 unique plates across meals.  Modified texture foods dataset: 64 images across 93 classes of modified texture food samples; 47 unique foods; samples were imaged at different simulated intake levels | NA | UNIMIB2016: pre-labelled food dataset: 1027 tray images, 73 categories  Regular foods dataset: 375  Modified texture foods dataset: 314 |
| Prakash, et al. (2022) | Unclear | Fruits | Single food | Unclear | 1, Fruits | NR | NR | NA | FRUITS  500 images in the total dataset; "75 samples" were used; they cite "Medus et al. 2021" but did not include the full reference |
| Sasaki, et al. (2022) | Mostly controlled (e.g., Real-world with clear table and solid background) | Standardized (e.g., chain restaurant, cafeteria, packaged foods) | Other: Multiple items | Somewhat controlled (e.g., arranged, but with some item overlap/occlusion) | 15 (cereals, potatoes, pulses, nuts, vegetables, green & yellow vegetables, fruits, mushrooms, seaweed, fish & shellfish, meats, eggs, dairy products, confectioneries, alcoholic beverages | NR | Unclear, but paper states "Four staff took a picture of each meal" (See Fig. 2), and "For every sample meal, 4 research staff registered data X and Y. We calculated the means of the 4 entries of data X and Y and compared them with data G as the gold standard." | NR, but seems like 480 images (120 meals x 4 images each) | "CALO mama app database, which has food images and nutrient information including fresh food,  self-made meals, ready meals, and commercial products"  NR: approx. 150,000 foods |
| Tagi, et al. (2022) | Mostly controlled (e.g., Real-world with clear table and solid background) | Standardized (e.g., chain restaurant, cafeteria, packaged foods) | Multiple separate food items (e.g., plate with burger and fries) | Highly controlled (e.g., laid out with clear separation between multiple items) | 5 types of food and liquids food: staple food, side dishes 1, side dishes 2, packaged beverages, and seasonings | 1 staple food; 4 slide dishes1; 6 slide dishes2, 5 package beverage; 1 seasoning | 6 images per item; 432 total images for thin rice gruel; 72 total images for fermented milk; 72 total images for peach juice | A total of 576 images of liquid food (432 images of thin rice gruel, 72 of fermented milk, and 72 of peach juice) | NR |
| UlHaque, et al. (2022) | Somewhat controlled (e.g., Real-world with some objects on table OR background noise) | Standardized (e.g., chain restaurant, cafeteria, packaged foods) | Multiple donuts | Other: Somewhat controlled (e.g., arranged, but with some item overlap/occlusion) | 5 | 1000 images of each apple, banana, etc. | Each category of food images was preserved along with the top and side view of the food items. | NA | Food-101: 5000 - No. in database 2  Fruit-360: 5000 - No. in database 1 |
| Zhang, et al. (2022) | Mostly controlled (e.g., Real-world with clear table and solid background) | Unclear, but seems to be standardized (maybe chain restaurant) | Multiple separate food items (e.g., plate with burger and fries) | Highly controlled (e.g., laid out with clear separation between multiple items) | 10 types of foods for Aliyun Cloud Food Recognition database;  51 common dishes for mobile phone database | Aliyun Cloud Food Recognition database has 270 meat, 188 vegetable, 145 meat and vegetable, 248 staple food, 134 fruit, 110 drink, 113 snack, 115 soup, 66 nut, 66 set meal; Mobile phone database has 1 each for 51 common food | 12 images for each common food in Mobile phone database | 1455 for evaluating Aliyun API  612 for training the weight-area model and 612 for evaluating the area-weight model. | NA |
| Nadeem, et al. (2023) | Somewhat controlled (e.g., Real-world with some objects on table OR background noise) | Non-standardized (e.g., home-prepared, non-chain restaurant, buffet) | Single food | Somewhat controlled (e.g., arranged, but with some item overlap/occlusion) | 14 "classes": apple, sour cream, chicken curry, branded soda cans (2), coin, chicken, sour cream, branded sandwiches (4 types), orange, pizza | Unclear: likely varied (4 sandwiches, 2 fruits, etc.) | NR | over 16,000 images | NA |
| Shao, et al. (2023) | Mostly controlled (e.g., Real-world with clear table and solid background) | Unclear; possibly both. | Seems to be multiple separate food items and multiple combined food items | Not at all controlled (e.g., not arranged and/or much item overlap/occlusion) | Nutrition5k database has more than 250 food categories; Food2k contains 2,000 categories | NR | Unclear, but for Nutrition5k, "the RGB-D set was constructed following the principle of incrementality, i.e., adding one at a time to the plates and scanning after each food item was added." | NA | Nutrition5k dataset: 2,960 pairs RGB-D images  Food2k dataset: >1 million images |
| Zheng, et al. (2023) | Highly controlled (e.g., Laboratory with grid-paper) | Standardized (e.g., chain restaurant, cafeteria, packaged foods) | Single food | Highly controlled (e.g., laid out with clear separation between multiple items) | 7: egg, orange, chicken leg, bread, grapefruit, cake, peach (all food replicas) | NR | 2 | Unclear; Possibly 7, with one image of each of 7 food replicas (p. 27) | NA |

NR, not reported; NA, not applicable; 3D, three dimensional; KC, Kelly Cara second author.

^a^ Image setting: Highly controlled (e.g., Laboratory with grid-paper); Mostly controlled (e.g., Real-world with clear table and solid background); Somewhat controlled (e.g., Real-world with consistently good lighting); Not at all controlled (e.g., Real-world with some objects on table AND background noise).

^b^ Type of foods: “standardized” includes foods from chain restaurants, cafeterias, or packaged foods (foods where nutrient content has been measured and reported); “non-standardized” includes home-prepared, non-chain restaurant, and buffet foods (foods where nutrient content has not been measured or is not readily available).

^c^ Number of foods per image: Multiple combined food items (e.g., mixed dishes like stir fry); Multiple separate food items (e.g., plate with burger and fries); Single food (e.g., single apple with no other foods in the image); Single and multiple food item images (e.g., a combination of the above options).

^d^ Whether and how foods were arranged: Highly controlled (e.g., laid out with clear separation between multiple items); Mostly controlled (e.g., arranged, but items are close together on a plate/tray); Somewhat controlled (e.g., arranged, but with some item overlap/occlusion); Not at all controlled (e.g., not arranged and/or much item overlap/occlusion).

^e^ Rhyner, et al.^51^ and Vasiloglou, et al.^12^ report on different studies using the same tool.

**eTable 6.** Image-based dietary assessment methods and AI approaches used in included papers

| **Author (year)** | **Country of authors’ affiliations; Funding** | **System name or description** | **Tool used for image acquisition** | **Food image datasets used** | **System requirements (e.g., fiducial marker)** | **Food detection and classification approach** | **Volume/weight estimation approach** | **Energy or nutrient estimation approach; source of nutrient data** |
| --- | --- | --- | --- | --- | --- | --- | --- | --- |
| Zhu, et al. (2010) | USA; Government | Novel mobile telephone food record | Mobile devices (e.g., mobile telephone or PDA-like device); Apple iPhone or Nokia N810 Internet Tablet | Database of before/after meal images from the Department of Foods and Nutrition at Purdue University | Calibrated fiducial marker: color checkerboard for color imbalance in camera | Segmentation: connected component analysis, active contours, and normalized cuts  Classification: statistical pattern recognition techniques (i.e., SVM or support vector machine) | Spherical and prismatic approximation models with visual refinement  Camera parameter estimation through camera calibration using a fiducial marker as a reference for the scale and pose of the food item | From volume estimation, density (*g/in^3^*) is derived from FNDDS, then mass is calculated and converted to nutrients; FNDDS |
| Kong and Tan (2012) | USA; NR | DietCam | Smart phone; iPhone | Global database: of popular food types; collected manually from the developers’ input and Stockfood – the food image agency. (<http://www.stockfood.com>.)  Small personal database: developed as a cache in the image manager | Online chessboard pattern to calibrate the camera’s intrinsic parameters (needed one time only)  Fiducial marker, credit card  A classifier that classifies food items based on the number of matched vectors | Identification: optical character recognition (OCR) techniques or user inputs  Segmentation: visual features  Classification: Bayes decision theory based probabilistic food classification algorithm built on feature matching based object recognition; vocabulary tree data structure to make image matching scalable; SIFT features are clustered into visual words with an efficient hierarchical k-means clustering algorithm | 3-D reconstruction: Predefined shape models for regular shaped food items; 3D models reconstructed directly from points. For regular shaped foods, volume estimation model is prebuilt for the food type.  3-D volume estimation: Coordinates of all the points are calculated. Mass point is estimated by averaging all coordinates; Mass point is connected to each 3D point, forming a group of tetrahedrons. Volume of the food item is the sum of the volume of every single tetrahedron. | Calorie density is used to estimate energy roughly; number of calories = volume x mass density x calorie density; FNDDS |
| Lee, et al. (2012) | USA; Government | Portion weights automated from mobile telephones device | Other: HTC p4351 mobile telephones (HTCAmerica, Bellevue, WA) running Windows Mobile 6.0 (Microsoft, Redmond, WA) | None | Fiducial marker | Classification: support vector machine (SVM) | For portion volumes: Camera parameter estimation and model reconstruction  For images acquired at a shallow (oblique) angle, surface contact information is manually supplied, or automatic computation by symmetry cues is used  Feature point detection algorithm and shape analysis techniques: medial axis and active contour | FNDDS |
| Chen, et al. (2013) | USA and China; Government | 3D Model-based measuring volume/portion size of food | Digital camera | None | The serving container for scale. Measurements performed by subject before or after the dietary study. | Base plane localization: Plane on which the base of the food is sitting with respect to the camera coordinate system  Food segmentation: Combination of adaptive thresholding approach and edge-based snake model to segment foods from the detected plate region | 3D/2D model-to-image registration scheme to fit a 3D shape model with the food. The volume of the food is estimated from the analytically calculated volume of the fitted shape model | Database NR |
| Pouladzadeh, et al. (2014) | Canada; NR | Mobile technology with special calibration technique measures calorie and nutrient components | Other: 3 different digital cameras including a smartphone camera: Canon SD1400, iphone 4, and Canon SD1300 | None | Thumb; one-time calibration | Texture segmentation: Gabor filter  Food portion identification: Support vector machine (SVM) scheme  Radial basis function (RBF) kernel to map samples into a higher dimensional space in a nonlinear manner | Superimpose a grid of squares; adjust granularity of grid, if needed (based on mobile phone capabilities) | Calorie in the photo = (Calorie from table × Mass in the photo) / (Mass from table)  Tables available from national and international health organizations (not specified) |
| Siswantoro, et al. (2014) | Malaysia, Indonesia, and Saudi Arabia; Government | Monte Carlo method for volume estimation | Digital camera | None | Fiducial marker: ten different views of calibration object for intrinsic camera parameters. For the extrinsic camera parameter estimation, calibration object was in the bottom of the measured object and was assumed to lie on plane=0 in the real-world coordinate system. |  | Volume measurement using a computer vision system for irregularly shaped food products based on Monte Carlo method with heuristic adjustment: (1) 3D bounding box construction using Monte Carlo method; (2) generate 3D random points; (3) Monte Carlo integration; (4) heuristic adjustment | NA |
| Anthimopoulos, et al. (2015) | Switzerland and USA; Government | GoCARB | Smart phone | food images downloaded from the Internet. images are characterized by high visual diversity (background, lighting conditions, angle, arbitrary serving, etc)  Inselspital data set (images from 248 multifood served meals prepared at the Inselspitals restaurants) | Web data set: no fiducial marker; Inselspital data set: fiducial marker | Plate detection: ellipse detector with edge map from Canny edge detector and edge segments in incremental random sample consensus (RANSAC)  Segmentation: Images converted to the CIELAB color space, pyramidal mean shift filtering is applied, refined using detected ellipse  Food recognition with description and classification: Color k-means algorithm; texture features (local binary pattern (LBP) histogram values); nonlinear support vector machine (SVM), with a radial basis function (RBF) kernel that assigns the segment to 1 of 9 predefined food classes. | 3D model reconstruction and volume estimation using passive stereo vision; Speeded up robust features (SURF) detected and matched between the 2 images. RANSAC model fitting is applied to the matched pairs to extract a candidate pose; image pairs are transformed so point correspondences lie on the same row, using polar rectification. | FNDDS |
| Huang, et al. (2015) | Australia; NR | Application: OpenCV | Smart phone | Unclear; To train the SVM classifier, a fruit database was collected with 10 types of fruits. Each fruit type contained 60 images. Database was randomly separated into a training and testing datasets (50 and 10 images each fruit type, respectively) For volume performance, another image dataset was obtained from a smartphone (Samsung Galaxy S2). | Fiducial marker, Credit card | Classification: Support Vector Machine (SVM)  Scale Invariant Feature Transform (SIFT); categorized by a method called Bag-of-Words (BOW); texture was quantified by the Local Binary Pattern (LBP) method | Volume: Modelling approach; light-intensity threshold to segment the item and obtain contour and central axis of symmetry; revolving the contour around the central axis, a ball-shaped model was constructed, and its volume was determined. | A fruit database (not specified) to obtain the density of the food and carbohydrate concentration |
| Rhyner, et al. (2016)^a^ | Switzerland and USA; Government and nonprofit | GoCARB; smartphone-based carbohydrate counting, employing computer vision, machine learning, and smartphone technologies | Smart phone | None | Reference card | Dish detection by extracting edges of image and applying a robust fitting paradigm; automatic recognition to segmented food item by color and texture features to support vector machine; 3D shape is reconstructed; key points matched between two images to define the orientation and location; image pixels (correspondence and disparity) provide depth for 3D model | By using the 3D model and the segmentation results, the volume of each item is calculated | The carbohydrate content is calculated by combining the volume of each food item with the nutritional information provided by the USDA Nutrient Database for Standard Reference; FNDDS |
| Dehais, et al. (2017) | Switzerland; Government | 3D reconstruction and food volume estimation using stereovision on mobile device | Smart phone | Meals-45 (45 dishes [113 distinct food items] from the restaurant facilities of the Bern University Hospital, Inselspital)  Angles-13 (a secondary dataset of the Meals-45 dataset)  Plates-18 (includes 6 meals from an international fast food retailer, served in 3 different types of plates)  Meals-14 (meals from local supermarkets and restaurants) | Fiducial marker | Extrinsic calibration: Salient point matching (SURF was used), relative pose extraction (RANSAC-based method and models are created using the five point relative pose model generator of Nister; includes local optimization [LO] and Levenberg Marquardt [LM] optimization algorithm), and scale extraction | Dense reconstruction: Stereo matching approach involving rectification of the images, stereo matching, point cloud generation, and volume extraction (using RANSAC) | Database NA |
| Hassannejad, et al. (2017) | Italy; Government | image-based modeling with stochastic object-detection method for volume estimation | Smart phone | None | Fiducial marker: checkerboard used as size/ground reference | Segmentation: Gaussian Mixture Model alongside the graph-cut algorithm. | Point-cloud to model the food; stochastic object-detection method locates checkerboard  Segmentation: customized interactive version of the graph-cut (GC) algorithm deals for pixel labeling  Speeded Up Robust Features (SURF) extracted and Binary Robust Invariant Scalable Keypoints (BRISK) description is used to find matches between image pairs | Database NA |
| Minija and Emmanuel (2017) | India; NR | Neural network classifier for image segmentation and calorie calculator | NR | Dataset: food images collected from publicly available resources | Pixels; superimposed square grid with equal number of pixels | Segmentation: feed-forward NN classifier  Classification: Training algorithm of the feedforward NN: extract image features  The testing the feedforward NN: (1). the food features fed into the feed-forward NN. (2). training algorithm is called (3). the food features of testing images are compared with the trained feature set to attain the desired classified food items. | total area and depth in image used to calculate volume | Nutrient database https://www.calorie-charts.net/ |
| Todd, et al. (2017) | USA; Government | Digital food image analysis, beta version | Digital camera | None | NR, but identification ruler and lunch tray with known dimensions may have served as referents | Recognition of food item and quantity: Digital Food Image Analysis (DFIA); Size, shape, color, and density of photographed items are analyzed through using adaptive neural networks |  | FNDDS |
| Ege and Yanai (2018) | Japan; NR | Image-based food calorie estimation via CNN | NR | Japanese Calorie-Annotated Food Photo Dataset: ~83,000 recipes from six recipe web sites that indicated professionals such as chefs provided recipe information  American Calorie-Annotated Food Photo Dataset: ~24,000 recipes data collected from All recipes web site | NA | Feature extractor: VGG16 is pre-trained with the ImageNet 1000-class dataset.  Extracted activation signals of fully connected layers (fc layers) of the VGG16 network as CNN features; 4096-dim feature vector for each food image. |  | Calorie: calculating an average value of food calories of the top k similar images; NA |
| Emmanuel and Minija (2018) | India; NR | CSW-WLIFC algorithm; Whale-Levenberg Marquardt | NR | UNIMIB2016 dataset | Tray size might be used as a reference, but this is not clear; Pixels of the image are used to determine food area | Segmentation: Cauchy, Generalized T-Student, and Wavelet kernel based Wu-and-Li Index Fuzzy clustering (CSW-WLIFC) based segmentation segments the image based on the existing WLI-FC algorithm.  Feature vectors such as color, shape, and texture are extracted from the segmented image.  Recognition: The Neural Network is trained with the Whale-Levenberg Marquardt (WLM) model to recognize each food item from the tray image. |  | Database NR |
| Lo, et al. (2018) | UK; Nonprofit | A depth camera-based dietary assessment technique | Other: Mobile phone with depth sensors or a depth camera | Yale-CMU-Berkeley object set (dataset [https://www.doc.ic.ac.uk/~ys4315/Food_dataset.zip] constructed through image rendering based on virtual 3D models of the real-life objects) | Study used digital 3D models only where dimensions were known | Segmentation and Classification: image segmentation approach (ex. Mask R-CNN) | Point cloud completion with the deep learning view synthesis is then applied to each labeled object item to perform 3D reconstruction and estimate food volume; a modified Iterative Closest Point (ICP) algorithm was developed to address the problem of misalignment of initial and synthetic point clouds | FNDDS, but calorie results NR |
| Subhi, et al. (2018) | Malaysia; NR | Stereo image approach | Wearable device (eyeglasses with two image sensors (to produce stereo images) | None | Stereo images and known camera intrinsic parameters | 1. Object displacement  2. Dimension estimation of the object  3. Object corners identification: Harris corners and an edge detection algorithm | Vision-based approach; surrounding box for irregular shapes where the maximum dimensions of the object are the dimensions of the box. | Mass and calorie estimation; For food density: International Network of Food Data Systems (INFOODS) database; FNDDS |
| Vasiloglou, et al. (2018)^a^ | Switzerland, Germany, Austria; Government | GoCARB is a novel smartphone-based approach for carbohydrate counting, employing computer vision, machine learning, and smartphone technologies | Smart phone | None | Fiducial marker: a credit card-sized reference card (8.5 x 5.5 cm) | Dish detection by extracting edges of image and applying a robust fitting paradigm; automatic recognition to segmented food item by color and texture features to support vector machine; 3D shape is reconstructed; key points matched between two images to define the orientation and location; image pixels (correspondence and disparity) provide depth for 3D model | By using the 3D model and the segmentation results, the volume of each item is calculated | The carbohydrate content is calculated by combining the volume of each food item with the nutritional information provided by the USDA Nutrient Database for Standard Reference; FNDDS |
| Yang, et al. (2018) | USA, China and Australia; Government | smartphone-based imaging system | Smart phone | Nasco Life/form Food replica | the length (or the width) of the smartphone itself is used to calibrate the imaging system |  | Food volume estimation: International Food UnitTM (IFUTM), where a virtually generated cube is used as the unit of estimation.  Using virtual reality, an estimator scales the cube up or down until the volumes of the cube and the food are visually equivalent; implemented in MATLAB with a graphical interface. | Database NA |
| Anzawa, et al. (2019) | Japan; Government | Images recognition using single photo via NN searches | NR | JISS-22 ("real data obtained from the restaurant"; <https://www.jpnsport.go.jp/jiss/>)  JISS-DET  FLD-DET: FoodLog Dataset (FLD) from FoodLog App  FLD-469: FoodLog Dataset (FLD) from FoodLog App | No | Localized dishes; hierarchical recognition; specific food classes recognition; using deep features and applying NN searches on the template and target features; take deep features, from the output of the last average pooling layer of ResNet 50 and apply L2-normalization |  | Foods in image were assumed to be one serving size, and nutrient information was calculated; Database NA |
| Ege and Yanai (2019) | Japan; Government | Multi-task CNN | Digital camera | UEC Food-100 (Japanese food photo dataset which includes multiple-dish food photos attached with bounding boxes, and a calorie-annotated food photo dataset which contains single-dish food photos with food calories)  Calorie-Annotated Food Photo Dataset (from Ege 2018; calorie-annotated recipe data collected from commercial cooking recipe sites on the web) | No | Detect dishes: Single-shot multibox Detector SSD |  | Modify the network of SSD based on VGG16 to output a food calorie value on each food bounding box. Use image-based food calorie estimation based on regression learning with CNN to detect dishes and estimate food calories simultaneously; Database NR |
| Fang, et al. (2019) | USA; Government | An End-to-End energy estimation system | NR | ImageNet  image-to-energy distribution data set (constructed from food images collected from the Food in Focus study where "Each food item and each eating occasion image were manually labeled and segmented in the data set") | Fiducial marker | Generative Adversarial Network (GAN) architecture; used a CNN based regression model; To resize the output from generative model, we used OpenCV implementation of image resize, which is based on linear interpolation | cGAN to estimate energy distribution image | FNDDS |
| Makhsous, et al. (2019) | USA; Government | 3d reconstruction; mobile structured light system | Other: Digital Dietary Recording System (DDRS): Smart phone camera fitted with a structured light system device | None | structured light system | The DDRS software consists of three main algorithms: image segmentation, automatic laser dot detection, and 3D volume calculation; manual dot selection implemented using MATLAB | Digital Dietary Recording System (DDRS) algorithm uses the structured light system, as well as the recorded video, to calculate the volume of the food. | Nutritional Data Systems for Research (NDSR), but calorie results NR |
| Situju, et al. (2019) | Japan and Indonesia; Government | Multi-task CNN | NR | Ingredient annotated dataset: food images annotated with calorie and salinity information from 6 commercial cooking recipe sites on the Web  Rakuten18: dataset with category-annotated food images for 14 categories of foods  ImageNet: a dataset for generic object recognition | No | Food ingredient estimation: Multi-task CNN; then, the Xception model, which has achieved a high recognition rate in image classification tasks using Image-Net, is used as basis for the proposed architecture. | Regression model with mean square error used to model relative and absolute error for calories and salinity | Recipe information provided by experts such as cooks and cooking researchers from published websites |
| Herzig, et al. (2020) | Switzerland; Nonprofit and government | computer vision on a depth-sensing smartphone | Smart phone | None | No | Depth map from phone's front sensors; image is partitioned into regions using a CNN structured by pixels and their correlations visual appearance of images; visible point cloud is transformed using a Delaunay triangulation; the location and orientation of the table using the RANSAC-Algorithm for an outlier-robust fitting | The segmented food items are used to cut the visible surface into partial food surfaces. Each food surface is then closed by the dish surfaces before their volume is calculated. | Swiss Food Composition Database |
| Jiji and Rajesh (2020) | India; NR | Vision-Based Measurement (VBM) | Smart phone | None | No | the VBM method: pre-processing; Food Portion Segmentation; using Color & Texture, K-mean Clustering, and Graph Cut-Based Segmentation; Feature Extraction Stage; Food Recognition stage based on features using different classification methods such as a Support Vector Machine (SVM), Neural Network, and Deep Learning |  | Calorie information from national and international health organizations was multiplied by volume; Tables from national and international health organizations (not specified) |
| Lo, et al. (2020) | UK; Nonprofit | UNet, VNet, | Other: depth camera | AutoCAD used to build a new food dataset (10 common food categories including burger, fried rice, pizza, etc.); each category has 20 food models with different shape geometries and portion size; Linear interpolation used to generate 4 k food models | Training Dataset: Rubik’s cube; Real-world experiment: NR |  | Two network architectures UNet and VNet, complete the partial point clouds and estimate the actual volume (cm3) of 3-D models; novel data augmentation method to enlarge the dataset of 3-D models using linear latent interpolation to ease the network convergence; point cloud reprocessing techniques are developed to facilitate volume estimation. | Database NA |
| Lu and Stathopoulou, et al. (2020) | Switzerland and USA; Nonprofit | GoFOOD | Smart phone | MADiMa database: contains images of 80 central-European style meals captured by monocular cameras  Fast food database: new database that contains both two view images and stereo image pairs of each meal using food from the international fast food chain, McDonald  Training dataset: "images collected from the Internet, from publicly available databases and the internally captured meal images" | for different-view images from a single-camera smartphone; No for stereo-image pair using a smartphone with two rear cameras; other: a reference object of known size must be placed next to the food when taking photos | Segmentation: Mask R-CNN; for the semi-automatic food segmentation, the traditional region growing and merging algorithm is applied.  Recognition: Inception V3; deep neural networks are used to process the two images and implement food detection, segmentation, and recognition | a 3D reconstruction algorithm estimates the food's volume | Nutritionix Database: calories and macronutrient content of each food category (per 100 ml)  MADiMa database: USDA and/or Swiss  Fast food database: official website of McDonalds |
| Makhsous, et al. (2020) | USA; Government | DietSensor | The scanner hardware consists of a smartphone, an infrared (IR) projector, and a camera module to record depth information; Commercial Off The Shelf (COTS) depth sensor called Structure Sensor (made by Occipital) | None | No | Segmentation of the plate, as well as the individual food items, were done within Meshmixer. | generalizable hole-filling algorithm, which applies during post-processing; Post-processing of the 3D models is done using the VCA algorithm; post-processing used Autodesk Meshmixer; After filling the generated holes, Meshmixer calculates the volume of the object; For holes, boundary vertices are found, and the advanced front mesh (AFM) technique is used to fill the hole. | Nutritional database provided by the Harborview Medical Center kitchen; Nutrition Coordination Center (NCC); SelfNutritionData; FatSecret; but nutrient results NR |
| Mohideen Pillai and Kother Mohideen (2020) | India; NR | Neural Deep learning Network in android applications | Digital camera | None | Thumb | Segmentation and feature extraction (shape, size, and color): cloud SVM and deep neural network technique |  | Various databases and healthy facts tables such as Santé Canada (limited sample of a shared Food Reality table); tables from international and regional welfare organizations (not specified) |
| Shermila and Milton (2020) | India; NR | deep learning CNN; image features with linear regression using SVM | Smart phone | None | No | Deep convolutional neural network is formed with (1) image input layer, (2) convolutional layer, (3) normalization layer, (4) linear rectification layer, (5) pooling layer, (6) fully connected layer and (7) regression layer. |  | Nutrition facts from the selected health drink manufacturers (assumed) |
| Chotwanvirat, et al. (2021) | Thailand; Government | not specified | Smart phone | None | Other: A spoon was used in some, but not all images | YOLOv4-tiny for object detection. Transfer learning was used to retrain a Microsoft Common Object in Context (MS COCO) pre-trained model. Darknet for training. GrabCut was used for image segmentation. | The system consisted of three independent algorithms, the convolutional neural network (CNN)-based object detector, segmentation unit, and neural network regression-based weight estimation unit. | Other: Weight to nutrients; INMUCal-Nutrients software database; Thai Food Exchange List or official Carbohydrate Counting Guidebook |
| Kumar, et al. (2021) | India and South Korea; Government | multilayer perceptron model | NR | None | No | feature extraction (SIFT method, gabor filter, color histogram);classification: multilayer perceptron (MLP) and SVM classification; Gabor filter | SVM vectors with features are used to calculate length, width, height and depth, which is used to calculate area and then volume, which is multiplied by food value | Database NR |
| Lu, et al. (2021) | Switzerland; Nonprofit | multi-task contextual network; few-shot learning-based classifier; 3D construction | Other: Intel RealSense RGBD sensor | Nutrient Intake Assessment Database (NIAD): "RGB-D image pairs of 322 real world meals, including 1281 food items associated to 521 food categories in total.”  ImageNet | Segmented plates and classified the plate types | Segmentation: a Multi-Task Contextual Network (MTCNet), with pyramid architecture for feature encoding and Contextual layer (CTLayer) to provide contextual information between foods and serving plates;  Recognition: few-shot learning-based classifier; RANSAC algorithm; the training data is augmented using a Generative Adversarial Networks (GAN)-based approach | Volume: a 3D surface construction algorithm | Nutrient intake calculator that links the consumed volume; Central kitchen of Bern University Hospital |
| Ma, et al. (2021) | China and USA; Government | VGG with larger kernel-sized filters; ResNet-50 or ResNet-152; Wide ResNet-50; Inception V3 | NA | ImageNet-1000; ChinaFood-100; images downloaded from <https://image.baidu.com/>; ChinaMarketFood-109; UECFood-256; Food-101 | Not reported | Food classification: deep CNN, used the pre-trained architectures (e.g. Inception V3), which will take images as input and classify it into 100 categories, each associated with a probability. | Nutrition estimation: derived from the classification results given each food category has a pre-defined and fixed nutrient configuration (e.g. calories, protein, fat, carb, vitamin, micronutrients). | Other: Portion; Nutrient facts for each category from from Dietary Guidelines for Chinese (http://www.fao.org/nutrition/education/food-dietar y-guidelines/regions/countries/China/en), the amount of 23 common nutrients (see in Table S1) for each class was annotated as a ground truth value. |
| Papathanail, et al. (2021) | Switzerland; No external funding | ResNet50 architecture (ResNet + PSPNet) or a simple encoder with five stacks of convolutional layers in a row (Encoder + PSPNet) | Other: Standardised mount with RGB-D camera, connected to a laptop. | Dataset from previous study (ref 24) | Technology measured depth data, so a fiducial marker was not needed. | For the food segmentation: Pyramid Scene Parsing Network (PSPNet, a CNN) to extract the feature map, using either ResNet50 or a simple encoder with five stacks of convolutional layers in a row. Then a pyramid parsing module was applied. | To estimate the volume consumed and the macronutrient intake:  The depth image generated a 3D point cloud, used to model food and plate surfaces using segmentation masks. Food volume was estimated by subtracting the plate surface. Percent consumed was calculated from volume before and after meals. Nutritional intake was determined by multiplying the consumed percentage with dish nutritional data from the kitchen database | Volume to nutrients; nutritional information of the individual menus was retrieved from the kitchen database (SANALOGIC Solutions GmbH) |
| Yang, et al. (2021) | China and USA; Government | Modified MobileNetV2 model | Other: Ideal real food dataset: stationary camera; General real food dataset: personal mobile phones (brands unrestricted) | VFDL-15 (generated food dataset utilizing 15 classes and large volumes: 400 mL to 3400 mL)  VFDS-15 (generated food dataset utilizing 15 classes and small volumes: 200 mL to 1700 mL) | No | Image classification network outputs a vector of the probability values with respect to a pre-selected set of reference class based on the inverted residual block.  Trained our deep neural network using the standard Stochastic Gradient Descent(SGD) algorithm. | Food volume directly from a single-view 2D RGB images  Food volume is estimated by an inner product between the probability vector and a volume vector consisting of the volumes of reference classes | Database NA |
| Yuan, et al. (2021) | China and USA; Government and Nonprofit | Volume of Density (VD) meter | Digital camera | None | No |  | Volume estimation methods, including the convex-hull method, the slice-based method and the electric field methods; point cloud with two methods: (1) a simple sliced point cloud method and (2) a robust estimation method; SURF, Harris and FAST with combined features to extract feature points from images. | Database NA |
| Dai, et al. (2022) | Korea; Government | Mask R-CNN with Inception_v2 with RPN (Region Proposal Networks) classification network | NA | Korea Food Image database | Unclear | Food recognition: Used a pre-trained model using the TensorFlow's object detection API for Gimbap recognition. Mask R-CNN model then returns the bounding box and the mask for each detected Gimbap. They created a labeling approach for the Gimbap image datasets and fine-tuned Mask R-CNN architecture for efficient segmentation of Gimbap Korean food. | Calorie estimation approach is proposed, combining the calibration result and the Gimbap instance segmentation result | Portion to nutrients (e.g., 4 fries); Unknown |
| Kadam, et al. (2022) | India and USA; NR | Mask-RCNN | Smart phone | Regular shaped food -images from internet: oranges and bread  Irregular shaped food dataset - 30 images from crowdsourcing and around 90 from the internet of Dosa and Upma/Vada-paav (Indian foods);  Amorphous food item dataset (e.g., takes the shape of the container)  Bowl and coin dataset | Newly created database with bowl and coin to serve as reference size | Two parts: Region Proposal Network (RPN) which is trained with ResNet (using Feature Pyramid Network FPN) to identify where food objects are in image, giving accuracy along with the processing speed in a Mask-RCNN; Fully Conventional Network segments the masks on each Region of Interest (ROI). | The volume is estimated by marking the coin and bowl with a bounding box; volume for amorphous food is done with a pixel per metric method to find the ratio of the pixel to the width of the reference object, the height and width of the object and what volume of food is in the bowl. | Volume to nutrients;  Blogger: Indian food calorie chart, URL http:// indianfoodrecipeswithpictures.blogspot.com/. |
| Li and Yang (2022) | China; Government | YOLOv5 | Other: Social robot that included a Kinect V2 depth camera, plus multiple cameras in the experimental environment | CFNet-34 = ChineseFoodNet + Images newly acquired | No | Food-recognition algorithm based on YOLOv5; input layer preprocesses the training dataset through the Mosaic data-enhancement method, adaptive anchor-frame calculation, adaptive picture scaling and other methods. Backbone layer divides the pictures through the Focus structure; Neck layer fuses data set through FPN and PAN operations to obtain the prediction feature map. The Precision layer calculates the gap between prediction box and real box through the loss function, updates parameters of the iterative model, filters the prediction box through the NMS operation to obtain results. | Calibrated the weight of food ingredients and designed the method for the calculation of food nutritional composition; then, proposed a dietary nutritional information autonomous perception method based on machine vision (DNPM) that supports the quantitative analysis of nutritional composition. Finally, the proposed algorithm was tested on the self-expanded dataset CFNet-34 based on the Chinese food dataset ChineseFoodNet. | Method: weight to nutrients (but unclear);  National Nutrition Database-Food Nutritional Composition Query Platform and Shi An Tong-Food Nutritional Composition Query Platform |
| Ma, et al. (2022) | China and USA; Government | Inception V3 work | NR | Internet websites  UECfood-256  Food-101 | NR | Deep CNN (4 types, trained the architecture on ImageNet) | Results were used with the ChinaMarketFood109 dataset for nutrient estimation. Three methods were compared: Method 1 used a straight arithmetic mean, Method 2 normalized the top 5% probabilities before calculating an arithmetic mean, and Method 3 used the top 5% probabilities for a harmonic mean. Grad-Cam was applied to differentiate correct and incorrect classifications using deep residual architecture for CNN-based features. | Nutrition in each type of food, weighted by the probability the image being a different type of food;  Dietary Guidelines for Chinese 2019 (http://dg.en.cnsoc.org/) the amount of 23 common nutrients for each class was annotated as ground truth value (table S1) |
| Minija and Emmanuel (2022) | India; NR | Imperialist Competitive Algorithm (IpCA)-based Deep Belief Network (IpCA-DBN) | NA | UNIMIB2016 dataset | No | Imperialist Competitive Algorithm (IpCA)-based Deep Belief Network (IpCA-DBN) for food category recognition and the calorie estimation of the food. Initially, the food image is pre-processed and subjected to the segmentation process, which is done by the Bayesian Fuzzy Clustering. Then, the features, such as shape, color histogram, wavelet, scattering transform features are generated from the optimal segments. | Features are fed to the IpCA-DBN for recognizing the food category and estimating the calorie of the food. | The calorie of the segments depends on the area of the segment, and the calorie value of the food and the area of the segment is the product of the number of the pixels and the unit equivalent of a pixel. The database seemed to be annotated with nutrient info |
| Pfisterer, et al. (2022) | Canada; Goverment | Deep convolutional neural network (DCNN) leveraged a spliced ResNet101 architecture | NA | UNIMIB2016: pre-labelled food dataset (1027 tray images, 73 categories), which contains per-pixel ground-truth segmentation  Regular foods dataset: custom long-term care (LTC), fully labelled, high resolution dataset with 9 unique foods (variety of fruits, vegetables, pastas, soup, and meat dishes) representative of LTC  Modified texture foods dataset: custom long-term care (LTC), fully labelled, high resolution dataset with 27 unique foods (variety of fruits, vegetables, pastas, soup, and meat dishes) representative of LTC | A single RGB-D (depth sensor) camera | Deep CNN for food segmentation maps, refined using depth heuristics and combined with a calibrated pixel-wise food heights to estimate food consumption -- an Encoder Decoder Food Network (EDFN) architecture that computes food volume and computes food intake.  PSPNet is the segmentation backbone; they used ResNet 101 architecture (trained on ImageNet) automatic semantic segmentation with depth refinement; | Proposed deep food segmentation network comprised of: (1) encoder-decoder food network (EDFN) consisting of a residual encoder microarchitecture and a pyramid scene parsing decoder microarchitecture which outputs a segmented food mask; (2) pixel height map estimation for assessing food depth; (3) depth refinement which outputs a depth-refined food mask (EDFN-D); and (4) food intake report summarizing the volume of food on the plate, the intake amount (in mL) and the intake percent. | NA |
| Prakash, et al. (2022) | India; Private incorporated and government | CNN | NA | FRUITS | No | CNN to process images and output multi-class labels and train the model | NA | Method: Volume to nutrients; FRUITS dataset  Routine health information from the  health management information system (HMIS) |
| Sasaki, et al. (2022) | Japan; Link & Communication Inc (Tokyo, Japan) | CALO mama app | Smart phone | CALO mama app database, which has food images and nutrient information including fresh food, self-made meals, ready meals, and commercial products | NR | The CALO mama app is an automated image recognition system involving deep learning predicts food items from a list of the standard 215 items and identifies ingredients in each item and portion size. | Another system calculates the nutritional values of the items based on the predictions made by the image recognition system. Finally, the predicted names of the items, their portion sizes, and corresponding nutrition values are displayed on users' smartphones. If the outputs appear imprecise, users can manually search for appropriate food items from the full list of approximately 150,000 items, modify the name and portion size of each item, and record them. | Method: Portion to nutrients (e.g., 4 fries); Standard Tables of Food Composition in Japan |
| Tagi, et al. (2022) | Japan; Crowdfunding | YOLOv3; architecture of the multitask CNN based on Ege et al's (2017) method | Digital camera | FoodLog dataset | NR | Object-detection: YOLOv3 was used for object detection, following training using the FoodLog data set | Classification, matching food names, and estimation of leftover liquid food: The architecture of the multitask CNN involved a calorie-volume estimation model based on the method proposed by Ege et al | NA |
| UlHaque, et al. (2022) | Bangladesh; No external funding | Parameter-optimized CNN | NA | Food-101, subset of images from here  Fruit-360, subset of images from here | No | Image processing and segmentation: Used custom models of CNNs for dataset where different filter numbers and filter sizes were used. | Identify food items from the food image, measure volumes, match that information with the current nutritional fact table. Calorie estimation from calorie table based on detected food.  CNN or ConvNet based on classification epoch: 80 (from Fig. 1) | Direct estimate of nutrients from image  Food-101 and Fruit-360 had dietary data implicit in them, but the source of that data is not stated. Links provided in the reference section do not appear still to connect. |
| Zhang, et al. (2022) | China; NR | Aliyun Cloud Food Recognition API and Visual-inertial system | Smart phone | NA | No | Food classification: Aliyun Cloud Food Recognition API as food identification model to classify food. | Weight estimation: used deep convolutional neural network and Visual-inertial system as the Area-weight model to predict food weight. | NA |
| Nadeem, et al. (2023) | New Zealand and Kuwait; No external funding | single stage fast region-based CNN; TensorFlow Object Detection API designed by Google and the pretrained model: fast-er_rcnn_inception_v2_coco | Digital camera | NA | Other: NZD 2 NZD coin. The diameter of the coin was 2.65 CM. | Food recognition and classification: applied a pre-trained faster R-CNN model for food images classification. Images are classified by deploying a trained TensorFlow object detection model. | Volume calculation using image processing: The diameter of the coin as a reference was used to determine the pixel-to-cm ratio for computing the approximate volume. To determine the volume of an item from a 2D images, applied GrabCut algorithm as segmentation method.  Calorie estimation: The calories are estimated by first identifying the food in the image and then using volume estimation to obtain the relative size of the food to a NZD 2 coin reference object. The weight of the food is determined by multiplying its volume by its density, and the approximate calorie count can be calculated using the calories per 100 g for the specific food. | Volume to nutrients |
| Shao, et al. (2023) | China; Goverment | ResNet-101 was selected as the backbone network, and the study compared 6 basic backbone networks: AlexNet, VGG-16, Inception V3, ResNet-50, ResNet-101, CoTNet | NA | Nutrition5k dataset: RGB and depth images captured by an Intel RealSense D435 camera; more than 250 food categories; images annotated with nutritional information  Food2k dataset: a large food recognition dataset containing 2,000 categories and over one million images | RGB-D camera was used | Utilizing a ResNet-101 backbone network for feature extraction from RGB and depth images.  Employing a Feature Pyramid Network (FPN) to create multi-scale feature maps.  Incorporating a multimodal feature fusion (MMFF) module that includes components such as the Balanced Feature Pyramid (BFP) and Convolutional Block Attention Module (CBAM) for enhancing features.  Using the resulting feature maps for food detection and classification. |  | Direct estimate of nutrients from image; USDA Food and Nutrient Database |
| Zheng, et al. (2023) | China and USA; Government | Applications with Noise (DBSCAN) algorithm | Other: Aiptek iDV Stereo camera | None | Fiducial marker | Food and plate separation: food and plate segmentation by the Simple Linear Iterative Cluster (SLIC) and Density Based Spatial Clustering of Applications with Noise (DBSCAN) algorithm, The plate plane was determined by the Maximum Likelihood Estimation Sample Consensus (MLESAC) algorithm | The food volume estimation method utilizes stereo vision, multi-layer superpixel segmentation employing the Simple Linear Iterative Cluster algorithm (SLIC) in a 5-D space, including lightness scale, hue, saturation, and pixel coordinates. Disparity maps are generated using the Density-Based Spatial Clustering of Applications with Noise (DBSCAN). Based on the disparity information, the image is sliced sequentially in the depth direction and normalized to adjust for closer objects appearing larger. After normalizing pixels in all slices, the area of each slice is calculated and summed to determine the food volume. | Volume to nutrients |

FNDDS = Food and Nutrient Database for Dietary Studies from the United States Department of Agriculture (USDA); LED = light emitting diode; NA = Not applicable; NR = Not reported; SVM = support vector machine; PDA = personal digital assistant; 3D/2D = three dimensional/two dimensional; NN = neural network; CNN = computational neural network

^a^ Rhyner, et al. (2016) and Vasiloglou, et al. (2018) report on different studies using the same tool.

**eFigure 1**. Percent of total papers (n = 52) reporting volume, calories, carbohydrates, weight/mass or protein by type of results reported
